# A systematic review of match-play characteristics in women’s soccer

**DOI:** 10.1371/journal.pone.0268334

**Published:** 2022-06-30

**Authors:** Alice Harkness-Armstrong, Kevin Till, Naomi Datson, Naomi Myhill, Stacey Emmonds

**Affiliations:** 1 School of Sport, Rehabilitation and Exercise Sciences, University of Essex, Colchester, United Kingdom; 2 Institute for Sport, Physical Activity and Leisure, Leeds Beckett University, Leeds, United Kingdom; 3 Institute of Sport, University of Chichester, Chichester, United Kingdom; 4 The Football Association, Burton Upon Trent, United Kingdom; PLOS (Public Library of Science), UNITED KINGDOM

## Abstract

This review aimed to (1) systematically review the scientific literature evaluating the match-play characteristics of women’s soccer, (2) determine the methods adopted to quantify match-play characteristics of women’s soccer, and (3) present the physical, technical and tactical characteristics of women’s soccer match-play across age-groups, playing standards and playing positions. A systematic search of electronic databases was conducted in May 2021; keywords relating to the population, soccer and match-play characteristics were used. Studies which quantified physical, technical or tactical performance of women’s soccer players during match-play were included. Excluded studies included adapted match-play formats and training studies. Sixty-nine studies met the eligibility criteria. Studies predominantly quantified match-play characteristics of senior international (n = 27) and domestic (n = 30) women’s soccer match-play, with only seven studies reporting youth match-play characteristics. Physical (n = 47), technical (n = 26) and tactical characteristics (n = 2) were reported as whole-match (n = 65), half-match (n = 21), segmental (n = 17) or peak (n = 8) characteristics. Beyond age-groups, playing standard, and playing position, fourteen studies quantified the impact of contextual factors, such as environment or match outcome, on match-play characteristics. Distance was the most commonly reported variable (n = 43), as outfield women’s soccer players covered a total distance of 5480–11160 m during match-play. This systematic review highlights that physical match-performance increases between age-groups and playing standards, and differs between playing positions. However, further research is warranted to understand potential differences in technical and tactical match-performance. Coaches and practitioners can use the evidence presented within this review to inform population-specific practices, however, they should be mindful of important methodological limitations within the literature (e.g. inconsistent velocity and acceleration/deceleration thresholds). Future research should attempt to integrate physical, technical and tactical characteristics as opposed to quantifying characteristics in isolation, to gain a deeper and more holistic insight into match-performance.

## 1 Introduction

There has been substantial global growth and development of women’s soccer within recent years. Global, continental and national governing bodies have implemented specific women’s soccer strategies and increased investment, to support the development of the sport from grassroots to elite playing standards [1–5]. There has been an increase in participation rates [3], increased provision and support for developing talented youth players (e.g. the English Football Association’s regional talent centres and Women’s Super League academies programme), increased professionalisation of elite playing standards [6], and subsequently increased audiences for elite senior competitions (e.g. FIFA Women’s World Cup, UEFA Women’s European Championships, UEFA Champions League) [3, 5, 6]. Furthermore, recent research has suggested that observed increases in physical match-play performances of elite senior players are consequential of the sport’s growth and development, and increased professionalisation of the game [7, 8].

Additionally, there has been a notable increase in the volume of literature focusing on women’s soccer [9], which is likely reflective of the sport’s growth and development. The focus of the literature to date has predominantly surrounded injury and strength and conditioning of women’s soccer players, with limited research quantifying the match-characteristics of women’s soccer [9]. This is problematic, as knowledge and understanding of the demands which players may experience during match-play is important for informing population-specific practices for match-play and beyond. For example, coaching practice design and training programme design in preparation for the demands of match-play within respective playing standards, preparing players transitioning across playing standards, long-term athletic player development practices, talent identification, or injury monitoring and rehabilitation processes.

Despite a relatively limited body of literature, there have previously been six narrative reviews summarising match-play characteristics of women’s soccer [10–15]. However, there are several important limitations associated with these reviews. Firstly, without a comprehensive literature search and pre-defined, objective study selection criteria, narrative reviews may involve subjective study selection bias [16]. Additionally, the depth of information or choice of data extracted from respective studies may be limited or subjective. Consequentially, narrative reviews may result in biased or subjective author interpretation and conclusions [16]. Therefore, there is a need for a systematic review, to provide a comprehensive, objective and scientifically rigorous summary of the evidence-base on match-play characteristics of women’s soccer. Secondly, all narrative reviews to date have exclusively summarised the physical characteristics of match-play, neglecting the important technical and tactical characteristics. This is problematic, as soccer performance is the combination of physical, technical and tactical characteristics, and thus aspects of performance should not be considered in isolation [17, 18]. Therefore, there is a need to review and summarise physical, technical and tactical characteristics, to provide a holistic understanding of women’s soccer match-play. Thirdly, narrative reviews have highlighted methodological inconsistencies within the literature (e.g. methods of data collection, and velocity or acceleration thresholds). However, no review has attempted to evaluate the methodologies adopted to quantify match-play characteristics. Methods of data collection within recent research likely differ compared to earlier studies, due to FIFA law changes permitting wearable technology (e.g. global positioning system (GPS) units) within competitive match-play. Therefore, it is important that researchers and practitioners have an awareness and understanding of the different methodologies utilised within the literature when interpreting match-play characteristics and informing research or practice. Lastly, existing reviews neglected to summarise the peak periods of women’s soccer match-play characteristics [19, 20], which provide insight into the worst-case scenarios players may face during matches. Understanding the peak periods of match-play players may experience is important for informing coaching practice and training prescription for players, to ensure players are optimally prepared for the most demanding periods of match-play.

Therefore, the aims of this review were to: (1) systematically review the scientific literature evaluating the match-play characteristics of women’s soccer, (2) determine the methods adopted to quantify match-play characteristics of women’s soccer, and (3) present the physical, technical and tactical characteristics of women’s soccer match-play across age-groups, playing standards and playing positions. This will be the first systematic review of match-analysis within women’s soccer, providing researchers and practitioners with a comprehensive, critical and objective resource of the physical, technical, and tactical match-play research across women’s soccer populations, which can be used to inform respective population-specific practice.

## 2 Methods

### 2.1 Design and search strategy

The systematic review was conducted in accordance with the Preferred Reporting Items for Systematic Reviews and Meta-Analyses (PRISMA) statement [21]. A systematic search of electronic databases (CINAHL, Medline, PubMed, Scopus and SPORTDiscus) was completed on the 18^th^ May 2021, with no date restrictions applied. The search strategy included the terms for the population (‘female’ OR ‘women’s’ OR ‘girls’), AND sport (‘soccer’ OR ‘football’ OR ‘association football’), AND match-play characteristics (‘match characteristics’ OR ‘match demands’ OR ‘match performance’ OR ‘match play’ OR ‘match-play’ OR ‘match activities’ OR ‘activity profile’ OR ‘physical characteristics’ OR ‘physical performance’ OR ‘running characteristics’ OR ‘running demands’ OR running performance’ OR ‘peak demands’ OR ‘movement characteristics’ OR ‘movement profiles’ OR ‘technical characteristics’ OR ‘technical demands’ OR ‘technical performance’ OR ‘tactical characteristics’ OR ‘tactical demands’). Additionally, the search strategy included NOT (‘American football’ OR ‘Australian football’ OR ‘Australian rules football’ OR ‘Gaelic football’). Additional manual searches of selected study’s reference lists were conducted for potentially eligible studies. A review protocol was not prepared/registered prior to literature search.

### 2.2 Study selection

Duplicate studies were identified and eliminated prior to initial screening. Initial screening involved, two researchers independently (AHA, NM) screening the title, abstract, and keywords against the eligibility criteria. Selected studies’ reference lists were manually searched for other potentially eligible papers and included for further screening. Following initial screening, selected studies underwent full-text screening against the eligibility criteria, with the selected studies following this further screening included within this review. Disagreements by the two researchers following initial or full-text screening, were resolved through discussion.

Studies were included if they involved women’s soccer players, participants could be of any age, standard or playing position, and studies were included if they involved a physical, technical or tactical performance aspect of friendly or competitive match-play. Only peer-reviewed studies were included, with abstracts, book chapters, systematic reviews and theses excluded. Studies which only included; men, match-play characteristics of other football codes (i.e. American football, Australian rules football, futsal, Gaelic football, rugby league, rugby union, rugby sevens), quantification of training characteristics (i.e. did not include match-play), adapted match-play formats (i.e. match-play not in accordance with official rules for the respective age-group, e.g. reduced match duration or dimensions, small-sided games), or studies unavailable in English were also excluded.

### 2.3 Methodological quality

The methodological quality of the selected studies were assessed in line with previous systematic reviews involving match performance of soccer players [22, 23]. The methodological quality criteria are shown in Table 1. A maximum score of 10 out of 9 criteria questions could be obtained. Where ‘clearly’ is included within criteria, this required the relevant information to be explicitly detailed within the study. Methodological quality was included for descriptive purposes as opposed to criteria for inclusion/exclusion within this review.

**Table 1:** Methodological quality criteria for selected studies.

Question No.	Criteria	Score
Q1	The study is published in a peer-reviewed journal	No = 0, yes = 1
Q2	The study is published in an indexed journal	No = 0, yes = 1
Q3	The study objective(s) is/are clearly set out	No = 0, yes = 1
Q4	Either the number of recordings is specified or the distribution of players/recordings used is known	No = 0, yes = 1
Q5	The duration of player recordings (an entire half, a complete match etc.) is clearly indicated.	No = 0, yes = 1
Q6	A distinction is made according to player positions	No = 0, yes = 1
Q7	The reliability/validity of the instrument is not stated, is mentioned or is measured	Not stated = 0, mentioned = 1, measured = 2
Q8	Certain contextual variables (e.g. match status, match location, type of competition or the opponent) are taken into account in analysis or information is clearly specified	No = 0, yes = 1
Q9	The results are clearly presented	No = 0, yes = 1

### 2.4 Data extraction

Data were extracted by one author (AHA), and checked by a second (NM), with any disagreements resolved through discussion. Data relating to participant and study characteristics (e.g. age, height, body mass, standard of competition, number of teams, number of matches), methods of data collection and analysis (e.g. equipment specification, adopted velocity thresholds, variable definitions), and match-play characteristics (e.g. physical, technical or tactical variables, and match contextual information such as match outcome) were extracted. Where data were presented as figures, WebPlotDigitizer v4.4 [24] was utilised to extract data. Where studies included other data in addition to the relevant data, only the eligible data relating to match-play characteristics of women’s soccer players were extracted. For example, sex-differences [25–30], training and adapted match-formats [29, 31–33], matches against men’s soccer teams [34], or assessments of fitness or physiological characteristics [35, 36]. Lastly, to facilitate comparisons between studies, metrics were converted to standard units, including; player height (cm), distance covered (m), and relative distance covered (m·min^-1^).

### 2.5 Statistical analysis

A meta-analysis was precluded within this systematic review due to the variation in methods of data collection and analysis. Data are presented as mean ± SD. Where possible, any data extracted as mean ± SE or confidence intervals were converted to SD [19, 37–42], however, where this was not possible due to insufficient methodological information provided within studies, SE or confidence intervals were reported and noted [8, 26, 33, 43–46].

## 3 Results

### 3.1 Overview

**Fig 1:**
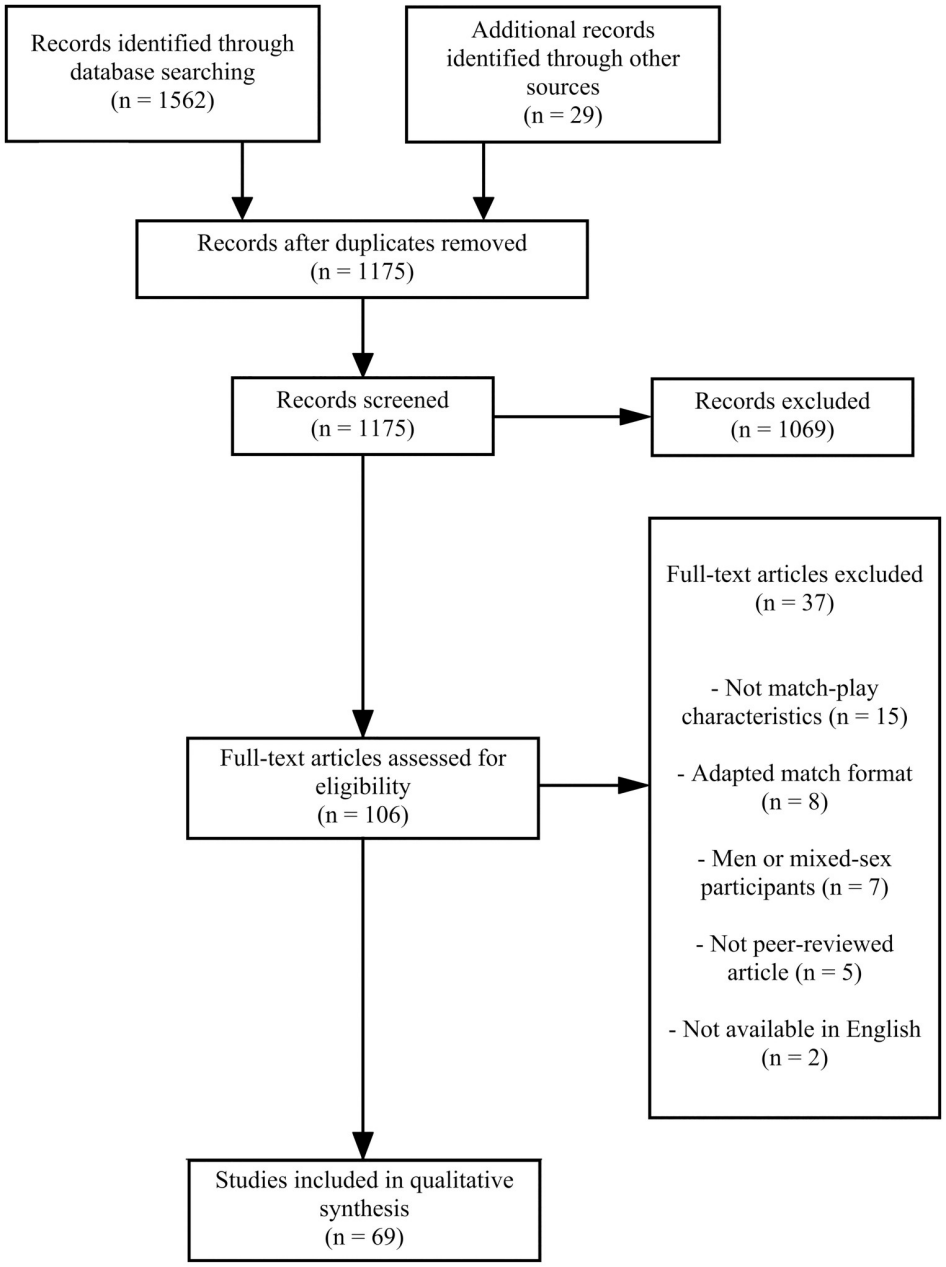
Flow diagram of study selection process for qualitative synthesis.

Fig 1 presents a flow diagram of the study selection process. The electronic database search identified 1562 articles, with an additional 29 articles identified through other sources. A total of 69 articles remained for analysis following removal of duplicates, initial and full-text screening [8, 19, 20, 25–90].

### 3.2 Study quality

The results for the methodological quality can be seen in Table 2. The mean score was 7.3 ± 1.4, and scores ranged between 4–10. The majority of studies lacked information regarding contextual variables (Q8 n = 28) of matches, whilst only 33 of the 69 studies differentiated match-play characteristics by playing position (Q6).

**Table 2:** Methodological quality of included studies.

Study	Question number									Total score
	Q1	Q2	Q3	Q4	Q5	Q6	Q7	Q8	Q9	
Alcock (2010) [47]	1	1	1	1	1	0	2	0	1	8
Althoff et al. (2010) [25]	1	1	1	1	1	0	0	0	1	6
Andersen et al. (2016) [31]	1	1	1	0	0	0	0	0	1	4
Andersson et al. (2010) [43]	1	1	1	1	1	1	2	0	1	9
Beare & Stone (2019) [48]	1	1	1	1	1	0	2	0	1	8
Bendiksen et al. (2013) [35]	1	1	1	0	0	0	0	1	1	5
Benjamin et al. (2020) [36]	1	1	1	1	1	1	0	0	1	7
Bohner et al. (2015) [49]	1	1	1	0	1	0	0	0	1	5
Bozzini et al. (2020) [50]	1	1	1	0	0	0	0	1	1	5
Bradley et al. (2014) [26]	1	1	1	0	1	1	1	0	1	7
Casal et al. (2021) [27]	1	1	1	1	1	0	2	0	1	8
Datson et al. (2017) [51]	1	1	1	1	1	1	1	0	1	8
Datson et al. (2019) [52]	1	1	1	1	1	1	1	0	1	8
De Jong et al. (2020) [53]	1	1	1	1	1	0	1	1	1	8
Gabbett et al. (2008) [34]	1	1	1	0	0	0	2	0	1	6
Gabbett et al. (2013) [54]	1	1	1	1	0	0	1	0	1	6
Garcia-Unanue et al. (2020) [55]	1	1	1	0	1	1	1	1	1	8
Gentles et al. (2018) [56]	1	1	1	1	0	1	1	0	1	7
Gómez et al. (2008) [28]	1	1	1	1	1	1	0	0	1	7
Griffin et al. (2021) [57]	1	1	1	1	1	1	1	1	1	9
Harkness-Armstrong et al. (2020) [37]	1	1	1	1	1	1	2	1	1	10
Harkness-Armstrong et al. (2021) [19]	1	1	1	1	1	1	1	1	1	9
Harriss et al. (2019) [58]	1	1	1	1	1	1	2	0	1	9
Hewitt et al. (2014) [38]	1	1	1	1	1	1	1	1	1	9
Hjelm (2011) [59]	1	1	1	1	1	0	0	0	1	6
Ibáñez et al. (2018) [60]	1	1	1	1	1	0	0	1	1	7
Ishida et al. (2021) [61]	1	1	1	1	0	0	1	0	1	6
Jagim et al. (2020) [62]	1	1	1	1	1	1	1	1	1	9
Julian et al. (2020) [63]	1	1	1	0	1	0	1	1	1	7
Konstadinidou & Tsigilis (2005) [64]	1	1	1	1	1	0	2	0	1	8
Krustrup et al. (2005) [65]	1	1	1	1	1	0	1	0	1	7
Krustrup et al. (2010) [66]	1	1	1	1	0	0	0	0	1	5
Kubayi & Larkin (2020) [67]	1	1	1	1	1	0	2	1	1	9
Mara et al. (2012) [68]	1	1	1	1	1	0	2	1	1	9
Mara et al. (2017) [69]	1	1	1	1	1	1	2	0	1	9
Mara et al. (2017) [70]	1	1	1	1	1	1	2	0	1	9
McCormack et al. (2015) [71]	1	1	1	0	1	0	0	1	1	6
McFadden et al. (2020) [29]	1	1	1	0	1	0	0	0	1	6
Meylan et al. (2017) [72]	1	1	1	1	1	0	0	1	1	7
Mohr et al. (2008) [44]	1	1	1	0	1	0	1	0	1	6
Nakamura et al. (2017) [73]	1	1	1	1	1	1	1	0	1	8
Ohlsson et al. (2015) [32]	1	1	1	1	1	0	1	1	1	8
Panduro et al. (2021) [74]	1	1	1	1	1	1	0	1	1	8
Park et al. (2019) [39]	1	1	1	1	1	0	1	1	1	8
Paulsen et al. (2018) [45]	1	1	1	0	0	1	1	0	1	6
Peek et al. (2021) [75]	1	1	1	1	1	1	2	1	1	10
Póvoas et al. (2020) [76]	1	1	1	0	1	0	0	1	1	6
Principe et al. (2021) [77]	1	1	1	0	0	1	1	0	1	6
Ramos et al. (2017) [78]	1	1	1	0	0	1	1	1	1	7
Ramos et al. (2019) [33]	1	1	1	0	1	1	0	0	1	6
Ramos et al. (2019) [79]	1	1	1	1	1	1	1	0	1	8
Romero-Moraleda et al. (2021) [80]	1	1	1	1	1	1	1	0	1	9
Sausaman et al. (2019) [81]	1	1	1	1	1	1	0	0	1	7
Scott et al. (2020) [8]	1	1	1	1	1	1	1	0	1	8
Scott et al. (2020) [82]	1	1	1	1	1	0	0	0	1	6
Soroka & Bergeir (2010) [83]	1	1	1	1	1	0	0	1	1	7
Tenga et al. (2015) [30]	1	1	1	0	1	0	0	1	1	7
Trewin et al. (2018) [20]	1	1	1	1	1	1	0	0	1	7
Trewin et al. (2018) [84]	1	1	1	1	1	0	1	1	1	8
Tscholl et al. (2007) [85]	1	1	1	0	0	0	2	0	1	6
Vescovi (2012) [86]	1	1	1	1	1	1	1	0	1	8
Vescovi (2014) [40]	1	1	1	1	1	0	1	0	1	7
Vescovi & Falenchuk (2019) [46]	1	1	1	0	1	0	1	1	1	6
Vescovi & Favero (2014) [41]	1	1	1	1	0	1	1	0	1	6
Wang & Qin (2020) [87]	1	1	1	1	1	1	1	1	1	9
Wang & Qin (2020) [88]	1	1	1	1	1	1	0	1	1	8
Wells et al. (2015) [89]	1	1	1	0	1	0	1	0	1	6
Williams et al. (2019) [42]	1	1	1	1	1	1	1	1	1	9
Zubillaga et al. (2013) [90]	1	1	1	0	1	0	0	0	1	5
**Total**	69	69	69	48	56	33	60	28	69	7.3

### 3.3 Participant and study characteristics

Table 3 presents the participant and study characteristics of the 69 studies. The earliest study was published in 2005 [64, 65]. There has been a notable increase in publications since 2015 (70%). Only 39 studies reported the year(s)/season(s) data was collected, of which 13 and 21 studies’ data were collected prior to- and since 2015, respectively, whilst 5 studies involved data collected both prior to- and since 2015. Nationalities of participants/locations of match-play included; Australia (n = 8; 12%), Brazil (n = 5; 7%), USA (n = 21; 30%), Canada (n = 1; 1%), and various Asian countries (n = 1; 1%), European countries (n = 24; 35%), or countries competing in the FIFA Women’s World Cup Finals (n = 9; 13%), whilst 3 studies did not report this information [20, 72, 84]. Studies predominantly quantified match-play characteristics of senior players (n = 63; 91%), and included international (n = 27; 39%), top tier domestic (n = 28; 41%), lower tiers domestic (n = 3; 4%), and college/university (n = 13; 19%) playing standards. Only seven studies involved youth players, including; U20 [78, 79], U17 [40, 75, 79], U16 [19, 37, 40, 75], U15 [40, 58, 75], U14 [19, 37, 58, 75] and U13 age-groups [58, 75]. Of the 53 studies which reported the number of teams, over half only involved a single team (n = 30; 57%). The mean number of reported participants was 52 (6–518), with 7 studies involving more than 100 participants (107–518) [8, 19, 37, 41, 51, 52, 55]. Of the 57 studies which reported number of matches, the mean number of matches observed was 38 (1–695). However, when excluding the largest number of matches observed within a single study (n = 695) [53], the mean reduced to 27 (1–230) matches. The majority of studies involved competitive match-play only, with two studies involving both competitive and friendly match-play [40, 57], three studies involving only non-competitive match-play [31, 39, 72], and two studies not stating whether match-play was competitive or friendly [20, 61]. Nineteen studies did not report the number of match files. The mean number of reported match files was 200 (4–3268), however when discarding the study with the largest number of match files (n = 3268) [8], the mean was reduced to 138 (4–695) match files.

**Table 3:** Participant and study characteristics of studies quantifying match-play characteristics of women’s soccer.

Study	Year(s) of Data Collection	Nationality/ Location	Age-Group	Playing Standard	No. of Teams	No. of Participants	No. of Matches	No. of Match Files	Data Inclusion	Age (yrs)	Height (cm)	Body Mass (kg)
Alcock (2010) [47]	2007	WWC	Senior	INT	NS	NS	32	32	All players	NS	NS	NS
Althoff et al. (2010) [25]	1999	WWC	Senior	INT	NS	NS	8	8	All players	NS	NS	NS
Andersen et al. (2016) [31]	NS	Denmark & Norway	Senior	DOM D1-3	3	27	1	NS	NS	21 ± 6	168.2 ± 1.5	61.0 ± 1.4
Andersson et al. (2010) [43]	NS	Denmark & Sweden	Senior	INT	2	17	3	54	WM	27 ± 1	170 ± 7	62 ± 7
				DOM D1	NS		3					
Beare & Stone (2019) [48]	2017–2018	England	Senior	DOM D1	NS	NS	89	89	All players	NS	NS	NS
Bendiksen et al. (2013) [35]	NS	Norway	Senior	DOM D2	1	11	1	NS	NS	21.0 ± 4.5	169.3 ± 5.5	58.7 ± 6.0
Benjamin et al. (2020) [36]	NS	USA	Senior	COL D1	1	14	26	199	>60-min	20.6 ± 1.4	169 ± 6.1	64.7 ± 5.3
Bohner et al. (2015) [49]	NS	USA	Senior	COL D1	1	6	3	NS	>60-min	19.5 ± 1.0	165.2 ± 5.5	62.1 ± 6.4
Bozzini et al. (2020) [50]	2018	USA	Senior	COL D1	1	11	NS	NS	45-min	19.0 ± 1.0	NS	68.1 ± 5.4
Bradley et al. (2014) [26]	NS	Europe	Senior	DOM UEFA CL	NS	59	NS	NS	WM	NS	NS	NS
Casal et al. (2021) [27]	2016–2017	Spain	Senior	DOM D1	14	NS	68	68	All players	NS	NS	NS
Datson et al. (2017) [51]	2011–2013	Europe	Senior	INT	13	107	10	148	WM	NS	NS	NS
Datson et al. (2019) [52]	2011–2013	Europe	Senior	INT	13	107	10	148	WM	NS	NS	NS
De Jong et al. (2020) [53]	2011–2018	Europe & USA & WWC	Senior	INT & DOM D1	NS	NS	695	695	All players	NS	NS	NS
Gabbett et al. (2008) [34]	NS	Australia	Senior	INT	1	13	12	NS	NS	21 ± 2	NS	NS
				DOM D1	1		9	NS				
Gabbett et al. (2013) [54]	NS	Australia	Senior	INT	1	13	5	15	NS	21 ± 2	NS	NS
				DOM D1	1		10	19				
Garcia-Unanue et al. (2020) [55]	2011	WWC	Senior	INT	16	205	NS	NS	>90-min	26.7 ± 4.2	NS	NS
	2015			INT	24	313				28.7 ± 5.2		
Gentles et al. (2018) [56]	NS	USA	Senior	COL D2	1	25	17	305	NS	20.2 ± 1.1	166.3 ± 5.9	62.0 ± 7.0
Gómez et al. (2008) [28]	2007	WWC	Senior	INT	NS	NS	13	13	All players	NS	NS	NS
Griffin et al. (2021) [57]	2016–2018	Australia	Senior	INT	1	18	15	97	WM	25.6 ± 3.7	166.7 ± 8.4	59.7 ± 6.8
				DOM D1	1	15	21	85		25.7 ± 3.1	167.5 ± 7.7	61.3 ± 6.2
Harkness-Armstrong et al. (2020) [37]	2018–2020	England	U16	DOM D1	6	108	21	210	Positional observation	15.0 ± 0.6	162.4 ± 5.9	56.1 ± 6.4
			U14		5	81	24	239		12.9 ± 0.7	158.7 ± 6.4	48.5 ± 8.9
Harkness-Armstrong et al. (2021) [19]	2018–2020	England	U16	DOM D1	6	108	26	204	Positional observation	15.0 ± 0.6	162.4 ± 5.9	56.1 ± 6.4
			U14		6	93	24	227		12.9 ± 0.7	158.7 ± 6.4	48.5 ± 8.9
Harriss et al. (2019) [58]	NS	Canada	U13-15	DOM	3	NS	60	60	All players	NS	NS	NS
Hewitt et al. (2014) [38]	NS	Australia	Senior	INT	1	15	13	58	WM	23.5 ± 0.7	170 ± 1	64.9 ± 1.3
Hjelm (2011) [59]	2003, 2007	Sweden	Senior	INT	1	NS	14	14	All players	NS	NS	NS
Ibáñez et al. (2018) [60]	2015–2016	Spain	Senior	DOM D1	16	NS	230	230	All players	NS	NS	NS
Ishida et al. (2021) [61]	NS	USA	Senior	COL D1	1	12	1	12	NS	20.7 ± 2.3	164.5 ± 6.0	64.4 ± 7.2
Jagim et al. (2020) [62]	2019	USA	Senior	COL D3	1	25	22	241	WM	19.7 ± 1.1	161 ± 30	66.7 ± 7.5
Julian et al. (2020) [63]	2015–2016	Germany	Senior	DOM D1-2	NS	15	NS	NS	>75-min	23 ± 4	169 ± 80	64.3 ± 8.2
Konstadinidou & Tsigilis (2005) [64]	1999	WWC	Senior	INT	4	NS	20	20	All players	NS	NS	NS
Krustrup et al. (2005) [65]	NS	Denmark	Senior	DOM D1	NS	14	4	14	WM	24	167	58.5
Krustrup et al. (2010) [66]	NS	Denmark	Senior	DOM D1	NS	23	3	23	NS	23	169	60.1
Kubayi & Larkin (2020) [67]	2019	WWC	Senior	INT	NS	NS	48	48	All players	NS	NS	NS
Mara et al. (2012) [68]	2010–2011	Australia	Senior	DOM D1	7	NS	34	34	All players	NS	NS	NS
Mara et al. (2017) [69]	NS	Australia	Senior	DOM D1	1	12	7	49	WM	24.3 ± 4.2	171.9 ± 5.1	65.3 ± 5.1
Mara et al. (2017) [70]	NS	Australia	Senior	DOM D1	1	12	7	49	WM	24.3 ± 4.2	171.9 ± 5.1	65.3 ± 5.1
McCormack et al. (2015) [71]	NS	USA	Senior	COL D1	1	10	16	NS	>45-min	20.5 ± 1.0	166.6 ± 5.1	61.1 ± 5.8
McFadden et al. (2020) [29]	NS	USA	Senior	COL D1	1	9	23	NS	>45-min	19.3 ± 1.4	166.6 ± 5.3	63.9 ± 5.7
Meylan et al. (2017) [72]	NS	NS	Senior	INT	1	13	34	157	WM	27.0 ± 5.3	170.3 ± 6.1	65.7 ± 5.3
Mohr et al. (2008) [44]	NS	USA	Senior	Top-Class (INT & DOM D1)	NS	19	2	NS	WM	NS	NS	NS
		Denmark & Sweden	Senior	High-Level (DOM D1)	NS	15	2	NS				
Nakamura et al. (2017) [73]	2015	Brazil	Senior	DOM D1	1	11	10	61	WM	21.0 ± 3.0	163.8 ± 4.5	59.7 ± 8.0
Ohlsson et al. (2015) [32]	NS	Sweden	Senior	DOM D1	3	15	1	15	>45-min	24 ± 3	167 ± 6	60 ± 4
Panduro et al. (2021) [74]	2019–20	Denmark	Senior	DOM D1	8	94	NS	108	WM	22.5 ± 4.2	170 ± 6	64.0 ± 6.1
Park et al. (2019) [39]	2012–2015	USA	Senior	INT	1	27	52	277	>45-min	24.6 ± 3.8	168.9 ± 4.8	63.0 ± 4.2
Paulsen et al. (2018) [45]	NS	USA	Senior	COL D1	1	21	13	NS	NS	18–23	NS	NS
Peek et al. (2021) [75]	2019	Australia	U13-17	DOM D1	55	NS	50	55	NS	NS	NS	NS
Póvoas et al. (2020) [76]	NS	Europe	Senior	INT	3	48	12	NS	NS	26 ± 4	170 ± 4	63.4 ± 4.8
Principe et al. (2021) [77]	2019	Brazil	Senior	DOM D1	1	23	23	NS	NS	27.7 ± 4.7	15.4 ± 5.8	60.9 ± 5.3
Ramos et al. (2017) [78]	2015	Brazil	U20	INT	1	12	7	NS	NS	18.0 ± 0.7	167 ± 5.8	62.0 ± 6.2
Ramos et al. (2019) [33]	2016	Brazil	Senior	INT	1	21	6	NS	>45min	26 ± 3.6	167 ± 5.8	NS
Ramos et al. (2019) [79]	2016	Brazil	Senior	INT	1	17	6	47	WM	27 ± 4.5	186.9 ± 4.8	60.7 ± 4.5
	2015		U20		1	14	7	54		18.1 ± 0.8	165.9 ± 6.8	59.9 ± 6.2
	2016		U17		1	14	7	43		15.6 ± 0.5	164.6 ± 6.4	58.0 ± 4.3
Romero-Morleda et al. (2021) [80]	2017–2018	Spain	Senior	DOM D1	1	18	NS	94	≥85% WM	26.5 ± 5.7	164.4 ± 5.3	58.6 ± 5.6
Sausaman et al. (2019) [81]	NS	USA	Senior	COL D1	1	23	NS	375	WM	20.6 ± 1.0	163.5 ± 13.3	62.1 ± 7.1
Scott et al. (2020) [8]	2016–2017	USA	Senior	DOM D1 (INT)	10	78	NS	1375	WM	25.0 ± 3.3	166.7 ± 6.1	64.0 ± 6.4
				DOM D1 (non-INT)		142	NS	1893				
Scott et al. (2020) [82]	2016–2017	USA	Senior	DOM D1	10	36	NS	408	WM	24.4*	168.2*	62.9*
Soroka & Bergeir (2010) [83]	2005	Europe	Senior	INT	NS	NS	15	15	All players	NS	NS	NS
Tenga et al. (2015) [30]	2003–2005	Spain	Senior	DOM D1	4	NS	4	4	All players	NS	NS	NS
Trewin et al. (2018) [20]	2012–2016	NS	Senior	INT	1	45	55	172	WM	NS	NS	NS
Trewin et al. (2018) [84]	2012–2015	NS	Senior	INT	1	45	47	606	>75-min	15–34	NS	NS
Tscholl et al. (2007) [85]	1999–2000, 2002–2004	WWC & Olympics	Senior & U19	INT	NS	NS	24	NS	NS	NS	NS	NS
Vescovi (2012) [86]	NS	USA	Senior	DOM D1	NS	71	12	139	WM	NS	NS	NS
Vescovi (2014) [40]	NS	USA	U17	DOM	NS	15	NS	15	WM	NS	NS	NS
			U16	DOM	NS	63	NS	63		NS	NS	NS
			U15	DOM	NS	11	NS	11		NS	NS	NS
Vescovi & Falenchuk (2019) [46]	NS	USA	Senior	DOM D1	NS	28	NS	NS	WM	NS	NS	NS
Vescovi & Favero (2014) [41]	NS	USA	Senior	COL D1	9	113	NS	117	>One half	NS	NS	NS
Wang & Qi (2020) [87]	2019	WWC	Senior	INT	24	NS	52	52	All players	NS	NS	NS
Wang & Qi (2020) [88]	2019	Asia	Senior	INT	4	NS	50	50	All players	NS	NS	NS
Wells et al. (2015) [89]	NS	USA	Senior	COL D1	1	9	21	NS	≥55-min	21.3 ± 0.9	170.3 ± 5.7	64.0 ± 5.8
Williams et al. (2019) [42]	NS	USA	Senior	COL D1	1	25	21	94	WM	19.3 ± 1.1	167.6 ± 5.6	63.0 ± 6.4
Zubillaga et al. (2013) [90]	NS	Spain	Senior	DOM D1	4	NS	4	4	All players	NS	NS	NS

NS = not specified. Nationality: WWC = Women’s World Cup. Age-Group: U = under. Playing Standard: COL = college, DOM = domestic, INT = international, D = division/tier. Match Files: WM = whole match.

* mean calculated from available data

### 3.4 Physical characteristics

Studies predominantly quantified physical characteristics of women’s soccer match-play (n = 47; 68%). The majority (n = 35; 74%) quantified whole-match absolute characteristics, whilst 21 studies (45%) quantified half-match absolute characteristics, 15 studies (32%) quantified segmental absolute values (e.g. 5-minutes, 15-minutes), 16 studies (34%) quantified whole-match relative values, and 8 studies (17%) quantified peak values. Distance was the most commonly quantified variable (n = 43; 91%). Details of data collection and analysis methods are presented in Table 4. Data collection methods for quantifying external load variables included; 5 Hz (n = 9; 19%), 10 Hz (n = 22; 47%) or 15 Hz (n = 1; 2%) GPS units, time-motion analyses (n = 5; 11%), 25 Hz multi-camera match analysis system (n = 3; 6%), 25 Hz optical player tracking system (n = 2; 4%), and 20 Hz radio-frequency tracking (n = 2; 4%). Heart rate monitors were used in 11 studies (23%), and the respective physical characteristics reported are presented in S1 Table.

**Table 4:** Methods used to quantify physical characteristics of women’s soccer match-play.

Study	Data Collection	Comparative Groups	Time-Period					Physical Characteristics
			W		H	S	P	
			A	R				
Andersen et al. (2016) [31]	20 Hz RF tracking (ZXY Sport Tracking System); HR monitor (Polar Team 2 System, Polar Electro OY)	N/A	Y	-	Y	Y	-	TD (km), TD (km) in velocity zones, accelerations (n), HIR and SPR bouts, mean and peak HR (BPM, % HR_max_)
Andersson et al. (2010) [43]	Video camera; time-motion analysis; HR monitor (Team System, Polar Electro OY)	*Playing Standard*: INT vs DOM D1 *Playing Position*: DEF vs MID vs FWD	Y	-	Y	Y	Y	TD (km), TD (km) in velocity zones, total time spent in velocity zones (%), frequency (n) and duration (s) of efforts, mean HR (BPM, % HR_max_)
Bendiksen et al. (2013) [35]	20 Hz RF tracking (ZXY Sport Tracking System)	N/A	Y	-	-	Y	-	TD (m), TD (m) in velocity zones
Benjamin et al. (2020) [36]	10 Hz GPS (Viper Pod, STATSports)	*Environmental Factors*: Low WBGT vs moderate WBGT vs high WBGT	-	Y	-	-	-	TD (m∙min^-1^), HSR (%/TD), High Metabolic Load (%)
Bohner et al. (2015) [49]	10 Hz GPS (MinimaxX 4.3, Catapult)	*Environmental Factors*: Sea-level vs altitude	-	Y	Y	-	-	TD (m∙min^-1^), TD (m∙min^-1^) in velocity zones
Bozzini et al. (2020) [50]	10 Hz GPS and HR monitor (Polar TeamPro; Polar Electro OY)	*Type of Competition*: In-conference vs out-of-conference	-	Y	Y	-	-	TD (m∙min^-1^), TD (m∙min^-1^) in velocity zones, SPR efforts (n∙min^-1^), time in heart rate zones (min∙min^-1^), energy expenditure (kcal∙min^-1^)
Bradley et al. (2014) [26]	25 Hz multi-camera match analysis system (Amisco Pro)	*Playing Position*: CD vs FB vs CM vs WM vs ATT	Y	-	Y	Y	Y	TD (m), TD (m) in velocity zones
Datson et al. (2017) [51]	25 Hz multi-camera match analysis system (STATS)	*Playing Position*: CD vs WD vs CM vs WM vs ATT	Y	-	-	Y	Y	TD (m), TD (m) in velocity zones, SPR; frequency (n), distance (m) and type (%)
Datson et al. (2019) [52]	25 Hz multi-camera match analysis system (STATS)	*Playing Position*: CD vs WD vs CM vs WM vs ATT	Y	-	-	-	-	Frequency (n) of efforts and bouts, distance (m) of efforts, recovery duration (s) between efforts and bouts
Gabbett et al. (2008) [34]	Video camera; time-motion analysis	*Playing Standard*: INT vs DOM D1	Y	-	Y	-	-	TD (m), TD (m) in velocity zones, time in velocity zones (%), frequency (n) and duration (s) of efforts. SPR; frequency (n), bouts (n), duration (s), recovery duration (s) and recovery movement (%)
Gabbett et al. (2013) [54]	Video camera; time-motion analysis	N/A	-	-	Y	Y	-	RHIA and RSA; frequency of bouts (n), efforts in bout (n), duration (s), recovery duration (s)
Gentles et al. (2018) [56]	5 Hz GPS (BT-Q1300ST, Qstarz International Co.)	N/A	Y	-	-	-	-	TD (km), TD (km) in velocity zones, impulse load (N∙s), RPE
Griffin et al. (2021) [57]	10 Hz GPS (SPI HPU, GPSports; VX Live Log, VX Sport)	*Playing Standard*: INT vs DOM D1 *Playing Position*: DEF vs MID vs ATT	Y	-	-	-	-	TD (m), TD (m) in velocity zones, and deceleration duration (s)
Harkness-Armstrong et al. (2021) [19]	10 Hz GPS (Optimeye S5; Catapult)	*Age-Group*: U14 vs U16 *Position*: CD vs WD vs CM vs WM vs FWD	Y	Y	-	-	Y	TD (m, m∙min^-1^), TD (m, m∙min^-1^) in velocity zones, maximum velocity (m∙s^-1^)
Hewitt et al. (2014) [38]	5 Hz GPS (MinimaxX v2.5; Catapult)	*Playing Position*: DEF vs MID vs ATT *Opposition Quality*: Ranked top 10 vs ranked 11–25 vs ranked >25	Y	-	Y	Y	-	TD (m), TD (m) in velocity zones, time spent SPR (%/TD)
Ishida et al. (2021) [61]	10 Hz GPS (Optimeye S5; Catapult)	N/A	Y	-	-	-	-	TD (m), TD (m) in velocity zones, PlayerLoad (au)
Jagim et al. (2020) [62]	10 Hz GPS and HR monitor (Polar TeamPro; Polar Electro, OY)	*Playing Position*: GK vs CB vs CM vs FP vs FWD	Y	-	-	-	-	TD (m), TD (m) in velocity zones, energy expenditure (kcals), mean HR (BPM, % HR_max_), SPR efforts (n), accelerations and decelerations (n)
Julian et al. (2020) [63]	5 Hz GPS (TT01, Tracktics GmbH)	*Stage of Menstrual Cycle*: follicular phase vs luteal phase	-	Y	-	-	-	TD (m∙min^-1^), TD (m∙min^-1^) in velocity zones, HSR and SPR bouts (n)
Krustrup et al. (2005) [65]	Video camera; time-motion analysis; HR monitor (Polar Vantage NC, Polar Electro OY)	N/A	Y	-	-	Y	-	TD (km), TD (km) in velocity zones, time spent in velocity zones (%), frequency (n) and duration (s) of efforts, mean HR (BPM)
Krustrup et al. (2010) [66]	HR monitor (Polar Vantage NC, Polar Electro OY)	N/A	Y	-	-	-	-	Mean HR (BPM, % HR_max_), peak HR (BPM, % HR_max_)
Mara et al. (2017) [69]	25 Hz optical player tracking system (Australian Institute of Sport)	*Playing Position*: CD vs WD vs MID vs WATT vs CATT	Y	-	Y	Y	-	Frequency (n), mean and maximum distance (m) per acceleration and deceleration effort, mean and maximum time (s) between efforts
Mara et al. (2017) [70]	25 Hz optical player tracking system (Australian Institute of Sport)	*Playing Position*: CD vs WD vs MID vs WATT vs CATT	Y	-	Y	Y	-	TD (m), TD (m) in velocity zones, frequency (n) mean and maximum distance (m) and duration (s) of HSR, RHSA, SPR, and RSPR efforts, recovery between efforts (s)
McCormack et al. (2015) [71]	10 Hz GPS (MinimaxX 4.0, Catapult)	*Match Congestion*: Previous match >42 hours vs <42 hours	-	Y	-	-	-	TD (m∙min^-1^), TD (m∙min^-1^) in velocity zones, HIR and SPR efforts (n)
McFadden et al. (2020) [29]	10 Hz GPS and HR monitor (Polar TeamPro; Polar Electro, OY)	N/A	Y	-	-	-	-	Average speed (km∙h^-1^), TD (km), TD (m) in velocity zones, SPR efforts (n), time spent in HR zones (min), energy expenditure (kcal)
Meylan et al. (2017) [72]	10 Hz GPS, 100 Hz accelerometer (MinimaxX S4, Catapult)	N/A	-	Y	-	-	-	TD (m∙min^-1^), TD (m∙min^-1^) in velocity zones, high-intensity efforts (n∙min^-1^), high inertial sensor count (n∙min^-1^), accelerations (n∙min^-1^)
Mohr et al. (2008) [44]	Video camera; time-motion analysis	*Playing Standard*: INT vs DOM D1 *Playing Position*: DEF vs MID vs FWD	Y	-	Y	Y	Y	TD (m), TD (m) in velocity zones, time spent in velocity zones (%), frequency (n) and duration (s) of efforts
Nakamura et al. (2017) [73]	5 Hz GPS (SPI Elite, GPSports Systems)	*Playing Position*: CD vs FB vs MID vs FWD	Y	-	Y	-	-	SPR; distance (m), frequency (n), duration (s), recovery between efforts (s)
Ohlsson et al. (2015) [32]	HR monitor (Polar Team 2 System, Polar Electro OY)	N/A	Y	-	Y	-	-	Mean HR (BPM, % HR_max_), peak HR (BPM, % HR_max_), time spent in HR_max_ zones (%)
Panduro et al. (2021) [74]	10 Hz GPS and HR monitor (Polar TeamPro; Polar Electro OY)	*Playing Position*: GK vs CD vs FB vs CM vs EM vs FWD	Y	-	Y	Y	Y	TD (m), TD (m) in velocity zones, peak speed (km∙h^-1^), mean and peak heart rate (BPM, % HR_max_), time spent in HR zones (min/min), accelerations and decelerations (n)
Park et al. (2019) [39]	10 Hz GPS (MinimaxX S4, Catapult)	N/A	-	-	Y	-	-	TD (m)
Principe et al. (2021) [77]	10 Hz GPS (Polar TeamPro; Polar Electro OY)	*Playing Position*: *DEF vs MID vs FWD*	-	-	Y	-	-	TD (m), TD (m) in velocity zones, accelerations and decelerations (n)
Paulsen et al. (2018) [45]	HR monitor (Polar Team 2 System, Polar Electro OY)	*Playing Position*: CD vs OD vs MID vs FWD	Y	-	-	Y	-	Mean HR (BPM)
Ramos et al. (2017) [78]	10 Hz GPS (MinimaxX,Team S5, Catapult)	*Playing Position*: CD vs WD vs MID vs FWD	Y	-	Y	Y	Y	TD (m), TD (m) in velocity zones, accelerations (n), decelerations (n), Player Load (au)
Ramos et al. (2019) [33]	10 Hz GPS (MinimaxX,Team S5, Catapult)	*Playing Position*: CD vs WD vs CM vs WM vs FWD	-	Y	-	-	-	TD (m∙min^-1^), TD (m∙min^-1^) in velocity zones, accelerations (n∙min^-1^), decelerations (n∙min^-1^), repeated accelerations/SPR (n∙min^-1^)
Ramos et al. (2019) [79]	10 Hz GPS (MinimaxX,Team S5, Catapult)	*Age-Group*: U17 vs U20 vs senior *Playing Position*: CD vs WD vs MID vs ATT	Y	-	-	-	-	TD (m), TD (m) in velocity zones, accelerations (n), decelerations (n), Player Load (au)
Romero-Moraleda et al. (2021) [80]	5 Hz GPS (SPI Pro X, GPSports Systems)	*Playing Position*: *CB vs WB vs CM vs WM vs ATT*	Y	Y	-	-	-	TD (m, m∙min^-1^), TD (m, m∙min^-1^) in velocity zones, accelerations and decelerations (n), body load (au), RPE
Sausaman et al. (2019) [81]	10 Hz GPS (NS, Catapult)	*Playing Position*: DEF vs MID vs ATT	Y	-	-	-	-	TD (m), TD (m) in velocity zones
Scott et al. (2020) [8]	10 Hz GPS (Optimeye S5, Catapult)	*Playing Standard*: DOM 1 (INT) vs DOM D1 (non-INT) *Playing Position*: GK vs CD vs WD vs CAM vs CDM vs WM vs FWD	Y	-	-	-	-	TD (m), TD (m) in velocity zones, maximum velocity (km/h)
Scott et al. (2020) [82]	10 Hz GPS (Optimeye S5, Catapult)	*N/A*	Y	-	-	-	-	TD (m), TD (m) in velocity zones
Trewin et al. (2018) [20]	10 Hz GPS (Optimeye S5, Catapult)	*Playing Position*: CD vs FB vs MID vs FWD	Y	Y	-	-	Y	TD (m, m∙min^-1^), TD (m, m∙min^-1^) in velocity zones, accelerations (n), HSR and SPR efforts (n), Player Load (au)
Trewin et al. (2018) [84]	10 Hz GPS (MinimaxX S4; Catapult)	*Environmental Factors*: Sea-level vs altitude, cold/mild vs warm/hot *Match Outcome*: Win vs draw vs loss *Opposition Quality*: ‘Win vs higher ranked’ vs ‘draw vs higher ranked’ vs ‘loss vs higher ranked’ vs ‘win vs lower ranked’ vs ‘draw vs lower ranked’ vs ‘loss vs lower ranked’*Match Congestion*: Previous match >72 hours vs <72 hours	-	Y	-	-	-	TD (m∙min^-1^), TD (m∙min^-1^) in velocity zones, accelerations (n∙min^-1^), HSR and SPR efforts (n∙min^-1^)
Vescovi (2012) [86]	5 Hz GPS (SPI Pro, GPSports)	*Playing Position*: DEF vs MID vs FWD	Y	-	Y	-	-	SPR; distance (m, %/TD), duration (s), time between efforts (s), maximum velocity (km∙h^-1^)
Vescovi (2014) [40]	5 Hz GPS (SPI Pro, GPSports)	*Age-Group*: U15 vs U16 vs U17 *Playing Position*: DEF vs MID vs FWD	Y	Y	Y	-	-	TD (m, m∙min^-1^), TD (m) in velocity zones, maximum velocity (m∙min^-1^), SPR; frequency (n) and distance (m)
Vescovi & Falenchuk (2019) [46]	5 Hz GPS (SPI Pro, GPSports)	*Match Location*: Home vs away *Type of Surface*: Natural vs artificial *Match Outcome*: Win vs draw vs loss	-	Y	-	-	-	TD (m∙min^-1^) in velocity zones, distance at Metabolic Power (m∙min^-1^); low (<20 W∙kg^-1^), high (20–35 W∙kg^-1^), elevated (35–55 W∙kg^-1^), maximal (>55 W∙kg^-1^)
Vescovi & Favero (2014) [41]	5 Hz GPS (SPI Pro, GPSports)	*Playing Position*: DEF vs MID vs FWD	-	-	Y	-	-	TD (m, m∙min^-1^), TD (m) in velocity zones
Wells et al. (2015) [89]	10 Hz GPS (MinimaxX 4.0, Catapult)	*Stage of Season*: Regular-season vs post-season	Y	Y	Y	-	-	TD (m, m∙min^-1^), TD (m, m∙min^-1^) in velocity zones, time in velocity zones (min,%), maximum velocity (km∙h^-1^), energy cost (kJ∙kg^-1^), exertion index (au∙min^-1^), PlayerLoad (au)
Williams et al. (2019) [42]	15 Hz GPS (SPI HPU, GPSports); HR monitor (T34, Polar Electro OY)	N/A	Y	-	-	Y	-	TD (m), High Metabolic Power (m), Speed Exertion (au), mean HR (BPM), HR exertion (au), Energy Expenditure (kJ/kg)

Data Collection: GPS = global positioning system; HR = heart rate; RF = radio-frequency. Comparative Groups: INT = international; DOM = domestic; D = division; U = under; GK = goalkeeper; DEF = defender; CB = centre back; CD = central defender; OD = outside defender; WD = wide defender; FB = full-back; MID = midfield; CM = central midfield; CAM = central attacking midfield; CDM = central defensive midfield; WM = wide midfield; ATT = attacker; CATT = central attacker; WATT = wide attacker; FP = flank players; FWD = forward. WBGT = wet bulb-globe temperature. Time-period: W = whole-match; A = absolute; R = relative; H = half-match; S = segmental; P = peak. Physical Characteristics: TD = total distance; HSR = high-speed running; HIR = high-intensity running; SPR = sprinting; RHIA = repeated high-intensity activity; RHSR = repeated high-speed running; RSA = repeated-sprint activity; RSPR; repeated-sprinting; RPE = rate of perceived exertion.*Originally expressed as m·s^-1^

The majority of studies involved comparative groups (n = 34; 72%); playing position (n = 25; 53%), playing standard (n = 5, 11%), and age-group (n = 3; 6%). Whilst, 9 studies (19%) quantified the impact of contextual variables on physical characteristics; environmental factors (e.g. altitude, temperature [36, 49, 84]), quality of opposition [38, 84], match outcome [46, 84], type of competition [50], match location [46], congestion of fixtures [71, 84], playing surface [46], stage of season [89], and stage of menstrual cycle [63].

Of the 26 studies which categorised players by playing position; 9 studies utilised high-level categorisation (i.e. defenders vs midfielders vs forwards) [38, 40, 41, 43, 44, 57, 77, 81, 86]; 7 studies differentiated central and wide defenders and midfielders (i.e. central defenders vs wide defenders vs central midfielders vs wide midfielders vs forwards) [19, 26, 33, 51, 52, 74, 80]; 5 studies differentiated central and wide defenders only (i.e. central defenders vs wide defenders vs midfielders vs forwards) [20, 45, 73, 78, 79]; 2 studies differentiated central and wide defenders and forwards/attackers (i.e. central defenders vs wide defenders vs midfielders vs central attackers vs wide attackers) [69, 70]; 1 study categorised wide players together (i.e. central defenders vs central midfielders vs wide players vs forwards) [62]; and 1 study differentiated central midfielders (i.e. central defenders vs wide defenders vs central attacking midfielders vs central defensive midfielders vs wide midfielders vs forwards) [8]. Three studies included goalkeepers within analysis [8, 62, 74].

A variety of velocity thresholds have been adopted within the 40 studies which categorised movement into velocity zones. The quantitative velocity thresholds are presented in Table 5. Four studies also quantified backwards running (>10 km·h^-1^) [35, 43, 44, 65]. The methods for establishing or adopting velocity thresholds included; arbitrary velocity thresholds which have previously been utilised in men’s soccer literature [26, 35, 43, 44, 51, 52, 65, 81, 89], sample-mean or individualised velocity thresholds derived from physical performance characteristics (e.g. sprint speed and maximal aerobic speed [20, 63, 72, 73, 84], velocity thresholds based on physical performance characteristics of women’s soccer players from existing literature [29, 33, 40, 41, 46, 57, 78, 79], derived velocity thresholds from match-play data of senior women’s soccer players [8, 38, 39, 70, 82], or a justification for velocity thresholds adopted was not provided [31, 49, 40, 56, 61, 62, 71, 73, 74, 77, 80, 86]. Additionally, 2 studies [34, 54], established velocity zones based on qualitative movement descriptors which had previously been utilised in men’s sports outside of soccer (e.g. hockey, rugby).

**Table 5:** Velocity thresholds (km∙h^-1^) adopted by selected studies utilising quantitative velocity zones to quantify physical characteristics of women’s soccer match-play.

Study	Standing	Walking	Jogging	Running	LSR / LIR	MSR / MIR	HSR / HIR	VHSR	Sprinting	No Descriptor	Additional
Andersen et al. (2016) [31]	-	< 7	7–12	12.1–16	-	-	16.1–20	-	>20	-	-
Andersson et al. (2010) [43]	0–6	6–8	8–12	-	12–15	15–18	18–25	-	>25	-	HIR >15
Bendiksen et al. (2013) [35]	-	0–8	8–12	-	12–15	15–18	18–21	-	>21	-	HIR >15
Bohner et al. (2015) [49]	0–2.02*	2.02–6.98*	6.98–9*	-	9–12.99*	13–15.98*	15.98–21.99	-	>22.0*	-	HIR >15.98
Bozzini et al. (2020) [50]	-	-	-	-	-	-	15.0–19.9	-	>20	-	-
Bradley et al. (2014) [26]	-	-	-	-	-	-	>15	-	-	0–12, 12–15, 15–18, 18–21, 21–23, 23–25, 25–27, >27	>12, >18
Datson et al. (2017) [51]	-	0.7–7.1	7.2–14.3	14.4–19.7	-	-	19.8–25.1	>19.8	>25.1	-	HSR >14.4
Datson et al. (2019) [52]	-	-	-	-	-	-	>19.8	-	>25.1	-	-
Gentles et al. (2018) [56]	1–4.99	5–9.99	-	-	10–14.99	-	15–19.99	20–24.99	>25	-	-
Griffin et al. (2021) [57]	-	-	-	-	-	-	16–20	-	>20	-	-
Harkness-Armstrong et al. (2021) [19]	-	-	-	-	-	-	>12.5	>19.0	>22.5	-	-
Hewitt et al. (2014) [38]	0–0.4	0.5–6	6–12	12–19	-	-	>12	-	>19	-	-
Ishida et al. (2021) [61]	-	-	-	-	-	-	>15	-		-	-
Jagim et al. (2020) [62]	-	<6.99	7.0–14.99	15.0–18.99	-	-	>15	-	>19	-	-
Juilian et al. (2020)	-	-	-	-	<13.2 ± 0.7	-	13.2 ± 0.7–16.69 ± 1.1	16.69 ± 1.1–19.94 ± 0.9	>19.94 ± 0.9	-	-
Krustrup et al. (2005) [65]	0–6	6–8	8–12	-	12–15	15–18	18–25	-	>25	-	HIR >15
Mara et al. (2017) [70]	-	-	-	-	-	-	12.24–19.44*	-	>19.44*	-	-
McCormack et al. (2015) [71]	-	-	-		-	-	12.99–21.99*	-	>21.99*	-	-
McFadden et al. (2020) [29]	-	-	-	-	-	-	15–18.99	-	>19	-	3–6.99, 7–10.99, 11–14.99
Meylan et al. (2017) [72]	-	-	-	-	-	-	16.5–19.9	-	>20	-	-
Mohr et al. (2008) [44]	0–6	6–8	8–12	-	12–15	15–18	18–25	-	>25	-	HIR >15
Nakamura et al. (2017) [73]	-	-	-	-	-	-	-	-	>20	-	IND SPR 19.37 ± 0.48
Panduro et al. (2021) [74]	-	-	-	-	-	-	>15	>18	>25	-	0–5.99, 6–11.99, 12–14.99, 15–17.99, 18–24.99
Park et al. (2019) [39]	-	-	-	-	-	-	12.5–19	19–22.5	>22.5	-	-
Principe et al. (2021) [77]	-	<11.99*	11.99–15.98*	15.99–19.98*	-	-	-	-	>19.98*	-	-
Ramos et al. (2017) [78]	-	-	-	-	-	-	15.6–20	-	>20	-	-
Ramos et al. (2019) [33]	0–6	-	-	6.1–8	8.1–12	12.1–15.5	15.6–20	-	>20	-	-
Ramos et al. (2019) [79]	-	-	-	-	-	-	15.6–20	-	>20	-	-
Romero-Moraleda et al. (2021) [80]	-	-	-	-	-	-	>15	-	-	-	-
Sausaman et al. (2019) [81]	0–0.1	0.1–6	6.1–8	-	8.1–12	12.1–15	15.1–18	-	18.1–25	-	HSR >15, SPR >18, Maximal SPR >25
Scott et al. (2020) [8]	-	-	-	-	-	-	>12.5	>19	>22.5	-	-
Scott et al. (2020) [82]	-	-	-	-	-	-	>12.5	>19	>22.5	-	-
Trewin et al. (2018) [20]	-	-	-	-	-	-	>16.48*	-	>19.98*	-	-
Trewin et al. (2018) [84]	-	-	-	-	-	-	>16.48*	-	>19.98*	-	-
Vescovi (2012) [86]	-	-	-	-	-	-	-	-	18–20.9, 21–22.9, 23–24.9, ≥25	-	>18 >21 >23
Vescovi (2014) [40]	-	0–6	6.1–8	-	8.1–12	12.1–15.5	15.6–20	-	>20	-	-
Vescovi & Falenchuk (2019) [46]	-	≤6	6.1–8	-	8.1–12	12.1–16	16.1–20	-	20.1–32	-	-
Vescovi & Favero (2014) [41]	-	0–6	6.1–8	-	8.1–12	12.1–15.5	15.6–20	-	>20	-	-
Wells et al. (2015) [89]	0–1.98*	1.99–6.95*	6.96–8.96*	-	8.97–12.99*	13–15.95*	15.96–21.9*	-	≥22.0	-	HIR: >13

LSR = low-speed running; LIR = low-intensity running; MSR = moderate-speed running; MIR = moderate-intensity running; HSR = high-speed running; HIR = high-intensity running; VHSR = very-high-speed running; SPR = sprinting; IND = individualised.

*Converted to km·h^-1^ from m·s^-1^

Fourteen studies quantified acceleration and/or deceleration, however, studies predominantly provided no justification for the thresholds adopted (>1 m·s^-2^ [33, 79]; >2 m·s^-2^ [31, 77, 78]) [54, 64, 70]. Where a rationale was provided, thresholds were either; derived from physical performance characteristics of the sample (e.g. acceleration during a maximal sprint; >2.26 m·s^-2^) [20, 72, 84] or aligned to previous men’s soccer literature (>2 m·s^-2^) [57, 69]. Five of these studies presented accelerations and/or decelerations within acceleration/deceleration zones, however all studies adopted different thresholds (<1, >1 m·s^-2^ [80]; 1–2, >2 m·s^-2^ [77]; 0.5–1.99, 2–2.99, >3 m·s^-2^ [62]; 0.5–1.49, 1.5–2.99, >3 m·s^-2^ [74]; 1–2, 2–3, 3–4, >4 m·s^-2^ [57]).

#### 3.4.1 Whole-match physical characteristics

The majority of studies quantifying physical characteristics quantified whole-match absolute values (n = 33; [8, 20, 26, 29, 31, 32, 34, 35, 38–40, 42–45, 51, 52, 56, 57, 61, 62, 65, 66, 69, 70, 73, 74, 78–81, 86, 89]. Table 6 presents whole-match absolute values of the most frequently reported physical characteristics (i.e. total distance (TD); TD in velocity zones (high-speed running (HSR), very-high-speed running (VHSR), and sprinting (SPR)), maximum velocity, number of accelerations and decelerations). Whilst S2 and S3 Tables present the specific HSR and SPR characteristics (i.e. number of efforts and repeated efforts, distance, duration, recovery duration), and acceleration and deceleration characteristics (i.e. number of efforts, total duration), respectively. In addition to the physical characteristics presented, studies quantified the number of game activities or (i.e. the total number of individual efforts across all velocity zones; 1326–1641) [43, 44, 65], and percentage of game activity for HSR (3.7–24%) [34, 43, 44, 51, 65] and SPR (0.54–2.7%) [34, 43, 44, 51] for senior international and domestic players.

**Table 6:** Studies quantifying physical characteristics of women’s soccer match-play per whole-match as absolute data.

Study	Sample/Group			Velocity (km∙h^-1^) and Acceleration (m∙s^-2^) Thresholds	Playing Position	TD (m)	HSR (m)	VHSR (m)	SPR (m)	Vmax (km∙h^-1^)	ACC (n)	DEC (n)
Andersen et al. (2016) [31]	DOM D1-D3			HSR: 16.1–20 SPR: >20 ACC: >2	All	10400 ± 800	1436 ± 308	-	498 ± 15	-	161 ± 31	-
Andersson et al. (2010) [43]	INT			HSR: >15 SPR: >25	All	9900 ± 1800*	1530 ± 100*	-	256 ± 57*	-	-	-
					DEF	9500 ± 900*	1310 ± 100*	-	221 ± 32*	-	-	-
					MID	10600 ± 300*	1900 ± 200*	-	316 ± 51*	-	-	-
					FWD	9800 ± 200*	1620 ± 120*	-	262 ± 46*	-	-	-
	DOM D1				All	9700 ± 1400*	1330 ± 900	-	221 ± 45*	-	-	-
					DEF	9500 ± 100*	1250 ± 130*	-	230 ± 33*	-	-	-
					MID	10100 ± 300*	1480 ± 160*	-	221 ± 39*	-	-	-
					FWD	9500 ± 500*	1360 ± 200*	-	191 ± 42*	-	-	-
Bendiksen et al. (2013) [35]	DOM D2			HSR: >15 SPR: >21	All	9674 ± 191	1193 ± 115	-	372 ± 46	-	-	-
Bradley et al. (2014) [26]	DOM UEFA CL			HSR: >15	All	10754**	777 ± 33	-	-	-	-	-
					CD	10238**	602 ± 41	-	-	-	-	-
					FB	10706**	756 ± 86	-	-	-	-	-
					CM	11160**	778 ± 46	-	-	-	-	-
					WM	10929**	931 ± 78	-	-	-	-	-
					ATT	10766**	1051 ± 78	-	-	-	-	-
Datson et al. (2017) [51]	INT			HSR: 19.8–25.1 VHSR: >19.8 SPR: >25.1	All	10321 ± 859	2520 ± 580	776 ± 247	168 ± 82	-	-	-
					CD	9489 ± 562	1901 ± 268	534 ± 113	111 ± 42	-	-	-
					WD	10250 ± 661	2540 ± 500	796 ± 237	163 ± 79	-	-	-
					CM	10985 ± 706	2882 ± 500	853 ± 229	170 ± 69	-	-	-
					WM	10623 ± 665	2785 ± 510	920 ± 260	220 ± 116	-	-	-
					ATT	10262 ± 798	2586 ± 463	872 ± 161	221 ± 53	-	-	-
	INT		In possession		All	-	-	313 ± 210	-	-	-	-
					CD	-	-	103 ± 48	-	-	-	-
					WD	-	-	309 ± 161	-	-	-	-
					CM	-	-	311 ± 197	-	-	-	-
					WM	-	-	485 ± 195	-	-	-	-
					ATT	-	-	530 ± 127	-	-	-	-
			Out of possession		All	-	-	399 ± 143	-	-	-	-
					CD	-	-	371 ± 100	-	-	-	-
					WD	-	-	418 ± 120	-	-	-	-
					CM	-	-	485 ± 163	-	-	-	-
					WM	-	-	366 ± 166	-	-	-	-
					ATT	-	-	274 ± 114	-	-	-	-
Gabbett et al. (2008) [34]	INT			Qualitative	All	9968 ± 1143	2461 ± 491	-	965 ± 305	-	-	-
	DOM D1				All	9706 ± 484	2014 ± 301	-	NS	-	-	-
Gentles et al. (2018) [56]	COL D2			HSR: 15–19.99 VHSR: 20–24.99 SPR: >25	All	5480 ± 2350	460 ± 250	110 ± 80	20 ± 20	-	-	-
Griffin et al. (2021) [57]	INT			HSR: 16–20 SPR: >20	All	9433 ± 263	766 ± 64	-	364 ± 53	-	-	-
	DOM D1				All	8728 ± 283	609 ± 9	-	306 ± 56	-	-	-
Harkness-Armstrong et al. (2021) [19]	U16 DOM D1			HSR: >12.5 VHSR: >19 SPR: >22.5	All	7679 ± 2114	1696 ± 886	249 ± 143	53 ± 57	24.8 ± 1.5	-	-
					CD	6954 ± 1218	1308 ± 583	204 ±136	41 ± 45	24.5 ± 1.6	-	-
					WD	7603 ± 1210	1729 ± 576	277 ± 134	62 ± 44	25.1 ± 1.6	-	-
					CM	8385 ± 1376	1689 ± 648	124 ± 153	17 ± 51	23.8 ± 1.8	-	-
					WM	7934 ± 1218	2023 ± 583	326 ± 136	75 ± 52	25.5 ± 1.6	-	-
					FWD	7516 ± 1020	1728 ± 505	316 ± 122	72 ± 36	25.3 ± 1.7	-	-
	U14 DOM D1				All	7148 ± 2215	1530 ± 934	188 ± 151	29 ± 60	24.0 ± 1.6	-	-
					CD	6603 ± 1195	1246 ± 576	188 ± 139	33 ± 44	24.3 ± 1.8	-	-
					WD	6905 ± 1288	1471 ± 609	183 ± 147	25 ± 49	23.9 ± 1.8	-	-
					CM	7790 ± 1429	1609 ± 672	116 ± 156	13 ± 62	23.0 ± 2.0	-	-
					WM	7472 ± 1210	1742 ± 583	202 ± 141	30 ± 45	24.2 ± 1.6	-	-
					FWD	6962 ± 1158	1584 ± 558	249 ± 132	43 ± 48	24.6 ± 1.9	-	-
Hewitt et al. (2014) [38]	INT			HSR: >12 SPR: >19	ALL	9631 ± 1332	2407 ± 952	-	338 ± 228	-	-	-
					DEF	8759 ± 1024	1744 ± 498	-	188 ± 112	-	-	-
					MID	10150 ± 1243	2797 ± 953	-	392 ± 252	-	-	-
					ATT	9442 ± 1379	2272 ± 794	-	388 ± 217	-	-	-
Ishida et al. (2021) [61]	COL D1			HSR: >15	All	10036 ± 5206	1049 ± 525	-	-	-	-	-
Jagim et al. (2020) [62]	COL D3			HSR: >15 SPR: >19 ACC: >2	All	9793 ± 2715	1019 ± 560	-	282 ± 205	-	74**	85**
					GK	5622 ± 1953	48 ± 31	-	7 ± 15	-	29**	26**
					CD	9956 ± 2511	1004 ± 417	-	309 ± 163	-	78**	86**
					CM	10575 ± 511	1145 ± 388	-	266 ± 117	-	80**	88**
					FP	10056 ± 2763	1264 ± 613	-	403 ± 258	-	80**	90**
					FWD	7831 ± 2180	798 ± 308	-	140 ± 65	-	58**	65**
Krustrup et al. (2005) [65]	DOM D1			HSR: >15 SPR: >25	All	10300	1310	-	160	-	-	-
Mara et al. (2017) [69]	DOM D1			ACC: >2 DEC: <-2	All	-	-	-	-	-	423 ± 126	430 ± 125
					CD	-	-	-	-	-	342**	356**
					WD	-	-	-	-	-	431**	443**
					MID	-	-	-	-	-	465**	473**
					CATT	-	-	-	-	-	413**	409**
					WATT	-	-	-	-	-	475**	474**
Mara et al. (2017) [70]	DOM D1			HSR: 12.24–19.44* SPR: >19.44*	All	10025 ± 775	2452 ± 636	-	615 ± 258	-	-	-
					CD	9220 ± 590	1772 ± 439	-	417 ± 116	-	-	-
					WD	10203 ± 568	2569 ± 612	-	680 ± 278	-	-	-
					MID	10581 ± 221	2761 ± 417	-	484 ± 169	-	-	-
					CATT	9661 ± 602	2420 ± 405	-	841 ± 238	-	-	-
					WATT	10472 ± 878	2917 ± 545	-	850 ± 178	-	-	-
McFadden et al. (2020) [29]	COL D1			HSR: 15–18.99 SPR: >19	All	8310 ± 900	812 ± 88	-	401 ± 158	-	-	-
Mohr et al. (2008) [44]	Top-class			HSR: >15 SPR: >25	All	10330 ± 150*	1680 ± 90*	-	460 ± 20*	-	-	-
	High-level				All	10440 ± 150*	1300 ± 100*	-	380 ± 50*	-	-	-
	Top-class & high-level				DEF	10200 ± 100*	1260 ± 110*	-	330 ± 50*	-	-	-
					MID	10610 ± 190*	1650 ± 110*	-	430 ± 40*	-	-	-
					ATT	10200 ± 200 *	1630 ± 100*	-	520 ± 30*	-	-	-
Nakamura et al. (2017) [73]	DOM D1			SPR: >20	All	-	-	-	285 ± 164	-	-	-
					CD	-	-	-	125 ± 61	-	-	-
					FB	-	-	-	359 ± 98	-	-	-
					MID	-	-	-	359 ± 174	-	-	-
					FWD	-	-	-	352 ± 145	-	-	-
				SPR: >19.37 ± 0.48	All	-	-	-	353 ± 206	-	-	-
					CD	-	-	-	150 ± 71	-	-	-
					FB	-	-	-	496 ± 136	-	-	-
					MID	-	-	-	372 ± 192	-	-	-
					FWD	-	-	-	493 ± 179	-	-	-
Panduro et al. (2021) [74]	DOM D1			HSR: >15 VHSR: >18 SPR: >25 ACC: >3 DEC: <-3	GK	5214 ± 949	99 ± 70	31 ± 31	1 ± 3	21.5 ± 1.2	2.8 ± 1.5	3.3 ± 1.3
					CD	9274 ± 762	1088 ± 261	442 ± 135	19 ± 17	27.5 ± 2.3	6.7 ± 3.7	13 ± 4.3
					FB	10053 ± 639	1529 ± 369	717 ± 242	46 ± 48	28.2 ± 3.2	8.0 ± 4.9	17 ± 4.6
					CM	10572 ± 880	1518 ± 499	623 ± 252	33 ± 31	27.8 ± 2.0	10 ± 6.8	16 ± 5.5
					WM	10519 ± 963	1786 ± 527	863 ± 299	91 ± 81	27.6 ± 2.1	7.1 ± 5.4	23 ± 6.7
					FWD	9745 ± 988	1561 ± 372	737 ± 223	56 ± 45	29.2 ± 3.2	12 ± 7.0	19 ± 3.9
Ramos et al. (2017) [78]	INT U20			HSR: 15.6–20 SPR: >20 ACC: >2 DEC: <-2	CD	8202 ± 514	509 ± 76	-	113 ± 44	-	13 ± 3	14 ± 3
					WD	9073 ± 475	859 ± 99	-	331 ± 94	-	15 ± 6	19 ± 7
					MID	8436 ± 703	552 ± 113	-	126± 48	-	14 ± 5	11 ± 4
					FWD	9056 ± 460	830 ± 191	-	323 ± 111	-	17 ± 6	25 ± 9
Ramos et al. (2019) [79]	INT			HSR: 15.6–20 SPR: >20 ACC: >1 DEC: <-1	CD	10003 ± 954	590 ± 104	-	199 ± 91	-	218 ± 22	161 ± 19
					WD	10238 ± 665	840 ± 137	-	379 ± 119	-	214 ± 35	182 ± 23
					MID	10377 ± 981	811 ± 207	-	299 ± 142	-	214 ± 17	178 ± 19
					FWD	9825 ± 894	783 ± 251	-	352 ± 125	-	210 ± 29	176 ± 27
	INT U20				CD	8202 ± 514	509 ± 76	-	113 ± 44	-	172 ± 10	108 ± 14
					WD	9073 ± 475	859 ± 99	-	331 ± 94	-	197 ± 19	138 ± 21
					MID	8486 ± 703	553 ± 113	-	126 ± 48	-	172 ± 19	111 ± 17
					FWD	9056 ± 460	830 ± 191	-	323 ± 111	-	193 ± 30	146 ± 25
	INT U17				CD	7899 ± 888	348 ± 61	-	129 ± 85	-	165 ± 22	86 ± 15
					WD	8575 ± 996	637 ± 226	-	283 ± 143	-	199 ± 32	122 ± 16
					MID	8546 ± 1260	434 ± 117	-	96 ± 46	-	150 ± 17	93 ± 14
					FWD	8062 ± 1407	520 ± 243	-	248 ± 143	-	168 ± 35	106 ± 27
Romero-Moraleda et al. (2021) [80]	DOM D1			HSR: >15 ACC: >1 & <1 DEC: <-1 & >-1	All	9040 ± 938	1108 ± 294	-	-	-	255 ± 40	78 ± 16
Sausaman et al. (2019) [81]	COL D1			HSR: >15 SPR: >18	All	9486 ± 300	1014 ± 118	-	428 ± 70	-	-	-
					DEF	9039 (8527–9551)	868 (665–1071)	-	385 (265–504)	-	-	-
					MID	9536 (8998–10034)	840 (626–1054)	-	267 (141–393)	-	-	-
					ATT	9882 (9414–10349)	1333 (1147–1519)	-	633 (524–743)	-	-	-
Scott et al. (2020) [8]	DOM D1 (INT)			HSR: >12.5 VHSR: >19 SPR: >22.5	GK	4743 (4370–4742)	222 (0–480)	17 (0–111)	3 (0–40)	-	-	-
					CD	9398 (9110–9686)	1969 (1770–2168)	350 (277–422)	98 (70–127)	29.6 (28.8–30.3)	-	-
					WD	9892 (9637–10147)	2520 (2292–2696)	589 (528–651)	192 (166–218)	30.1 (29.5–30.6)	-	-
					CAM	10644 (10456–10931)	2749 (2551–2947)	487 (415–559)	129 (45–119)	28.7 (28.0–29.5)	-	-
					CDM	10228 (9860–10596)	2264 (2011–2518)	384 (292–477)	82 (45–119)	29.4 (28.3–30.5)	-	-
					WM	10375 (9942–10808)	2659 (2361–2958)	666 (559–773)	248 (204–291)	30.6 (29.5–31.6)	-	-
					FWD	9738 (9500–9976)	2312 (2147–2476)	564 (506–622)	209 (185–232)	30.3 (29.8–30.8)	-	-
	DOM D1 (non-INT)				GK	4445 (4148–4742)	181 (0–385)	11 (0–85)	1 (0–31)	25.8 (25.0–26.6)	-	-
					CD	9408 (9203–9613)	1936 (1795–2078)	382 (331–433)	96 (75–116)	29.7 (29.1–30.2)	-	-
					WD	10076 (9876–10276)	2430 (2292–2568)	512 (463–561)	154 (134–174)	29.8 (29.3–30.3)	-	-
					CAM	10619 (10333–10905)	2648 (2451–2846)	375 (304–446)	59 (26–91)	29.2 (28.5–29.9)	-	-
					CDM	10244 (9924–10566)	2345 (2124–2567)	316 (236–396)	59 (26–91)	28.9 (28.1–29.7)	-	-
					WM	10338 (10060–10616)	2651 (2459–2843)	541 (472–610)	152 (124–180)	29.9 (29.1–30.7)	-	-
					FWD	9867 (9679–10056)	2423 (2292–2553)	585 (539–631)	187 (168–206)	30.1 (29.6–30.5)	-	-
Scott et al. (2020) [82]	DOM D1			HSR: >12.5 VHSR: >19 SPR: >22.5	All	10068 ± 615	2401 ± 454	398 ± 143	122 ± 69	-	-	-
Trewin et al. (2018) [20]	INT			HSR: >16.48 SPR: >19.98 ACC: >2.26	All	10368 ± 952	930 ± 348	-	-	-	174 ± 33	-
					CB	9533 ± 650	661 ± 221	-	-	-	187 ± 33	-
					FB	10496 ± 822	1191 ± 314	-	-	-	185 ± 27	-
					MID	10962 ± 750	973 ± 334	-	-	-	158 ± 33	-
					FWD	10380 ± 893	1037 ± 305	-	-	-	174 ± 27	-
Vescovi (2012) [86]	DOM D1			SPR: >18	All	-	-	-	-	21.8 ± 2.3	-	-
					DEF	-	-	-	-	21.9 ± 2.1	-	-
					MID	-	-	-	-	21.4 ± 2.1	-	-
					FWD	-	-	-	-	22.1 ± 2.4	-	-
Vescovi (2014) [40]	DOM U17			HSR: 15.6–20 SPR: >20	All	8558 ± 864	658 ± 209	-	235 ± 128	25.6 ± 1.9	-	-
	DOM U16				All	8024 ± 802	611 ± 198	-	185 ± 119	25.6 ± 1.6	-	-
	DOM U15				All	6961 ± 789	458 ± 192	-	76 ± 116	24.3 ± 1.7	-	-
	DOM U15-U17				DEF	7779 ± 853	590 ± 201	-	188 ± 120	25.6 ± 1.5	-	-
					MID	8449 ± 850	600 ± 200	-	131 ± 120	24.7 ± 2.0	-	-
					FWD	7952 ± 846	665 ± 201	-	275 ± 119	26.7 ± 1.7	-	-
Wells et al. (2015) [89]	COL D1	Regular season		HSR 15.96–21.9 SPR >22	All	7482 ± 959	557 ± 137	-	86 ± 80	23.3 ± 1.9	-	-
		Post-season			All	8201 ± 693	604 ± 139	-	85 ± 81	23.7 ± 2.4	-	-
Williams et al. (2019) [42]	COL D1			NS	All	9541 ± 178	-	-	-	-	-	-

Data presented as mean ± SD or mean (90% CI).

*Data presented as mean ± SE.

** mean calculated from available data.

TD = total distance; HSR = high-speed running; SPR = sprinting; Vmax = maximum velocity; ACC = accelerations; DEC = decelerations. Qualitative VT = HSR “striding; movement is similar to jogging but involves a longer stride and more pronounced arm swing”; SPR “maximal effort with a greater extension of the lower leg during forward swing and higher heel lift relative to striding”. NS = not specified. Sample/Group: COL = college; DOM = domestic; INT = international; U = Under; D = division, UEFA CL = UEFA Champions League. Playing Position: GK = goalkeeper; DEF = defender; CB = centre back; CD = central defender; FB = full-back; MID = midfield; CM = central midfield; WM = wide midfield; FP = flank player; ATT = attacker; CATT = central attacker; WATT = wide attacker; FWD = forward.

Whole-match relative physical characteristics are presented in Table 7. In addition to those presented, Ramos et al. [33] also quantified relative repeated acceleration and SPR actions per playing position (0.12–0.15 n∙min^-1^). Only 14 studies quantified whole-match physical characteristics relative to match-duration [19, 20, 33, 36, 40, 46, 49, 50, 63, 71, 72, 80, 84, 89]. The majority of these studies reported relative values to explore the impact of contextual factors on physical characteristics [36, 46, 49, 50, 63, 71, 84, 89].

**Table 7:** Studies quantifying physical characteristics of women’s soccer match-play per whole-match as relative data.

Study	Sample / Group		Velocity (km∙h^-1^) and Acceleration (m∙s^-2^) Thresholds	Playing Position	TD (m∙min^-1^)	HSR (m∙min^-1^)	VHSR (m∙min^-1^)	SPR (m∙min^-1^)	ACC (n∙min^-1^)	DEC (n∙min^-1^)
Benjamin et al. (2020) [36]	COL D1	Low WBGT	N/A	All	145 ± 13	**-**	**-**	**-**	**-**	**-**
		Moderate WBGT		All	134 ± 13	**-**	**-**	**-**	**-**	**-**
		High WBGT		All	138 ± 13	**-**	**-**	**-**	**-**	**-**
Bohner et al. (2015) [49]	COL D1	Sea-level	HSR: >15	All	120 ± 9	27 ± 10	**-**	**-**	**-**	**-**
		Altitude		All	106 ± 10	25 ± 8	**-**	**-**	**-**	**-**
Bozzini et al. (2020) [50]	COL D1	In-conference	HSR: 15–19.9 SPR: >20	All	103 ± 8.7	10.0 ± 2.1	**-**	3.1 ± 1.8	**-**	**-**
		Out-conference		All	105 ± 9.1	10.3 ± 2.8	**-**	3.2 ± 2.1	**-**	**-**
Harkness-Armstrong et al. (2021) [19]	U16 DOM D1		HSR: >12.5 VHSR: >19 SPR: >22.5	All	93 ± 24	20.5 ± 11.4	3.0 ± 1.4	0.6 ± 1.4	**-**	**-**
				CD	84 ± 15	15.8 ± 7.1	2.5 ± 1.9	0.5 ± 0.6	**-**	**-**
				WD	92 ± 15	20.8 ± 7.7	3.3 ± 1.9	0.7 ± 0.6	**-**	**-**
				CM	101 ± 16.7	20.3 ± 8.0	1.5 ± 2.2	0.2 ± 0.7	**-**	**-**
				WM	96 ± 15	24.4 ± 7.8	3.9 ± 1.9	0.9 ± 0.6	**-**	**-**
				FWD	91 ± 12	21.0 ± 6.6	3.8 ± 1.5	0.9 ± 0.5	**-**	**-**
	U14 DOM D1			All	92 ± 26	19.8 ± 12.1	2.4 ± 1.5	0.4 ± 1.5	**-**	**-**
				CD	85 ± 15	16.1 ± 7.6	2.5 ± 1.9	0.4 ± 0.6	**-**	**-**
				WD	89 ± 15	19.0 ± 7.7	2.4 ± 2.1	0.3 ± 0.7	**-**	**-**
				CM	101 ± 17	20.8 ± 8.6	1.5 ± 2.3	0.2 ± 0.8	**-**	**-**
				WM	97 ± 15	22.7 ± 7.7	2.7 ± 1.9	0.4 ± 0.6	**-**	**-**
				FWD	89 ± 14	20.2 ± 7.2	3.2 ± 1.8	0.5 ± 0.6	**-**	**-**
Julian et al. (2020) [63]	DOM D1 & D3	Follicular phase	HSR: 13.2 ± 0.7–16.69 ± 1.1 VHSR: 16.69 ± 1.1–19.94 ± 0.9 SPR: >19.9 ± 0.9	All	103 ± 7.7	11.4 ± 3.4	5.9 ± 2.2	3.7 ± 2.4	**-**	**-**
		Luteal phase		All	104 ± 6.8	11.6 ± 3.3	6.6 ± 2.7	4.0 ± 2.0	**-**	**-**
McCormack et al. (2015) [71]	COL D1	Previous match >42h	HSR: 12.99–21.99 SPR: >21.99	All	120 ± 8	25.4 ± 7.2	-	-	**-**	**-**
		Previous match <42h		All	116 ± 8	22.9 ± 5.7	-	-	**-**	**-**
Meylan et al. (2017) [72]	INT		HSR: 16.5–19.9 SPR: >20 ACC: >2.26	All	107 ± 16	6.0 ± 2.1	-	2.9 ± 1.2	1.78 ± 0.67	-
Ramos et al. (2019) [33]	INT		HSR: 12.1–15.5 VHSR: 15.6–20 SPR: >20 ACC: >1 DEC: <-1	CD	109 ± 5.2*	13.7 ± 1.0*	6.4 ± 0.8*	2.2 ± 0.6*	0.06 ± 0.02*	0.08 ± 0.02*
				FB	110 ± 5.4*	13.1 ± 1.1*	8.7 ± 0.8*	4.4 ± 0.6*	0.06 ± 0.03*	0.14 ± 0.02*
				CM	110 ± 5.4*	14.5 ± 1.1*	7.4 ± 0.8*	2.7 ± 0.6*	0.04 ± 0.03*	0.09 ± 0.02*
				WM	109 ± 5.5*	15.0 ± 1.1*	8.6 ± 0.8*	4.2 ± 0.6*	0.04 ± 0.03*	0.14 ± 0.02*
				ATT	101 ± 5.2*	12.8 ± 1.0*	7.7 ± 0.8*	3.4 ± 0.6*	0.06 ± 0.02*	0.16 ± 0.02*
Romero-Moraleda et al. (2021) [80]	DOM D1		HSR: >15 ACC: >1 & <1 DEC: <-1 & >-1	All	95 ± 9	12.1 ± 2.4	-	-	-	-
				CB	86	10	-	-	0.66	0.23
				WB	94	14	-	-	0.70	0.25
				CM	104	11	-	-	0.58	0.18
				WM	92	12	-	-	0.71	0.24
				ATT	99	15	-	-	0.86	0.30
Trewin et al. (2018) [20]	INT		HSR >16.48 SPR >19.98 ACC: >2.26	All	108 ± 10	9.7 ± 3.7	-	-	1.82 ± 0.35	-
				CB	100 ± 7.3	6.9 ± 2.3	-	-	1.96 ± 0.35	-
				FB	110 ± 9.2	12.5 ± 3.3	-	-	1.95 ± 0.29	-
				MID	115 ± 7.9	10.2 ± 3.5	-	-	1.65 ± 0.34	-
				FWD	108 ± 10	10.8 ± 3.2	-	-	1.81 ± 0.28	-
Trewin et al. (2018) [84]	INT	Sea-level (≤500m)	HSR: >16.48 SPR: >19.98 ACC: >2.26	All	108 ± 9.8	9.8 3.3	-	-	1.80 ± 3.8	-
		Altitude (>500m)		All	104 ± 7.8	9.3 ± 2.9	-	-	1.85 ± 0.40	-
		Cold/mild (<21°C)		All	108 ± 9.5	9.8 ± 3.4	-	-	1.84 ± 0.35	-
		Warm/hot (≥21°C)		All	106 ± 9.9	9.5 ± 2.9	-	-	1.73 ± 0.44	-
		Win		All	108 ± 9.7	9.5 ± 3.4	-	-	1.77 ± 0.36	-
		Draw		All	104 ± 9.6	9.2 ± 3.4	-	-	1.91 ± 0.45	-
		Loss		All	107 ± 9.4	10.3 ± 2.9	-	-	1.83 ± 0.38	-
		Win vs higher ranked OPP		All	111 ± 9.0	9.9 ± 3.1	-	-	1.81 ± 0.27	-
		Draw vs higher ranked OPP		All	104 ± 9.9	8.2 ± 3.3	-	-	1.82 ± 0.47	-
		Loss vs higher ranked OPP		All	107 ± 10	10.1 ± 2.8	-	-	1.84 ± 0.39	-
		Win vs lower ranked OPP		All	108 ± 9.7	9.4 ± 3.4	-	-	1.76 ± 0.37	-
		Draw vs lower ranked OPP		All	105 ± 9.0	11.1 ± 2.8	-	-	2.07 ± 0.35	-
		Loss vs lower ranked OPP		All	107 ± 7.7	10.9 ± 3.0	-	-	1.80 ± 0.33	-
		Previous match >72h		All	108 ± 9.5	9.7 ± 3.0	-	-	1.79 ± 0.36	-
		Previous match <72h		All	107 ± 9.7	10.0 ± 3.4	-	-	1.85 ± 0.39	-
Vescovi & Falenchuk (2019) [46]	DOM	Home	HSR: 16.1–20 SPR: 20–32	All	112**	8.4 ± 0.4*	-	4.0 ± 0.4*	-	-
		Away		All	110**	8.1 ± 0.4*	-	3.8 ± 0.3*	-	-
		Natural Turf		All	108**	7.3 ± 0.4*	-	3.8 ± 0.4*	-	-
		Artificial Turf		All	112**	8.6 ± 0.4*	-	3.9 ± 0.4*	-	-
		Win		All	111**	8.3 ± 0.5*	-	3.9 ± 0.5*	-	-
		Draw		All	112**	8.5 ± 0.5*	-	4.3 ± 0.4*	-	-
		Loss		All	111**	7.9 ± 0.5*	-	3.4 ± 0.3*	-	-
Vescovi (2014) [40]	DOM U17		N/A	All	100 ± 12	-	-	-	-	-
	DOM U16			All	100 ± 8	-	-	-	-	-
	DOM U15			All	86 ± 10	-	-	-	-	-
	DOM U15-U17			DEF	97 ± 15	-	-	-	-	-
				MID	105 ± 10	-	-	-	-	-
				FWD	99 ± 11	-	-	-	-	-
Wells et al. (2015) [89]	COL D2	Regular Season	HSR: 15.96–21.9 SPR: >22	All	105 ± 13	7.9 ± 2.5	-	1.2 ± 1.2	-	-
		Post-season		All	98 ± 13	7.1 ± 1.7	-	1.0 ± 0.9	-	-

Data presented as mean ± SD. TD = total distance; HSR = high-speed running; VHSR = very-high speed running; SPR = sprinting; ACC = accelerations; DEC = decelerations. NS = not specified. Sample/Group: COL = college; DOM = domestic; INT = international; U = Under; D = division; OPP = opponent; WBGT = wet bulb-globe temperature. Playing Position: DEF = defender; CB = centre back; CD = central defender; FB = full-back; MID = midfield; CM = central midfield; WM = wide midfield; ATT = attacker; FWD = forward.

*Data presented as mean ± SE.

** mean calculated from available data

#### 3.4.2 Half-match physical characteristics

Eighteen studies reported half-match physical characteristics [26, 31, 32, 34, 38, 40, 41, 43, 44, 49, 50, 54, 69, 70, 73, 74, 77, 78, 86, 89], with the reported data presented in S4 Table. In addition to the data presented, Mara et al. [69] identified players performed a total of 226 and 221 decelerations during the first and second half of elite senior match-play, respectively. Furthermore, Mara et al. [69] analysed six different accelerations and decelerations by intensity, categorising accelerations/decelerations dependent upon starting and final velocity. Only six studies reported half-match data according to playing position [26, 40, 41, 74, 77, 86], whilst the remaining studies presented sample or group averages.

#### 3.4.3 Segmental physical characteristics

Fifteen studies quantified physical characteristics by 15-minute segmental time-periods (i.e. 0–15, 15–30 minutes etc.) [31, 35, 38, 42 – 45, 51, 54, 65, 69, 70, 74, 78], however, four studies selectively reported 15-minute time-periods [35, 43, 65, 74]. For example, Panduro et al. [74] presented only the initial and final 15-minute time-periods. Additionally, four studies reported physical characteristics as a mean of all 5-minute segmental periods within match-play [26, 43, 51, 78]. The data presented by segmental time-periods are presented in S5 Table.

#### 3.4.4 Peak physical characteristics

Eight studies quantified the peak physical characteristics of women’s soccer match-play [19, 20, 26, 43, 44, 51, 74, 78]. The reported peak data are presented in Table 8. Panduro et al. [74] also quantified mean heart rate, and observed values between 181 and 183 beats per minute (BPM) dependent upon playing position. All studies quantified peak data in 5-minute durations except for Harkness-Armstrong et al. [19] who quantified 1-10-minute durations for TD, HSR and VHSR. Only two studies [19, 20] adopted a moving average analysis to determine peak durations, whilst the remaining studies adopted a pre-determined segmental analysis approach (i.e. 0–5 minutes, 5–10 minutes etc.).

**Table 8:** Peak physical characteristics of women’s soccer match-play.

Study	Sample/ Group	Velocity (km∙h^-1^) and Acceleration (m∙s^-2^) Thresholds	Absolute/Relative	Playing Position	TD		HSR		VHSR	SPR		ACC		DEC	
					Peak 5-min	Next 5-min	Peak 5-min	Next 5-min	Peak 5-min	Peak 5-min	Next 5-min	Peak 5-min	Next 5-min	Peak 5-min	Next 5-min
Andersson et al. (2010) [43]	INT	HSR: >15 SPR: >25	Absolute (m)	All	-	-	151 ± 7*	79 ± 11*	-	43 ± 3*	13 ± 3*	-	-	-	-
	DOM D1			All	-	-	134 ± 6*	67 ± 8*	-	35 ± 3*	13 ± 3*	-	-	-	-
Bradley et al. (2014) [26]	DOM D1	HSR: >15	Absolute (m)	All	-	-	149 ± 6*	83 ± 5*	-	-	-	-	-	-	-
Datson et al. (2017) [51]	INT	HSR: >14.4	Absolute (m)	All	-	-	223 ± 47	135 ± 47	-	-	-	-	-	-	-
Harkness-Armstrong et al. (2021) [19]	U16 DOM D1	HSR: >12.5 VHSR: >19	Relative (m∙min^-1^)	All	122 ± 23	-	41 ± 16	-	12 ± 6	-	-	-	-	-	-
				CD	112 ± 15	-	34 ± 12	-	11 ± 5	-	-	-	-	-	-
				WD	120 ± 14	-	41 ± 11	-	13 ± 5	-	-	-	-	-	-
				CM	127 ± 16	-	40 ± 13	-	8 ± 6	-	-	-	-	-	-
				WM	127 ± 15	-	48 ± 11	-	14 ± 5	-	-	-	-	-	-
				FWD	121 ± 13	-	41 ± 10	-	14 ± 5	-	-	-	-	-	-
	U14 DOM D1			All	120 ± 25	-	40 ± 17	-	10 ± 6	-	-	-	-	-	-
				CD	112 ± 15	-	34 ± 12	-	10 ± 5	-	-	-	-	-	-
				WD	118 ± 16	-	40 ± 12	-	10 ± 6	-	-	-	-	-	-
				CM	126 ± 17	-	42 ± 13	-	8 ± 6	-	-	-	-	-	-
				WM	125 ± 15	-	45 ± 12	-	14 ± 5	**-**	-	-	-	-	-
				FWD	120 ± 15	-	41 ± 12	-	14 ± 5	-	-	-	-	-	-
Mohr et al. (2008) [44]	Top-Class	HSR: >15	Absolute (m)	All	-	-	183 ± 9*	77 ± 6*	-	-	-	-	-	-	-
	High-Level			All	-	-	138 ± 8*	88 ± 10*	-	-	-	-	-	-	-
Panduro et al. (2021) [74]	DOM D1	HSR: >15 VHSR: >18 SPR: >25 ACC: >3 DEC: <-3	Absolute (m)	CD	625 ± 27	-	132 ± 36	-	74 ± 20	12 ± 9	-	2.4 ± 1.2	-	2.7 ± 0.8	-
				FB	664 ± 47	-	169 ± 37	-	101 ± 28	21 ± 14	-	2.3 ± 1.0	-	3.2 ± 0.9	-
				CM	683 ± 57	-	165 ± 42	-	91 ± 27	19 ± 14	-	2.3 ± 1.1	-	3.3 ± 1.0	-
				EM	658 ± 52	-	177 ± 37	-	110 ± 24	29 ± 20	-	1.9 ± 1.6	-	3.7 ± 1.0	-
				FWD	639 ± 74	-	167 ± 32	-	104 ± 28	24 ± 18	-	2.6 ± 1.4	-	3.6 ± 1.0	-
Ramos et al. (2017) [78]	INT U20	HSR: 15.6–20 SPR: >20 ACC: >2 DEC: <-2	Absolute (m)	CD	601 ± 57	459 ± 126	69 ± 16	27 ± 19	-	37 ± 15	6 ± 8	2.1 ± 0.6	1.1 ± 0.8	2.6 ± 0.5	0.7 ± 0.7
				WD	653 ± 41	530 ± 66	100 ± 16	47 ± 25	-	57 ± 17	21 ± 19	3.0 ± 0.9	0.7 ± 0.5	3.4 ± 0.8	0.9 ± 0.5
				MID	594 ± 51	470 ± 83	71 ± 17	33 ± 17	-	36 ± 14	5 ± 8	2.4 ± 0.8	1.0 ± 0.7	2.2 ± 0.6	1.1 ± 0.9
				FWD	623 ± 58	504 ± 82	92 ± 28	48 ± 23	-	61 ± 15	18 ± 15	3.4 ± 1.1	1.2 ± 0.8	3.9 ± 1.1	0.9 ± 0.8
Trewin et al. (2018) [20]	INT	HSR: >16.48 ACC: >2.26	Absolute (m)	All	704 ± 59	540 ± 84	123 ± 41	38 ± 24	-	-	-	17 ± 3	10 ± 3		-
				CB	658 ± 49	498 ± 64	101 ± 45	24 ± 19	-	-	-	17 ± 3	11 ± 3		-
				FB	718 ± 46	551 ± 88	153 ± 39	48 ± 25	-	-	-	18 ± 3	11 ± 2		-
				MID	732 ± 50	512 ± 21	126 ± 34	43 ± 25	-	-	-	15 ± 3	9 ± 3		-
				FWD	707 ± 61	543 ± 82	127 ± 31	44 ± 22	-	-	-	17 ± 4	10 ± 3		-
			Relative (m∙min^-1^)	All	141 ± 12	108 ± 16	25 ± 8	7.7 ± 4.9	-	-	-	3.3 ± 0.6	2.0 ± 0.6		-
				CB	132 ± 9.8	100 ± 13	20 ± 9	4.8 ± 3.7	-	-	-	3.4 ± 0.6	2.2 ± 0.6		-
				FB	144 ± 9.1	110 ± 18	31 ± 8	9.7 ± 4.9	-	-	-	3.6 ± 0.5	2.2 ± 0.4		-
				MID	146 ± 9.9	113 ± 17	25 ± 7	8.5 ± 5.0	-	-	-	3.0 ± 0.5	1.8 ± 0.5		-
				FWD	141 ± 12	109 ± 16	25 ± 6	8.7 ± 4.4	-	-	-	3.4 ± 0.7	2.0 ± 0.6		-

NS = not specified. TD = total distance; HSR = high-speed running; SPR = sprinting; Vmax = maximum velocity; ACC = accelerations; DEC = decelerations. Group/Sample: INT = international; DOM = domestic, D = Division, CL = Champions League, U = under. Playing Position: CB = centre back; CD = central defender; WD = wide defender; FB = full-back; MID = midfield; FWD = forward.

*Data presented as mean ± SE.

### 3.5 Technical characteristics

Of the twenty-six studies (38%) which included technical characteristics [25–28, 31, 34, 37, 44, 47, 48, 50, 53, 55, 58–60, 64, 65, 67, 68, 75, 76, 83, 85, 87, 88], six studies quantified technical characteristics in addition to the quantification of physical characteristics [26, 31, 34, 44, 50, 65], two studies predicted the impact of technical characteristics [53] and situational variables [60] on match outcome, and three studies further analysed heading [58, 75] or tackling actions [85] to explore frequency, characteristics, and incidence of associated injuries to understand potential areas of risk. Whilst four studies [28, 64, 68, 87] explored the technical-tactical characteristics of shooting and the respective play prior to shots by elite senior players.

The whole-match technical characteristics reported as player or team averages in these studies are presented in Table 9. In addition to the data presented, studies also presented characteristics as season or tournament totals [27, 31, 48, 59, 64, 67, 75, 83, 85, 87, 88] or position-specific characteristics (defenders vs midfielders vs forwards [44, 55, 58, 75, 87, 88] or central defenders vs wide defenders vs central midfielders vs wide midfielders vs forwards [37]), selected technical characteristics by pitch location (e.g. ball possession, touches, passes, ball recoveries, headers, shots [25, 27, 28, 55, 58, 64, 67, 68, 76, 87, 88], technical-tactical offensive characteristics [27, 28, 64, 68, 87], team set-piece characteristics [27, 31, 37, 47, 48, 67] and reported yellow or red cards [27, 55, 67]. Technical characteristics were predominantly quantified as whole-match characteristics, however three and two studies also presented results by halves [26, 44, 87] and 15-minute segmental periods [85, 87], respectively. Eleven studies included contextual factors within analysis; playing standard [34, 44], age-group [37, 58, 75], match outcome [60, 67, 83], team or opposition ranking [60, 68, 76], match location [60], competition type [50], and playing surface [55,60]. Lastly, only 9 studies [27, 37, 50, 53, 67, 68, 75, 76, 85] either included or provided a reference for the definition of technical characteristics.

**Table 9:** Whole-match technical characteristics of women’s soccer match-play, presented as player or team averages.

Study	Data Collection	Sample/ Group	Technical Variable	Player Average	
Andersen et al. (2016) [31]	Video camera; InStat	DOM D1-D3	Shots	1.4 ± 1.8	
			Shots on target	0.8 ± 1.3	
			Total passes (successful)	44 ± 13 (68 ± 11%)	
			Short passes (0-10m; successful)	11 ± 4 (70 ± 18%)	
			Medium passes (10-40m; successful)	32 ± 12 (68 ± 12%)	
			Long passes (>40m; successful)	1 ± 1 (38 ± 42%)	
			Crosses	20	
			Tackles	6 ± 3	
			Headers	4 ± 3	
			Interceptions	8 ± 5	
Bradley et al. (2014) [26]	25 Hz multi-camera match analysis system (Amisco Pro)	DOM D1	Touches per possession	2.1 ± 0.1*	
			Time in possession (s)	67 ± 3*	
			Total balls lost	17 ± 1*	
			Successful passes (%)	72 ± 2*	
			Duels Won (%)	51 ± 2*	
Krustrup et al. (2005) [65]	Video camera	DOM D1	Headers	8 (3–19)	
			Tackles	14 (7–21)	
				**Team Average**	
Althoff et al. (2010) [25]	Video camera	INT	Ball control	225	
			Short pass (<25 m)	243	
			Long pass (>25 m)	57	
			Shots	201	
			Goals	3	
			Dribbles	44	
			Tackles	8	
Casal et al. (2021) [27]	Video camera; InStat	DOM D1	Shots	1	
			Shots on target	11	
			Goals	4	
			Crosses (successful)	11 (3)	
			Dribbles (successful)	33 (17)	
			Passes (successful)	393 (276)	
			Tackles (successful)	44 (26)	
			Aerial challenge (successful)	43 (22)	
			Interceptions	64	
			Fouls	12	
			Recovered balls	68	
Gómez et al. (2008) [28]	Video camera; Infofutbol	INT	Shots	15 ± 8	
			Shot on target	9	
			Goals	2 ± 2	
Hjelm (2011) [59]	Video camera	INT	Ball actions	614	
Ibáñez et al. (2018) [60]	NS	DOM D1	Goals	2 ± 2	
Konstadinidou & Tsigilis (2005) [64]	Video camera	INT	Shots from combination (%)	23–37	
			Shots from individual attempt (%)	12–20	
			Shots from cross (%)	11–27	
			Shots from set-play (%)	19–31	
			Shots from opponent error (%)	10–21	
Tscholl et al. (2007) [85]	NS	INT	Tackles	147 ± 5 (139–158)	
Wang & Qin (2020) [87]	NS	INT	Shots on target (% shots)	35	
			Rate of goal scoring (% shots)	11	
Wang & Qin (2020) [88]	Video camera	INT	Passes (successful)	347–410 (72–74%)	
			Through passes (successful)	127–142 (58–63%)	
			Dribble successful (%)	48–59	
			Shots	10–15	
			Shots on target	2–7	
			Goals	1–2	
**Playing Standard**				**INT**	**DOM D1**
Gabbett et al. (2008) [34]	Video camera	INT & DOM	Dribbling contacts	14 ± 6	17 ± 5
			Passing contacts	29 ± 9	28 ± 8
			Trapping contacts	24 ± 8	22 ± 6
			Tackling contacts	10 ± 5	10 ± 5
			Other contacts	11 ± 5	19 ± 16
Mohr et al. (2008) [44]	Video camera	INT & DOM		**Top-Class**	**High-Level**
			Headers	11 ± 1*	8 ± 1*
			Tackles	16 ± 1*	14 ± 1*
**Age-Group**				**U16**	**U14**
Harkness-Armstrong et al. (2020) [37]	Video camera; Nacsport Pro+	DOM	Number of Possessions	35 ± 33	32 ± 36
			Total possession (s)	45 ± 82	39 ± 96
			Average possession (s)	1.2 ± 1.0	1.1 ± 1.2
			Touches per possession	2 ± 1	2 ± 1
			Offensive touch	74 ± 99	68 ± 102
			Pass (successful)	25 ± 28 (65 ± 30%)	22 ± 28 (63 ± 32%)
			First touch pass (successful)	8 ± 4 (64 ± 29%)	7 ± 3 (62 ± 31%)
			Dribble (successful)	4 ± 7 (33 ± 15%)	4 ± 8 (31 ± 16%)
			Cross	0.7 ± 0.0	0.8 ± 0.0
			Shot	1 ± 3	1 ± 3
			Defensive touch	14 ± 12	17 ± 15
			Aerial challenge	3 ± 3	2 ± 3
			Block	1 ± 3	1 ± 3
			Clearance	0.9 ± 4	0.7 ± 3
			Interception	4 ± 4	5 ± 5
			Tackle	3 ± 4	4 ± 5
			Foul	0.5 ± 1	0.5 ± 2
Harris et al. (2019) [58]	Video camera	DOM		**U15**	**U13-U14**
			Headers	0–9	0–9
Peek et al. (2021) [75]	Video camera	DOM		**Team Average**	
				**U15 –U17**	**U13 –U14**
			Headers	30–53 (15–69)	20–25 (13–68)
			Heading Duels (%)	15–23%	5–9%
**Contextual Factors—Type of Competition**				**In-Conference**	**Out-Conference**
Bozzini et al. (2020) [50]	InStat	COL D1	Passing accuracy (%)	74 ± 6	76 ± 5
			Dribble success (%)	46 ± 14	53 ± 23
			Tackle success (%)	65 ± 11	53 ± 15
			Challenges won (%)	58 ± 11	56 ± 9
**Contextual Factors—Team Ranking**				**High**	**Low**
Póvoas et al. (2020) [76]	InStat	INT	Successful tackles	1 ± 1	2 ± 1
			Recoveries	6 ± 4	7 ± 3
			Accurate passes (%)	75 ± 9	77 ± 8
			Challenges (won)	13 ± 3 (55 ± 9%)	20 ± 6 (55 ± 15%)
**Contextual Factors—Type of Playing Surface**				**Grass**	**Turf**
Garcia-Unanue et al. (2020) [55]	OPTA Sports	INT	Goals	0.1 ± 0.2	0.1 ± 0.3
			Shots	1 ± 1	1 ± 1
			Passes (successful)	38 ± 11 (67 ± 9%)	36 ± 13 (69 ±10%)
			Touches**	59	54
			Crosses	1 ± 2	1 ± 2
			Dibbles (successful)	3 ± 3 (62 ± 26%)	2 ± 2 (40 ± 29%)
			Tackles	3 ± 2 (77 ± 20%)	2 ± 1 (85 ± 20%)
			Clearances	2 ± 2	2 ± 2
			Interceptions	2 ± 2	2 ± 2
			Fouls	1 ± 1	1 ± 1
**Contextual Factors—Match Outcome**				**Team Average**	
				**Win**	**Loss**
Kubayi & Larkin (2020) [67]	InStat	INT	Passes (successful)	526 ± 114 (80 ± 5%)	393 ± 108 (74 ± 7%)
			Shots	16 ± 6	8 ± 5
			Shots on target	6 ± 4	3 ± 2
			Dribbles (successful)	39 ± 9 (52 ± 12%)	26 ± 9 (50 ± 13%)
			Aerial challenges (successful)	39 ± 11 (57 ± 10%)	39 ± 11 (43 ± 10%)
			Lost balls	81 ± 12	85 ± 12
			Tackles (successful)	35 ± 10 (63 ± 12%)	40 ± 9 (62 ± 11%)
			Fouls	10 ± 4	10 ± 5
			Ball recoveries	65 ± 10	59 ± 10

NS = not specified. Playing standard: INT = international; DOM = domestic; COL = college.

*Data presented as mean ± SE and/or (range)

**Mean calculated from available data.

### 3.6 Tactical characteristics

Table 10 presents the two studies which explored tactical characteristics of women’s soccer match-play. Both studies [30, 90] quantified playing length and playing width (m), which were defined as the range of all 20 outfield players’ longitudinal positioning, and the range of all 20 outfield players’ lateral positioning, respectively. Zubillaga et al. [90] included additional tactical variables; distance from defender to goal-line (m; distance between least longitudinally advanced opposition defender and opposition goal-line, only when in possession), distance from attacker to goal-line (m; distance between most longitudinally advanced opposition attacker and opposition goal-line, only when in possession), distance between goalkeeper and defender (m; least longitudinally advanced defender), distance between goalkeeper and attacker (m; most longitudinally advanced attacking opponent), and individual playing area (m^2^; derived from dividing playing length and playing width by 20). Only open-play data, collected at 5-second intervals was included within Zubillaga et al. [90], whilst Tenga et al. [30] included ball-in-play data collected at 5 Hz frequency, excluding the initial 5-seconds following set-pieces.

**Table 10:** Studies quantifying tactical characteristics of women’s soccer match-play.

Study	Data Collection	Tactical Variable	Ball Location (zones by pitch length)					
			Zone 1 (defensive third)	Zone 2 (defensive third)	Zone 3 (middle third, defensive half)	Zone 4 (middle third, offensive half)	Zone 5 (offensive third)	Zone 6 (offensive third)
Tenga et al. (2015) [30]	25 Hz multi-camera match analysis system (Amisco Pro) Data utilised 5 Hz sampling	Playing length (m)	43.3 ± 7.6	39.4 ± 5.5	37.4 ± 5.7	36.7 ± 4.1	40.4 ± 4.3	48.1 ± 3.9
		Playing width (m)	39.2 ± 8.1	42.4 ± 8.5	42.9 ± 20.3	41.8 ± 8.0	40.9 ± 7.1	40.4 ± 8.4
Zubillaga et al. (2013) [90]	25 Hz multi-camera match analysis system (Amisco Pro) Data utilised 5-second intervals	Playing length (m)	43.3 ± 7.6	39.4 ± 5.5	37.1 ± 5.3	36.4 ± 4.2	40.2 ± 4.2	48.1 ± 4.0
		Playing width (m)	39.2 ± 8.1	42.7 ± 8.5	43.8 ± 18.4	42.8 ± 17.6	40.5 ± 7.0	39.8 ± 7.7
		Distance from defender to goal-line (m)	45.8 ± 7.6	43.8 ± 6.9	36.9 ± 13.4	28.0 ± 7.5	16.9 ± 6.7	6.9 ± 7.5
		Distance from attacker to goal-line (m)	16.5 ± 11.1	22.0 ± 7.4	31.5 ± 7.4	40.8 ± 7.5	48.6 ± 6.6	52.2 ± 8.0
		Distance between goalkeeper and defender (m)	26.6 ± 4.4	26.4 ± 4.5	24.3 ± 9.9	20.8 ± 9.3	14.5 ± 4.9	7.2 ± 4.7
		Distance between goalkeeper and attacker (m)	12.4 ± 7.4	16.1 ± 5.3	20.8 ± 4.8	25.3 ± 4.5	28.3 ± 4.9	28.7 ± 5.7
		Individual playing area (m^2^)	85.0 ± 23.4	84.4 ± 21.7	81.6 ± 39.2	77.9 ± 32.7	81.3 ± 16.1	96.2 ± 22.6

## 4 Discussion

This is the first systematic review to summarise women’s soccer match-play, including the technical, tactical and physical characteristics. A total of 69 studies were included, predominantly quantifying physical characteristics (68%), whilst 38% quantified technical characteristics, and only 3% quantified tactical characteristics. Studies reported whole-, half-, segmental and/or peak match characteristics for physical data, however studies reporting technical and tactical characteristics were predominantly limited to whole-match analysis. There has been an increase in the number of studies quantifying women’s soccer match-play characteristics in recent years, however, studies predominantly involved senior international (39%) and domestic players (43%), with only 10% quantifying match-play characteristics of youth (age-group) soccer players. Physical characteristics appear to increase between age-groups and playing standards, and are dependent upon playing position. Furthermore, there are between-half decrements in physical performance, with the opening 15-minutes of match-play the most physically demanding. Further research quantifying the technical and tactical characteristics is required to understand differences within and between age-groups and playing standards. The results of this review provide insight into the current match-play characteristics across different playing standards and playing positions, which will be beneficial for researchers and practitioners implementing evidence-based practice with women’s soccer players.

### 4.1 Methodologies for quantifying match-play characteristics

There are important methodological limitations to acknowledge when interpreting and comparing the extracted data. Firstly, over half of the included studies which reported number of teams involved only a single-team or single-club. This is problematic, as results may not be reflective of the population due to individual team/club strategies, tactics or playing styles, which may influence players’ physical, technical and tactical performance. Future research should adopt multi-club data collection approaches to overcome this issue. Secondly, positional categorisation was inconsistent, which may be a consequence of small sample size within studies. Only nine of the twenty-six studies [8, 19, 26, 33, 37, 51, 52, 74, 80] quantifying position-specific characteristics differentiated both central and wide defenders and midfielders. High-level positional categorisations (i.e. defender or midfielder) may not provide accurate insights into match-play characteristics, given the positional differences observed when central and wide players are compared [19, 37, 51, 74]. Thus, in addition to adopting a multi-club approach, future research should ensure sufficient sample size (participants and match observations) to differentiate central and wide playing-positions.

Thirdly, comparing physical characteristics quantified by different equipment requires caution due to between-system differences (i.e., video-based time-motion analyses vs GPS units vs optical tracking; 5 vs 10 vs 15 Hz GPS) [91–94]. Furthermore, where studies have adopted the same data collection method (e.g. 10 Hz GPS units), differences exist between manufacturers’ hardware, firmware, data filtering and data processing methods [95, 96]. Therefore, direct comparison of findings between studies adopting different data collection methods may not be appropriate, and this further limits the potential insights which can be gained regarding the physical characteristics.

Lastly, a range of velocity and acceleration/deceleration thresholds have been adopted, with methods for establishing or adopting thresholds also differing between studies (e.g. arbitrary thresholds within men’s soccer, derived from physical performance characteristics, derived from match-play data of senior women’s soccer players). This has resulted in varying velocity and acceleration/deceleration zones, impacting comparisons between studies. There is a need for future research to establish a standardised approach for adopting velocity and acceleration/deceleration thresholds within women’s soccer to facilitate comparisons between studies. However, practitioners and researchers should also have an awareness and understanding of the potential impact thresholds may have on physical characteristics. For example, recent research found adopting senior-derived velocity thresholds for youth match-play, resulted in an underestimation of higher-speed distances as senior-derived thresholds are too excessive to accurately reflect the physical characteristics of youth players [97]. In this instance, adoption of senior-derived velocity thresholds may lead to misleading data and subsequently erroneous interpretations of the physical characteristics of youth match-play. Therefore, we recommended that researchers and practitioners make an informed-decision, depending upon their study aim or intended use of the data, as to whether it may be more appropriate to adopt senior-derived velocity thresholds or population-specific velocity thresholds.

The underpinning methodological limitations within the body of literature limits the insights which can be gained across women’s soccer populations. Consequently, researchers and practitioners using the match-play characteristics presented within this review, particularly the physical characteristics should be cautious in their interpretation and application of the data. Furthermore, subsequent discussion within this systematic review, is reflective of the limitations highlighted.

### 4.2 Whole-match characteristics

#### 4.2.1 Absolute physical characteristics

The TD covered during match-play by (outfield) women’s soccer players ranged between 5480–11160 m, and appeared to increase between playing standards, with similarities between senior international and domestic match-play. When considering the most common velocity thresholds implemented across respective velocity zones, HSR distance and percentage of TD also increased between playing standards (>15 km∙h^-1^: international = 13.8–17.9%; domestic = 5.9–17.0%; college = 10.1–13.5%) [26, 35, 43, 61, 62, 65, 74, 80, 81]. Whilst, VHSR distance and percentage of TD increased between youth and senior playing standards (>19 km∙h^-1^: domestic = 3.5–6.4%; youth = 1.5–4.2%) [8, 19, 82]. Similar SPR distances were covered by senior international and domestic players when considering the most commonly applied SPR threshold (>20 km∙h^-1^) [31, 40, 57, 73, 78, 79]. Whilst Ramos et al. [78] and Vescovi [40] observed a progressive increase in SPR distances covered by youth players, between U17, U20 and senior international age-groups, and U15, U16 and U17 domestic age-groups, respectively. The progressive increase in distances covered across playing standards and age-groups may be consequential of increasing physical capacities, greater match-specific fitness, reflective of increased technical-tactical demands or potentially differing contextual factors within playing standards. Furthermore, the increase in absolute distances between age-groups may be consequential of differing match-durations between youth and senior match-play [19, 37, 40].

The number of HSR and SPR efforts performed, differed between studies (HSR = 44–376; SPR = 4–70) [20, 29, 31, 40, 43, 44, 52, 63, 65, 69, 71, 73] which is likely due to differing methodological approaches (i.e. data collection, velocity thresholds). However, the mean distance per HSR and SPR efforts was predominantly <10 m [52, 63, 69] and <20 m [31, 40, 43, 52, 63, 65, 86], respectively, which suggests that acceleration ability is important within women’s soccer [12]. Yet, the number of accelerations differed vastly between studies [20, 31, 57, 62, 69, 74, 78, 79, 80], which may also be due to the different methods adopted. For example, the largest discrepancy was observed in studies adopting >2 m∙s^-2^ thresholds (i.e., 20 Hz radio frequency tracking = 161 accelerations [31]; 10 Hz GPS = 13–80 accelerations; [62, 78]; 25 Hz optical tracking = 342–475 accelerations [69]). Future research may aim to quantify acceleration and decelerations into zones or the starting and finishing velocities [69], to further understand the intensities of these movements [57, 62, 64, 77]. In comparison to distances covered in match-play, there is limited understanding of the accelerations and decelerations, across all playing standards. Therefore, future research should aim to further investigate these actions within match-play, particularly considering the associated high metabolic and mechanical loads [57], this information will be useful for preparing players for match-play, or informing player load monitoring, and injury prevention and rehabilitation practices.

#### 4.2.2 Absolute technical characteristics

There were consistent findings in technical characteristics across studies. Unsurprisingly, passes were the most frequent technical action in possession across playing standards, with an increasing pass success rate from youth (63–65%) [37] to senior match-play (67–80%) [26, 27, 31, 50, 55, 67, 76]. Tackles and interceptions were the most common defensive actions in senior and youth match-play [27, 31, 37, 55]. However, given the limited number of technical characteristics quantified across studies, it is difficult to explore potential differences in playing styles across playing standards. Additionally, the technical data presents on-the-ball technical actions for both in-possession (e.g. passing, crosses, or shots) and out-of-possession (e.g. clearances, interceptions, or tackles), which can be useful for practitioners informing coaching practice. However, given the small duration of time spent on the ball (senior = 67 ± 3 s; youth = 32–35 s) [26, 37] and low frequency of these technical actions, future research should aim to quantify off-the-ball technical actions, technical-tactical or physical-technical actions [17], such as pressing, pass effectiveness or sequences/patterns of play [47, 83], to gain better understanding of technical performance.

#### 4.2.3 Absolute tactical characteristics

Tactical characteristics referred to players’ positioning which provide insight to teams’ playing length and width, and players’ individual playing area, dependent upon ball location [30, 90]. The data presented may help practitioners determine appropriate dimensions for training drills, conditioned or small-sided games. However, the reported data lacks physical, technical and situational context. Therefore, further research is required to better understand team behaviours, and how physical and technical characteristics may interact with tactical strategies [27, 28, 68]. Furthermore, tactical characteristics are often dynamic, thus future research should aim to improve our understanding of the tactical characteristics across match-play, or prior-to and following key moments in match-play, and how contextual factors (e.g. match status) may affect tactical performance. However, it is important to acknowledge the challenges and complexities associated with this research and within practice [98, 99]. For instance, collecting sufficient data for appropriate analyses, the advanced theoretical and computational underpinning knowledge required for analyses, ability to work with complex datasets, and multidisciplinary collaboration. We anticipate that as the involvement of big data technologies within the women’s game increases, and more commercial data providers provide readily-available access to physical and technical data [53, 55], the body of literature exploring tactical characteristics in senior international and professional playing standards will grow accordingly.

#### 4.2.4 Position-specific absolute characteristics

Senior goalkeepers cover 4445–5622 m during match-play [8, 62, 74], predominantly at lower speeds. Unsurprisingly, goalkeepers covered less TD, HSR, VHSR and SPR distances, than outfield players. However, given the different movements and technical-tactical skills associated with goalkeeping, position-specific characteristics (e.g. number, intensity, and direction of movements such as diving, jumping or kicking) [100] would be more appropriate than distances covered. Future research should aim to improve our very limited understanding of women’s soccer goalkeeper match-play characteristics, to help inform goalkeeper-specific training and coaching practice.

When studies differentiated central and wide players, central defenders typically covered the lowest TD, HSR, VHSR, and SPR distances [8, 19, 20, 26, 51, 70, 74, 78, 79], performed the least HSR and SPR efforts [20, 52, 73], and had the least total and average duration of possession, touches per possession, and offensive touches during youth match-play [37]. Central midfielders covered the most TD [8, 19, 26, 51, 62, 74], and had the lowest maximum velocity [8, 19]. Furthermore, Harkness-Armstrong et al. [37] reported central midfielders had the greatest active involvement in youth match-play with the most offensive touches and passes, as well as the most tackles. Considering HSR distance, three studies reported wide midfielders covered the most [8, 19, 74], whilst Bradley et al. [26] and Datson et al. [51] reported forwards and central midfielders covered the most, respectively. Forwards and wide midfielders consistently covered the most VHSR and SPR distances [8, 19, 51, 74], and attempted the most dribbles, crosses and shots during youth match-play [37]. There was a discrepancy in position-specific accelerations and decelerations between studies, however this may be due to respective samples adopting notably different acceleration and/or deceleration thresholds. Although comparison of physical and technical characteristics are limited due to methodological differences, clear differences exist between playing positions and the data reported within the current review can be used to inform position-specific training drills and coaching practice to prepare players accordingly for match-play.

#### 4.2.5 Relative characteristics

Between playing standards, college players covered the highest relative TD [36, 49, 50, 71, 89], whilst senior international players covered more relative TD [20, 33, 72, 84] than senior domestic players [46, 63, 80], and youth players covered the least relative TD [19, 40]. Where similar SPR thresholds (>20 km∙h^-1^) were adopted, the data suggests an increase in relative SPR distance between playing standards [33, 46, 50, 72].

Quantifying relative characteristics normalises data to minutes played, removing potential differences in data due to match-duration across match observations, which is particularly useful for comparisons between groups, especially age-groups with differing match durations [19]. However, it is important to acknowledge that relative whole-match data includes ball out of play time, which has been found to be between 38.0 and 41.6% of the time in women’s soccer [37, 51]. Whilst inclusion of ball out of play time has been found to underestimate physical characteristics in men’s soccer populations [101, 102], the effect on women’s soccer players is yet to be quantified. Therefore, future research across women’s soccer populations should explore the differences between ball in play and whole-match characteristics, to better understand the physical and technical characteristics, and to ensure practitioners can implement coaching practice and training drills which better represent match-play demands.

#### 4.2.6 Influence of contextual factors

Excluding playing standard, age-group and playing position, only fourteen studies [36, 38, 46, 49, 50, 55, 63, 67, 68, 71, 76, 83, 84, 89] quantified the influence of contextual factors on match-play characteristics. Studies predominantly reported whole-match characteristics, with physical characteristics primarily presented as relative values. Whereas technical data were either presented as absolute or relative to match duration, which may lead to erroneous interpretation when comparing the effect of contextual factors. For example, Póvoas et al. [76] found international low-ranking teams performed more successful tackles, recoveries and challenges than high-ranking teams. However, this may be due to low-ranking teams having less possession, and thus more opportunity to perform defensive actions than high-ranking teams. Therefore, future research should present possession-dependent technical characteristics relative to possession status [37]. Additionally, studies quantified contextual factors in isolation (i.e. match location, match congestion, or opposition quality), with only Trewin et al. [84] combining contextual factors (i.e. match outcome and opposition quality; win vs higher ranked opponent). Quantifying contextual factors in isolation may be consequential of limited sample size or match observations. However, caution should be taken when interpreting the influence of isolated contextual factors given the complex, multifaceted nature of match-play performances [103].

Acknowledging these limitations; high temperatures [36, 84], altitude [49, 84], match congestion (<42h) [71, 84], playing against lower ranked opposition [38, 84], playing on grass rather than artificial turf [46], in-conference matches as opposed to out-of-conference fixtures (type of competition) [50], and competing in matches post-season compared to regular-season (seasonal changes) [89], resulted in reduced physical characteristics. However, all studies were conducted with a single-team, thus further research with a multi-club approach, larger sample size and greater number of match observations is required to identify whether these findings are generalizable to the wider women’s soccer population. Four studies quantified match characteristics according to match outcome [46, 67, 83, 84]. Differences in physical and technical performances were observed in teams who won, drew or lost across studies, however caution should be taken when interpreting this data. Grouping match observations by match outcome can be an overly-simplistic approach, which does not reflect the fluidity of match status during observations, and may subsequently lead to erroneous categorisation. For example, a team may score within the final minutes of match-play after drawing for the majority of the match, yet be categorised as a win. Therefore, we recommend future research explores the influence of match status (i.e. drawing, winning or losing) as opposed to match outcome on match-play characteristics. Finally, one study [63] attempted to explore sex-specific considerations on match-play performance. The authors compared physical characteristics during match-play between the follicular and luteal phase of the menstrual cycle. However, due to the complexities of data collection (e.g. small sample size due to strict participant inclusion criteria, individual and match-to-match variability) the authors could not attribute changes in performance to the menstrual cycle. Whilst there are considerable complexities to conducting such research [104], further work is warranted to explore the influence of sex-specific considerations on match-play performances.

### 4.3 Half-match characteristics

The twenty-two studies which quantified half-match characteristics [26, 31, 32, 34, 38–41, 43, 44, 49, 50, 54, 69, 70, 73, 74, 77, 78, 86, 87, 89] predominantly reported between-half decrements in performances. Reductions in TD (0.2–29.7%), HSR (0.9–26.6%), VHSR (4.6–12.0%) and SPR (4.6–29.5%) distances were observed between-halves, across all playing standards. Only two instances of increased HSR distance between-halves were observed [26, 40]. Whilst Vescovi & Favero [41] were the only authors to report an increase in SPR distance (college = 3.1–32.2%) between-halves. This inconsistency may be consequential of the authors’ data inclusion of half-match observations as opposed to whole-match, which resulted in differing numbers of player observations which may have involved different player samples in each half, and thus may not be a true reflection of between-half differences in performance. Fewer HSR and sprint efforts were performed during the second-half, whilst, the sprint characteristics (e.g. mean distance per sprint, mean duration per sprint) [40, 69, 73] also reduced between halves, as the interval between sprint efforts increased (10–15%) [70, 73]. Additionally, senior domestic players performed fewer accelerations (5.5–52.1%) and decelerations (2.2–29.5%) during the second-half [69, 74, 77].

Interestingly, alongside the observed reductions in physical performances, senior domestic players had less individual time in possession in the second half (-7.8%; 34.6s vs 31.9s) [26]. The remaining technical characteristics show similar technical performances between-halves. However, only a small number of technical characteristics were quantified, which were predominantly infrequent on-the-ball technical actions (e.g. duels, headers and tackles) [26, 44], therefore limited insight can be gained as to potential differences in technical performances between-halves. Additionally, no position-specific technical characteristics were quantified which is problematic, as player averages may provide limited insight into technical performances, given that technical characteristics differ between positions for whole-match [37, 55, 88]. Furthermore, position-specific differences and between-half decrements were observed in physical half-match characteristics [26, 74, 77]. Therefore, future research across playing standards should aim to quantify half-match physical, technical and tactical characteristics according to playing position. This information will enable practitioners to design and deliver position-specific practices to prepare players for match-play and improve their ability to sustain performances between-halves.

### 4.4 Segmental characteristics

The opening 15-minutes of match-play was consistently the most demanding period across all physical characteristics quantified. Reductions in TD (4.9–26.2%) [35, 38, 44, 70, 74, 78], HSR (15.5–41.0%) [31, 35, 38, 43, 44, 51, 65, 70, 74, 78], VHSR (25.5–35.7%) [74], and SPR (7.8–73%) [31, 38, 43, 44, 70, 74, 78] distances, number of accelerations and decelerations (3.8–66.7%) [74, 78], alongside an increase in mean interval between HSR and SPR efforts (45.5%; 48.5%) [70], were observed across studies from the opening and final 15-minutes of match-play. Only four instances of contrasting data were reported [35, 78], with increased performances observed between the first and last 15-minute period. However, these inconsistencies may also be explained by the studies’ samples. For instance, Bendiksen et al. [35] only observed one team for one match, and Ramos et al. [78] only included 12 players from one team. Consequently, data may be influenced by small sample size and specific team strategies, and therefore may not be representative of the wider populations.

The observed reductions in physical characteristics across match-play (half-match and segmental), may be consequential of reduced physical performance capacities due to physical fatigue, pacing strategies, or an increased perception of effort due to mental fatigue [18, 44, 66]. Furthermore, technical-tactical performance, situational and contextual variables may also contribute to these reductions, however physical characteristics have predominantly been quantified in isolation. Thus, future research should aim to quantify technical and tactical characteristics alongside physical characteristics, as well as exploring the influence of situational and contextual factors, to understand their influence in reductions in physical performance across match-play. For example, playing styles, team strategies or formation may differ over the duration of match-play but also in response to match status [105], substitution strategies influence on observed players’ physical characteristics [106, 107], or whether ball-in play time differs across the match and thus influences physical performances [96, 108]. This information would be important for practitioners when informing technical-tactical drills or conditioned games to prepare players for the fluctuating demands of the game, or training prescription which aims to increase players’ physical capabilities to sustain physical performances throughout match-play, but also for informing tactical coaching interventions during match-play.

### 4.5 Peak characteristics

All eight studies reporting peak characteristics, quantified physical characteristics for a 5-minute period [19, 20, 26, 43, 44, 51, 74, 78]. TD covered appeared to increase with increasing playing standards [19, 20, 74, 78]. However, comparison of HSR, VHSR and SPR distances and number of accelerations and decelerations, during peak-periods is limited, given the different methods adopted across the eight studies (e.g. five HSR velocity thresholds, three accelerations/ decelerations thresholds). Furthermore, two studies [19, 20] quantified peak periods by a moving-averages approach, whilst the remaining studies adopted a pre-determined segmental analysis approach. Previous research within other sporting populations (e.g. men’s soccer, men’s rugby union) found adopting segmental periods can underestimate peak TD and HSR distances by up to 25% in comparison to moving average analysis [109–112]. Therefore, it is likely peak-data quantified via segmental analysis [26, 43, 44, 51, 74, 78] underestimates the peak characteristics of women’s soccer players. Therefore, it is not possible to determine whether differences across playing standards is reflective of the increased demands, or a consequence of different methods. Thus, practitioners utilising peak data to inform coaching practice and training programme design to prepare players for the worst-case scenarios in match-play should be aware of the methods of analysis adopted. Furthermore, future research should adopt a moving average analysis when quantifying peak periods of women’s soccer.

Only one study [19] quantified peak periods at differing durations (1–10 minutes), and observed youth players covered the greatest distances during the 1-minute period, whilst relative distances reduced as duration increased, with the least distances covered during the 10-minute period. This is consistent with previous research in men’s soccer [110, 111]. Whilst underlying reasons why this reduction may occur is not known (e.g. reduction in physiological capacity; differing technical-tactical demands between peak periods) [96], duration-specific peak characteristics can be used to inform duration-specific training programme design or coaching practice. However, it is not appropriate to inform duration-specific training drills when duration-specific data does not align (i.e. 5-minute data to inform 3-minute training drills) [110]. This is problematic, as only 5-minute peak periods have been quantified for senior populations. Therefore, future research should quantify peak characteristics of 1-10-minute durations, to understand the duration-specific worst case scenarios within senior match-play, which can be used to inform duration-specific practices to optimally prepare players for the most physically demanding periods of match-play.

Four studies [19, 20, 74, 78] quantified position-specific peak physical characteristics. During 5-minute peak periods, central defenders typically covered the least TD [19, 20, 74], and HSR distance [19, 20, 74, 78], whilst central defenders and central midfielders covered the least VHSR [19, 74], and SPR [74, 78] distances. Where studies differentiated central and wide defenders and midfielders [19, 74], central midfielders covered the greatest TD, and wide midfielders covered the greatest HSR, VHSR and SPR distances. Furthermore, Harkness-Armstrong et al. [19] reported position-specific differences in TD, HSR and VHSR across 1–10 minute peak durations. The data indicates that peak physical characteristics are position-dependent, thus practitioners should implement position-specific practices to prepare players accordingly for the varying worst case scenarios in match-play. Additionally, the data highlights the need for research to quantify peak characteristics beyond TD (e.g. distances in velocity zones, number of accelerations) to facilitate position-specific differentiation of specific worst-case scenarios.

Consistent with other areas within this review, peak characteristics have quantified physical characteristics in isolation, which provides limited insight into the true demands and context of these worst-case scenarios within match-play [17]. Additionally, recent research in elite men’s soccer [113] found physical peak characteristics lack context due to the multifaceted nature of worst case scenarios, which consequently results in high variability. Therefore, future research should; quantify the associated technical and tactical characteristics during peak physical periods, to understand how technical-tactical roles may influence worst case scenarios; attempt to quantify the peak technical and tactical periods of match-play and the associated physical characteristics; explore how contextual factors (e.g. match status, formation, opposition quality, ball possession) influence worst case scenarios [111, 114], and quantify the variability of peak characteristics in women’s soccer match-play. As previously discussed, attempting to integrate the physical, technical and tactical characteristics, and understand the variation within and between matches will provide greater insight into these worst-case scenarios, and enable evidence-informed design and prescription of coaching practice and training programmes to optimally prepare players for the most demanding periods of match-play.

### 4.6 Limitations

This review has presented study limitations throughout, and the caution required when interpreting results or informing practical applications. For example, this review has identified key methodological limitations within the literature which limits comparisons between studies, including; single-team samples; differing data collection methods; and no standardised velocity and acceleration/deceleration thresholds. Consequently, researchers and practitioners should be cautious in their interpretation of the reported data, whilst future research requires greater consistency in the methods adopted to facilitate comparisons between studies. For example, multi-club samples to ensure findings are generalizable to the population, positional-categorisation of players which differentiate central and wide players as opposed to high-level categorisation (i.e. defenders vs midfielders vs forwards), and to establish and adopt standardised velocity, acceleration and deceleration thresholds/zones for women’s soccer, to facilitate comparisons between and within playing standards.

The heterogeneity of the included studies’ samples and methodologies prevented the inclusion of a meta-analyses within the current systematic review. Given the extent of the current review in summarising all physical, technical, and tactical characteristics during match-play, across all playing standards of women’s soccer, there is a very large breadth of results which may be overwhelming. However, given the recent growth, development, and investment within women’s soccer, the authors strongly believe there is a timely need for the current review; to collate all current evidence regarding women’s soccer match-play characteristics, and provide practitioners with a critical resource which can be utilised to develop evidence-informed practice within women’s soccer populations.

## 5 Conclusions

The quantification and understanding of match-play characteristics is important for informing practices across women’s soccer populations. This is the first systematic review to summarise the scientific literature evaluating the match-play characteristics of women’s soccer, and presents the physical, technical and tactical characteristics of women’s soccer match-play across age-groups, playing standards and playing positions. Furthermore, this review provides a critical evidence-based resource which can be used to inform population-specific practices across women’s soccer playing standards.

The current review has identified that physical characteristics appear to increase between playing standards and differ between playing positions. Furthermore, between-half reductions in physical characteristics were apparent, whilst the opening 15-minutes of match-play was consistently the most physically demanding. Additionally, peak physical characteristics were primarily quantified via a segmental analysis, which may underestimate the true worst-case scenarios of match-play. Therefore, research which quantifies the peak demands for differing durations via a moving-averages method is warranted across women’s soccer playing standards. Additionally, further research is needed to understand technical and tactical characteristics of women’s soccer match-play, and how performances may differ across playing standards. Furthermore, research should aim to integrate physical, technical and tactical characteristics rather than quantifying characteristics in isolation, to gain a holistic understanding of match-performance. In addition, further evidence is required regarding contextual factors within match-play, to understand how the characteristics players face during match-play may vary. Future research may also attempt to better our understanding of the match-to-match variation within women’s soccer populations. As currently only two studies have quantified match-to-match variation of physical characteristics utilising single-team samples, this is therefore not generalizable to the wider population [20, 72]. Finally, there is a heavy bias towards research quantifying match-play characteristics of senior players. The lack of research and subsequent knowledge and understanding of youth match-play characteristics (<U17) is problematic. Thus, further research is necessary within youth populations, to inform long-term talent development, transition of youth players across the talent pathway, and talent identification processes.

## Supporting information

S1 Checklist PRISMA 2020 checklist. (DOCX)

S1 Table Whole-, half-, segmental- and peak-match characteristics of women’s soccer players, quantified via heart rate monitors. (DOCX)

S2 Table Whole-match high-speed running and sprinting match-play characteristics of women’s soccer players. (DOCX)

S3 Table Whole-match acceleration and deceleration characteristics of women’s soccer players. (DOCX)

S4 Table Half-match physical characteristics of women’s soccer match-play. (DOCX)

S5 Table Segmental physical characteristics of women’s soccer-match-play. (DOCX)
