# Supplementary material for: A systematic review of match-play characteristics in women’s soccer
Source: PLoS One. 2022 Jun 30;17(6):e0268334. doi: 10.1371/journal.pone.0268334 (PMC9246157; doi:10.1371/journal.pone.0268334)
Supplement: S1 Table — (DOCX) [file pone.0268334.s002.docx]

**Table S1** Whole-, half-, segmental- and peak-match characteristics of women’s soccer players, quantified via heart rate monitors.

| **Study** | **Sample/ Group** | | **Playing Position** | **Heart Rate Variable** | **Whole** | **Half** | | **Segmental** | | | | | | **Peak** |
| --- | --- | --- | --- | --- | --- | --- | --- | --- | --- | --- | --- | --- | --- | --- |
|  |  |  |  |  |  | **1^st^** | **2^nd^** | **0-15min** | **15-30min** | **30-45min** | **45-60min** | **60-75min** | **75-90min** | **5-min** |
| Andersen et al. (2016) [31] | DOM D1-D3 | | All | Mean HR | (87 ± 5%) | 169 ± 11  (87 ± 5%) | 169 ± 13*  (87 ± 6%) | - | - | - | - | - | - | - |
|  |  |  |  | Peak HR | 190 ± 8 | - | - | - | - | - | - | - | - | - |
| Andersson et al. (2010) [43] | INT | | All | Mean HR | 162 ± 6* (85 ± 3%) | 164 ± 6*  (86 ± 3%) | 162 ± 7*  (85 ± 4%) | 162 ± 7* | 167 ± 9* | - | 158 ± 8* | - | 162 ± 9* | - |
|  |  |  |  | Peak HR | 187 ± 2* (97 ± 3%) | - | - | - | - | - | - | - | - | - |
|  | DOM D1 | | All | Mean HR | 163 ± 5*  (85 ± 3%) | 164 ± 6*  (86 ± 4%) | 159 ± 5*  (84 ± 3%) | - | 163 ± 10* | - | 158 ± 8* | - | - | - |
|  |  |  |  | Peak HR | 185 ± 2* (97 ± 2%) | - | - | - | - | - | - | - | - | - |
| Bozzini et al. (2020) [50] | COL D1 | IC | All | Time (min∙min^-1^) 80-89% HR | - | 0.43 ± 0.09 | 0.49 ± 0.08 | - | - | - | - | - | - | - |
|  |  | OC | All |  | - | 0.35 ± 0.1 | 0.48 ± 0.1 | - | - | - | - | - | - | - |
|  |  | IC | All | Time (min∙min^-1^) 90-100% HR | - | 0.46 ± 0.1 | 0.32 ± 0.1 | - | - | - | - | - | - | - |
|  |  | OC | All |  | - | 0.58 ± 0.1 | 0.38 ± 0.1 | - | - | - | - | - | - | - |
| Jagim et al. (2020) [62] | COL D3 | | All | Mean HR | 142 ± 20  (74 ± 6%) | - | - | - | - | - | - | - | - | - |
|  |  |  | GK | Mean HR | 121 ± 29  (68 ± 8%) | - | - | - | - | - | - | - | - | - |
|  |  |  | CD | Mean HR | 144 ± 23  (74 ± 6%) | - | - | - | - | - | - | - | - | - |
|  |  |  | CM | Mean HR | 147 ± 10  (76 ± 6%) | - | - | - | - | - | - | - | - | - |
|  |  |  | FP | Mean HR | 144 ± 21  (74 ± 5%) | - | - | - | - | - | - | - | - | - |
|  |  |  | FWD | Mean HR | 133 ± 8  (70 ± 4%) | - | - | - | - | - | - | - | - | - |
| Krustrup et al. (2005) [65] | DOM D1 | | All | Mean HR | 167  (87%) | - | - | 166 | 168 | 168 | 163 | 166 | 166 | - |
|  |  |  |  | Peak HR | 186  (97%) | - | - | - | - | - | - | - | - | - |
| Krustrup et al. (2010) [66] | DOM D1 | | All | Mean HR | 168 ± 1  (86 ± 1%) | - | - | - | - | - | - | - | - | - |
|  |  |  |  | Peak HR | 194 ± 2  (98 ± 1%) | - | - | - | - | - | - | - | - | - |
| McFadden et al. (2020) [29] | COL D1 | | All | Time (min) 50-59% HR | 0.9 ± 0.4 | - | - | - | - | - | - | - | - | - |
|  |  |  |  | Time (min) 60-69% HR | 1.9 ± 0.6 | - | - | - | - | - | - | - | - | - |
|  |  |  |  | Time (min) 70-79% HR | 8.0 ± 1.2 | - | - | - | - | - | - | - | - | - |
|  |  |  |  | Time (min) 80-89% HR | 32.0 ± 3.3 | - | - | - | - | - | - | - | - | - |
|  |  |  |  | Time (min) 90-100% HR | 33.3 ± 5.0 | - | - | - | - | - | - | - | - | - |
| Ohlsson et al. (2015) [32] | DOM D1 | | All | Mean HR | 168 ± 9  (89 ± 3%) | 169 ± 9  (90 ± 3%) | 167 ± 9  (89 ± 3) | - | - | - | - | - | - | - |
|  |  |  |  | Peak HR | 189 ± 8  (100 ± 0%) | 188 ± 8  (100 ± 1%) | 186 ± 8  (98 ± 2) | - | - | - | - | - | - | - |
|  |  |  |  | Time (%)  0-60% HR | 0 | 1 | 0 | - | - | - | - | - | - | - |
|  |  |  |  | Time (%) 60-75% HR | 5 | 4 | 6 | - | - | - | - | - | - | - |
|  |  |  |  | Time (%) 75-85% HR | 18 | 13 | 18 | - | - | - | - | - | - | - |
|  |  |  |  | Time (%) 85-90% HR | 25 | 24 | 23 | - | - | - | - | - | - | - |
|  |  |  |  | Time (%) 90-95% HR | 32 | 37 | 35 | - | - | - | - | - | - | - |
|  |  |  |  | Time (min) 95-100% HR | 19 | 21 | 17 | - | - | - | - | - | - | - |
| Panduro et al. (2021) [74] | DOM D1 | | GK | Mean HR | 148 ± 10  (79 ± 5%) | - | - | - | - | - | - | - | - | - |
|  |  |  |  | Peak HR | 181 ± 11  (96 ± 4%) | - | - | - | - | - | - | - | - | - |
|  |  |  | CD | Mean HR | 169 ± 9  (87 ± 4%) | 170 ± 9 | 167 ± 9 | 184 ± 9 | - | - | - | - | 183 ± 8 | 181 ± 8 |
|  |  |  |  | Peak HR | 192 ± 8  (98 ± 2%) | 192 ± 9 | 190 ± 9 | - | - | - | - | - | - | - |
|  |  |  | FB | Mean HR | 171 ± 11  (89 ±3%) | 172 ± 11 | 170 ± 11 | 184 ± 10 | - | - | - | - | 183 ± 10 | 182 ± 10 |
|  |  |  |  | Peak HR | 190 ± 9  (99 ± 1%) | 190 ± 9 | 187 ± 10 | - | - | - | - | - | - | - |
|  |  |  | CM | Mean HR | 170 ± 10  (89 ± 3%) | 172 ± 10 | 168 ± 11 | 183 ± 11 | - | - | - | - | 182 ± 12 | 181 ± 11 |
|  |  |  |  | Peak HR | 190 ± 11  (99 ± 2%) | 190 ± 11 | 189 ± 11 | - | - | - | - | - | - | - |
|  |  |  | WM | Mean HR | 173 ± 8  (89 ± 4%) | 174 ± 9 | 171 ± 8 | 187 ± 12 | - | - | - | - | 185 ± 9 | 183 ± 9 |
|  |  |  |  | Peak HR | 193 ± 13  (99 ± 1%) | 193 ± 13 | 191 ± 13 | - | - | - | - | - | - | - |
|  |  |  | FWD | Mean HR | 170 ± 8  (87 ± 3%) | 171 ± 9 | 169 ± 7 | 185 ± 8 | - | - | - | - | 185 ± 7 | 182 ± 7 |
|  |  |  |  | Peak HR | 194 ± 6  (98 ± 3%) | 193 ± 6 | 190 ± 6 | - | - | - | - | - | - | - |
| Paulsen et al. (2018) [45] | COL D1 | | All | Mean HR | 172 | - | - | 164 ± 16* | 180 ± 5* | 177 ± 5* | 172 ± 11* | 174 ± 8* | 171 ± 10* | - |
|  |  |  | DEF | Mean HR | 176 ± 5 | - | - | - | - | - | - | - | - | - |
|  |  |  | MID | Mean HR | 171 ± 8 | - | - | - | - | - | - | - | - | - |
|  |  |  | FWD | Mean HR | 172 ± 3 | - | - | - | - | - | - | - | - | - |
| Williams et al. (2019) [42] | COL D1 | | All | Mean HR | - | - | - | 168 ± 24 | 169 ± 26 | 167 ± 26 | 163 ± 24 | 162 ± 25 | 166 ± 24 | - |

Data presented as mean ± SD. *Data presented as mean ± SE. Sample/Group: COL=college; DOM=domestic; INT=international; D=division; IC=in-conference competition; OC=out-of-conference competition. Playing Position: GK=goalkeeper; DEF=defender; CD=central defender; FB=full-back; MID=midfielder; CM=central midfielder; WM=wide midfielder; FP=flank player; FWD=forward. HR=heart rate.
