# Supplementary material for: A systematic review of match-play characteristics in women’s soccer
Source: PLoS One. 2022 Jun 30;17(6):e0268334. doi: 10.1371/journal.pone.0268334 (PMC9246157; doi:10.1371/journal.pone.0268334)
Supplement: S2 Table — (DOCX) [file pone.0268334.s003.docx]

**Table S2** Whole-match high-speed running and sprinting match-play characteristics of women’s soccer players

| **Study** | **Sample/ Group** | | **Velocity Thresholds (km∙h^-1^)** | **Repeat HSR or SPR**  **(Y/N)** | **Playing Position** | **HSR** | | | | | **SPR** | | | | |
| --- | --- | --- | --- | --- | --- | --- | --- | --- | --- | --- | --- | --- | --- | --- | --- |
|  |  |  |  |  |  | **Efforts (n)** | **Efforts (n∙min^-1^)** | **Distance per Effort (m)** | **Effort Duration (s)** | **Recovery Duration (s)** | **Efforts (n)** | **Efforts (n∙min^-1^)** | **Distance per Effort (m)** | **Effort Duration (s)** | **Recovery Duration (s)** |
| Andersen et al. (2016) [31] | DOM D1-D3 | | HSR: 16.1-20  SPR: >20 | N | All | 86 ± 21 | - | - | - | - | - | - | 12 ± 7 | - | - |
|  |  |  |  | N | All | - | - | - | - | - | - | - | - | - | - |
| Andersson et al. (2010) [43] | INT | | HSR: >15 | N | DEF | 149 ± 15* | - | - | - | - | - | - | 21 ± 3* | - | - |
|  |  |  |  |  | MID | 239 ± 30* | - | - | - | - | - | - | 27 ± 4* | - | - |
|  |  |  |  |  | FWD | 208 ± 29* | - | - | - | - | - | - | 25 ± 5* | - | - |
|  | DOM D1 | |  | N | DEF | 151 ± 14* | - | - | - | - | - | - | 19 ± 3* | - | - |
|  |  |  |  |  | MID | 193 ± 21* | - | - | - | - | - | - | 20 ± 3* | - | - |
|  |  |  |  |  | FWD | 180 ± 31* | - | - | - | - | - | - | 18 ± 3* | - | - |
| Bozzini et al. (2020) [50] | COL D1 | IC | SPR: >20 | N | All | - | - | - | - | - | - | 0.15 ± 0.07 | - | - | - |
|  |  | OC |  |  | All | - | - | - | - | - | - | 0.16 ± 0.05 | - | - | - |
| Datson et al. (2019) [52] | INT | | HSR: >19.8  SPR: >25.1 | N | All | 169 ± 49 | - | - | - | 41 ± 12 | 33 ± 13 | - | - | - | - |
|  |  |  |  |  | CD | 119 ± 22 | - | - | - | 54 ± 9 | 22 ± 7 | - | - | - | - |
|  |  |  |  |  | WD | 170 ± 45 | - | - | - | 40 ± 9 | 32 ± 14 | - | - | - | - |
|  |  |  |  |  | CM | 190 ± 46 | - | - | - | 36 ± 9 | 35 ± 12 | - | - | - | - |
|  |  |  |  |  | WM | 197 ± 46 | - | - | - | 35 ± 8 | 40 ± 14 | - | - | - | - |
|  |  |  |  |  | ATT | 189 ± 36 | - | - | - | 38 ± 8 | 42 ± 8 | - | - | - | - |
|  |  |  |  | Y | All | 33 ± 10 | - | 6.5 ± 1.1 | - | 169 ± 62 | 1.1 ± 1.1 | - | 4.9 ± 1.3 | - | 700 ± 547 |
|  |  |  |  |  | CD | 22 ± 5 | - | 5.9 ± 1.0 | - | 236 ± 62 | 0.6 ± 0.7 | - | 5.1 ± 1.4 | - | N/A |
|  |  |  |  |  | WD | 33 ± 8 | - | 6.8 ± 0.9 | - | 166 ± 52 | 0.9 ± 0.9 | - | 5.0 ± 1.4 | - | 834 ± 544 |
|  |  |  |  |  | CM | 38 ± 8 | - | 6.3 ± 0.9 | - | 141 ± 40 | 1.6 ± 1.2 | - | 4.8 ± 1.4 | - | 697 ± 564 |
|  |  |  |  |  | WM | 40 ± 14 | - | 7.0 ± 1.5 | - | 137 ± 42 | 1.4 ± 1.3 | - | 5.0 ± 1.2 | - | 790 ± 822 |
|  |  |  |  |  | ATT | 42 ± 8 | - | 6.9 ± 0.7 | - | 142 ± 35 | 1.4 ± 1.4 | - | 4.3 ± 0.6 | - | 497 ± 351 |
| Gabbett et al. (2008) [34] | INT | | Qualitative | Y | All | - | - | - | - | - | 4.8 ± 2.8 | - | - | 2.1 ± 0.7 | 1164 ± 1344 |
|  | DOM D1 | |  | Y | All | - | - | - | - | - | 1.0 ± 1.0 | - | - | 2.9 ± 1.1 | N/A |
| Gabbett et al. (2013) [54] | INT | | Qualitative | Y | DEF | 21 ± 14 | - | - | - | - | 3.2 ± 2.3 | - | - | - | - |
|  |  |  |  |  | CM | 40 ± 35 | - | - | - | - | 10.0 ± 11.3 | - | - | - | - |
|  |  |  |  |  | WM | 6 | - | - | - | - | 6 | - | - | - | - |
|  |  |  |  |  | STR | 27 ± 9 | - | - | - | - | 3.6 ± 2.3 | - | - | - | - |
|  | DOM D1 | |  | Y | DEF | 32 ± 21 | - | - | - | - | 5.3 ± 4.4 | - | - | - | - |
|  |  |  |  |  | CM | 38 ± 20 | - | - | - | - | 7.4 ± 4.5 | - | - | - | - |
|  |  |  |  |  | WM | 25 ± 9 | - | - | - | - | 1.0 ± 1.0 | - | - | - | - |
|  |  |  |  |  | STR | 42 ± 21 | - | - | - | - | 6.0 ± 6.7 | - | - | - | - |
| Jagim et al. (2020) [62] | COL D3 | | SPR: >19 | N | All | - | - | - | - | - | 15 ± 8 | - | - | - | - |
|  |  |  |  |  | GK | - | - | - | - | - | 5 ± 3 | - | - | - | - |
|  |  |  |  |  | CB | - | - | - | - | - | 16 ± 7 | - | - | - | - |
|  |  |  |  |  | CM | - | - | - | - | - | 17 ± 8 | - | - | - | - |
|  |  |  |  |  | FP | - | - | - | - | - | 18 ± 7 | - | - | - | - |
|  |  |  |  |  | FWD | - | - | - | - | - | 11 ± 5 | - | - | - | - |
| Julian et al. (2020) [63] | DOM D1-2 | FP | HSR: 16.69 ± 1.1 - 19.94 ± 0.9  SPR: >19.94 ± 0.9 | N | All | 150 ± 29 | - | - | - | - | 23 ± 13 | - | 9 ± 2 | - | - |
|  |  | LP |  | N | All | 156 ± 29 | - | - | - | - | 25 ± 12 | - | - | - | - |
| Krustrup et al. (2005) [65] | DOM D1 | | HSR: >15  SPR: >25 | N | All | 125 | - | - | - | 2.3 | 26 | - | - | - | - |
| Mara et al. (2017) [70] | DOM D1 | | HSR: 12.24 – 19.44  SPR: >19.44 | N | All | 376 ± 90 | - | 7 ± 1 | - | 42 ± 10 | 70 ± 29 | - | 7.7 ± 2.5 | - | 87 ± 38 |
|  |  |  |  | Y | All | 297 ± 93 | - | 6.3 ± 0.7 | - | - | 25 ± 17 | - | - | - | - |
| McCormack et al. (2015) [71] | COL D1 | >42h | HSR: 12.29 – 21.99  SPR: >21.99 | N | All | 138 ± 36 | - | - | - | - | 4.3 ± 3.5 | - | - | - |  |
|  |  | <42h |  | N | All | 127 ± 31 | - | - | - | - | 4.7 ± 3.6 | - | - | - | - |
|  |  |  |  |  | All |  | - | - | - | - | - | - | - | - | - |
| McFadden et al. (2020) [29] | COL D1 | | HSR: 15 – 18.99  SPR: >19 | N | All | - | - | - | - | - | 14 ± 5 | - | - | - | - |
| Meylan et al. (2017) [72] | INT | | HSR: 16.5 – 19.9  SPR: >20 | N | All | - | 0.73 ± 0.25 | - | - | - | - | - | - | - | - |
| Mohr et al. (2008) [44] | Top-class | | HSR: >15  SPR: >25 | N | All | 154 ± 7* | - | - | 2.1 ± 0.1* | - | 30 ± 2* | - | - | 2.1 ± 0.1* | - |
|  | High-level | |  | N | All | 125 ± 7* | - | - | 2.1 ± 0.3* | - | 26 ± 1* | - | - | 2.1 ± 0.1* | - |
| Nakamura et al. (2017) [73] | INT | | SPR: >20 | N | All | - | - | - | - | - | 18 ± 9 | - | 16 ± 2 | 2.5 ± 0.5 | 350 ± 206 |
|  |  |  |  |  | CD | - | - | - | - | - | 8 ± 3 | - | 15 ± 2 | 2.4 ± 0.6 | 533 ± 251 |
|  |  |  |  |  | FB | - | - | - | - | - | 21 ± 5 | - | 17 ± 3 | 2.7 ± 0.4 | 265 ± 55 |
|  |  |  |  |  | MID | - | - | - | - | - | 22 ± 10 | - | 16 ± 3 | 2.5 ± 0.4 | 283 ± 161 |
|  |  |  |  |  | FWD | - | - | - | - | - | 23 ± 8 | - | 15 ± 3 | 2.5 ± 0.4 | 247 ± 58 |
|  |  |  | SPR: >19.37 ± 0.48 |  | All | - | - | - | - | - | 22 ± 12 | - | 16 ± 3 | 2.6 ± 0.4 | 311 ± 188 |
|  |  |  |  |  | CD | - | - | - | - | - | 10 ± 4 | - | 15 ± 2 | 2.4 ± 0.3 | 490 ± 212 |
|  |  |  |  |  | WD | - | - | - | - | - | 28 ± 6 | - | 18 ± 2 | 3.0 ± 0.4 | 202 ± 39 |
|  |  |  |  |  | MID | - | - | - | - | - | 22 ± 11 | - | 17 ± 3 | 2.7 ± 0.4 | 278 ± 140 |
|  |  |  |  |  | FWD | - | - | - | - | - | 32 ± 11 | - | 15 ± 3 | 2.6 ± 0.4 | 191 ± 51 |
| Trewin et al. (2018) [20] | INT | | HSR: >16.48  SPR: >19.98 | N | All | 62 ± 20 | 0.64 ± 0.21 | - | - | - | 20 ± 9 | 0.21 ± 0.10 | - | - | - |
|  |  |  |  |  | CB | 44 ± 14 | 0.46 ± 0.15 | - | - | - | 14 ± 6 | 0.14 ± 0.06 | - | - | - |
|  |  |  |  |  | FB | 74 ± 16 | 0.78 ± 0.17 | - | - | - | 26 ± 9 | 0.28 ± 0.10 | - | - | - |
|  |  |  |  |  | MID | 67 ± 19 | 0.70 ± 0.20 | - | - | - | 20 ± 9 | 0.20 ± 0.09 | - | - | - |
|  |  |  |  |  | FWD | 67 ± 17 | 0.70 ± 0.18 | - | - | - | 25 ± 9 | 0.26 ± 0.09 | - | - | - |
| Trewin et al. (2018) [84] | DOM D1 | Sea-Level | HSR: >16.48  SPR: >19.98 | N | All | - | 0.64 ± 0.19 | - | - | - | - | 0.21 ± 0.10 | - | - | - |
|  |  | Altitude |  |  | All | - | 0.60 ± 0.17 | - | - | - | - | 0.21 ± 0.08 | - | - | - |
|  |  | Cold/mild |  |  | All | - | 0.65 ± 0.19 | - | - | - | - | 0.22 ± 0.10 | - | - | - |
|  |  | Warm/hot |  |  | All | - | 0.60 ± 0.17 | - | - | - | - | 0.21 ± 0.09 | - | - | - |
|  |  | Win (W) |  |  | All | - | 0.63 ± 0.20 | - | - | - | - | 0.21 ± 0.10 | - | - | - |
|  |  | Draw (D) |  |  | All | - | 0.58 ± 0.20 | - | - | - | - | 0.20 ± 0.11 | - | - | - |
|  |  | Loss (L) |  |  | All | - | 0.67 ± 0.16 | - | - | - | - | 0.08 ± 0.23 | - | - | - |
|  |  | W vs higher ranked OPP |  |  | All | - | 0.65 ± 0.18 | - | - | - | - | 0.22 ± 0.10 | - | - | - |
|  |  | D vs higher ranked OPP |  |  | All | - | 0.53 ± 0.21 | - | - | - | - | 0.17 ± 0.11 | - | - | - |
|  |  | L vs higher ranked OPP |  |  | All | - | 0.66 ± 0.16 | - | - | - | - | 0.22 ± 0.08 | - | - | - |
|  |  | W vs lower ranked OPP |  |  | All | - | 0.63 ± 0.20 | - | - | - | - | 0.21 ± 0.10 | - | - | - |
|  |  | D vs lower ranked OPP |  |  | All | - | 0.67 ± 0.14 | - | - | - | - | 0.27 ± 0.07 | - | - | - |
|  |  | L vs lower ranked OPP |  |  | All | - | 0.69 ± 0.15 | - | - | - | - | 0.25 ± 0.08 | - | - | - |
|  |  | >72 hours |  |  | All | - | 0.63 ± 0.16 | - | - | - | - | 0.21 ± 0.10 | - | - | - |
|  |  | <72 hours |  |  | All | - | 0.65 ± 0.20 | - | - | - | - | 0.23 ± 0.09 | - | - | - |
| Vescovi (2012) [86] | DOM D1 | | SPR: >18 | N | All | - | - | - | - | - | - | - | 15 ± 9 | 2.3 ± 1.5 | 150 ± 150 |
|  |  |  |  |  | DEF | - | - | - | - | - | - | - | 15 ± 9 | 2.3 ± 1.5 | 150 ± 144 |
|  |  |  |  |  | MID | - | - | - | - | - | - | - | 14 ± 9 | 2.2 ± 1.4 | 168 ± 180 |
|  |  |  |  |  | FWD | - | - | - | - | - | - | - | 16 ± 10 | 2.3 ± 1.5 | 126 ± 120 |
| Vescovi (2014) [40] | U17 DOM | | SPR: >20 | N | All | - | - | - | - | - | 13 ± 2 | - | 19 ± 2 | - | - |
|  | U16 DOM | |  |  | All | - | - | - | - | - | 11 ± 1 | - | 17 ± 1 | - | - |
|  | U15 DOM | |  |  | All | - | - | - | - | - | 5 ± 2 | - | 16 ± 2 | - | - |
|  | U15 – U17 DOM | |  |  | DEF | - | - | - | - | - | 11 ± 1 | - | 17 ± 1 | - | - |
|  |  |  |  |  | MID | - | - | - | - | - | 8 ± 1 | - | 18 ± 1 | - | - |
|  |  |  |  |  | FWD | - | - | - | - | - | 15 ± 2 | - | 17 ± 2 | - | - |

Data presented as mean ± SD. *Data presented as mean ± SE. HSR=high-speed running; SPR=sprinting. Qualitative VT = HSR “striding; movement is similar to jogging but involves a longer stride and more pronounced arm swing”; SPR “maximal effort with a greater extension of the lower leg during forward swing and higher heel lift relative to striding”. Sample/Group: COL=college; DOM=domestic; INT=international; U=Under; D=division. Playing Position: GK=goalkeeper; DEF=defender; CD=central defender; WD=wide defender; MID=midfield; CM=central midfield; WM=wide midfield; FP=flank player; ATT=attacker; FWD=forward; STR=striker.
