# Supplementary material for: A systematic review of match-play characteristics in women’s soccer
Source: PLoS One. 2022 Jun 30;17(6):e0268334. doi: 10.1371/journal.pone.0268334 (PMC9246157; doi:10.1371/journal.pone.0268334)
Supplement: S3 Table — (DOCX) [file pone.0268334.s004.docx]

**Table S3** Whole-match acceleration and deceleration characteristics of women’s soccer players

| **Study** | **Sample/ Group** | **Thresholds**  **(m∙s^-2^)** | **Playing Position** | **ACC** | | **DEC** | |
| --- | --- | --- | --- | --- | --- | --- | --- |
|  |  |  |  | **Efforts (n)** | **Total Duration (s)** | **Efforts (n)** | **Total Duration (s)** |
| Griffin et al. (2021) [57] | INT | 1 - 2 | All | - | 524 ± 25 | - | 544 ± 28 |
|  |  | 2 – 3 | All | - | 164 ± 14 | - | 162 ± 13 |
|  |  | 3 – 4 | All | - | 51 ± 5 | - | 54 ± 5 |
|  |  | ACC: >4  DEC: <4 | All | - | 39 ± 4 | - | 42 ± 4 |
|  | DOM | 1 - 2 | All | - | 554 ± 26 | - | 530 ± 30 |
|  |  | 2 – 3 | All | - | 188 ± 15 | - | 181 ± 14 |
|  |  | 3 – 4 | All | - | 72 ± 6 | - | 74 ± 5 |
|  |  | ACC: >4  DEC: <4 | All | - | 32 ± 4 | - | 39 ± 4 |
| Jagim et al. (2020) [62] | COL D3 | 0.5 – 1.99 | All | 953 ± 260 | - | 1010 ± 266 | - |
|  |  |  | GK | 900 ± 323 | - | 1006 ± 343 | - |
|  |  |  | CD | 996 ± 232 | - | 1057 ± 236 | - |
|  |  |  | CM | 991 ± 250 | - | 1038 ± 252 | - |
|  |  |  | FP | 919 ± 264 | - | 970 ± 274 | - |
|  |  |  | FWD | 763 ± 183 | - | 820 ± 190 | - |
|  |  | 2 – 2.99 | All | 64 ± 23 | - | 69 ± 27 | - |
|  |  |  | GK | 27 ± 11 | - | 23 ± 10 | - |
|  |  |  | CD | 67 ± 19 | - | 72 ± 19 | - |
|  |  |  | CM | 70 ± 22 | - | 77 ± 24 | - |
|  |  |  | FP | 69 ± 21 | - | 74 ± 24 | - |
|  |  |  | FWD | 51 ± 14 | - | 55 ± 15 | - |
|  |  | ACC: >3  DEC: <3 | All | 10 ± 6 | - | 17 ± 8 | - |
|  |  |  | GK | 3 ± 3 | - | 4 ± 3 | - |
|  |  |  | CD | 10 ± 5 | - | 14 ± 6 | - |
|  |  |  | CM | 11 ± 6 | - | 12 ± 6 | - |
|  |  |  | FP | 12 ± 5 | - | 17 ± 8 | - |
|  |  |  | FWD | 7 ± 4 | - | 10 ± 5 | - |
| Mara et al. (2017) [70] | DOM D1 | ACC: LSR to LSR  DEC: LSR to LSR | CD | 213 ± 79 | - | 204 ± 54 | - |
|  |  |  | WD | 237 ± 73 | - | 254 ± 74 | - |
|  |  |  | MID | 266 ± 72 | - | 280 ± 72 | - |
|  |  |  | CATT | 198 ± 59 | - | 204 ± 54 | - |
|  |  |  | WATT | 252 ± 64 | - | 254 ± 56 | - |
|  |  | ACC: LSR to HSR  DEC: HSR to LSR | CD | 79 ± 30 | - | 83 ± 30 | - |
|  |  |  | WD | 108 ± 27 | - | 107 ± 32 | - |
|  |  |  | MID | 123 ± 44 | - | 122 ± 43 | - |
|  |  |  | CATT | 116 ± 44 | - | 113 ± 41 | - |
|  |  |  | WATT | 124 ± 23 | - | 129 ± 22 | - |
|  |  | ACC: HSR to HSR  DEC: HSR to HSR | CD | 20 ± 9 | - | 17 ± 7 | - |
|  |  |  | WD | 32 ± 9 | - | 28 ± 14 | - |
|  |  |  | MID | 32 ± 8 | - | 28 ± 8 | - |
|  |  |  | CATT | 35 ± 9 | - | 30 ± 12 | - |
|  |  |  | WATT | 34 ± 9 | - | 129 ± 22 | - |
|  |  | ACC: HSR to SPR  DEC: SPR to HSR | CD | 11 ± 6 | - | 11 ± 5 | - |
|  |  |  | WD | 21 ± 10 | - | 19 ± 9 | - |
|  |  |  | MID | 21 ± 9 | - | 14 ± 8 | - |
|  |  |  | CATT | 26 ± 14 | - | 19 ± 10 | - |
|  |  |  | WATT | 27 ± 7 | - | 22 ± 7 | - |
|  |  | ACC: SPR to SPR  DEC: SPR to SPR | CD | 7 ± 4 | - | 4 ± 3 | - |
|  |  |  | WD | 10 ± 7 | - | 6 ± 6 | - |
|  |  |  | MID | 5 ± 3 | - | 5 ± 3 | - |
|  |  |  | CATT | 14 ± 3 | - | 9 ± 5 | - |
|  |  |  | WATT | 12 ± 3 | - | 11 ± 6 | - |
|  |  | ACC: LSR to SPR  DEC: SPR to LSR | CD | 12 ± 7 | - | 16 ± 8 | - |
|  |  |  | WD | 23 ± 18 | - | 29 ± 11 | - |
|  |  |  | MID | 18 ± 9 | - | 24 ± 10 | - |
|  |  |  | CATT | 24 ± 14 | - | 34 ± 15 | - |
|  |  |  | WATT | 26 ± 11 | - | 29 ± 9 | - |
| Panduro et al. (2021) [74] | DOM D1 | ACC: >0.5  DEC: <0.5 | GK | 695 ± 164 | - | 738 ± 139 | - |
|  |  |  | CD | 864 ± 114 | - | 887 ± 101 | - |
|  |  |  | FB | 878 ± 136 | - | 895 ± 137 | - |
|  |  |  | CM | 945 ± 140 | - | 946 ± 135 | - |
|  |  |  | WM | 871 ± 116 | - | 893 ± 123 | - |
|  |  |  | FWD | 884 ± 126 | - | 921 ± 148 | - |

Data presented as mean ± SD. ACC=accelerations; DEC=decelerations. Sample/Group: COL=college; DOM=domestic; INT=international; D=division. Thresholds: LSR=low-speed running; HSR=high-speed running; SPR=sprinting. Playing Position: GK=goalkeeper; CD=central defender; FB=full-back; MID=midfield; CM=central midfield; WM=wide midfield; FP=flank player; FWD=forward; CATT=central attacker; WATT=wide attacker.
