# Supplementary material for: A systematic review of match-play characteristics in women’s soccer
Source: PLoS One. 2022 Jun 30;17(6):e0268334. doi: 10.1371/journal.pone.0268334 (PMC9246157; doi:10.1371/journal.pone.0268334)
Supplement: S4 Table — (DOCX) [file pone.0268334.s005.docx]

**Table S4** Half-match physical characteristics of women’s soccer match-play

| **Study** | **Group / Sample** | | | **Velocity (km∙h^-1^) or Acceleration (m∙s^-2^) Thresholds** | **Playing Position** | **Half** | **TD**  **(m)** | **TD**  **(m∙min^-1^)** | **HSR**  **(m)** | **HSR (m∙min^-1^)** | **VHSR**  **(m)** | **SPR**  **(m)** | **SPR**  **(m∙min^-1^)** | **Vmax (km∙h^-1^)** | **ACC (n)** |
| --- | --- | --- | --- | --- | --- | --- | --- | --- | --- | --- | --- | --- | --- | --- | --- |
| Andersson et al. (2010) [43] | INT | | | HSR: >15  SPR: >25 | All | 1^st^ | 5000 ± 900* | - | 820 ± 50* | - | - | 136 ± 3* | - | - | - |
|  |  |  |  |  |  | 2^nd^ | 4900 ± 1000* | - | 720 ± 50* | - | - | 120 ± 3* | - | - | - |
|  | DOM D1 | | |  | All | 1^st^ | 4900 ± 800* | - | 710 ± 50* | - | - | - | - | - | - |
|  |  |  |  |  |  | 2^nd^ | 4800 ± 800* | - | 620 ± 40* | - | - | - | - | - | - |
| Bohner et al. (2015) [49] | COL D1 | Sea-level | | HSR: >15 | All | 1^st^ | - | 123 ± 8 | - | 28 ± 9 | - | - | - | - | - |
|  |  |  |  |  |  | 2^nd^ | - | 119 ± 9 | - | 27 ± 10 | - | - | - | - | - |
|  |  | Altitude | |  | All | 1^st^ | - | 109 ± 8 | - | 25 ± 8 | - | - | - | - | - |
|  |  |  |  |  |  | 2^nd^ | - | 106 ± 7 | - | 25 ± 7 | - | - | - | - | - |
| Bozzini et al. (2020) [50] | COL D1 | In-conference | | HSR: 15 - 19.9  SPR: >20 | All | 1^st^ | - | 108 ± 9 | - | 11 ± 2 | - | - | 3.2 ± 1.9 | - | - |
|  |  |  |  |  |  | 2^nd^ | - | 99 ± 9 | - | 9 ± 2 | - | - | 2.9 ± 1.8 | - | - |
|  |  | Out-conference | |  | All | 1^st^ | - | 109 ± 19 | - | 11 ± 3 | - | - | 3.3 ± 2.8 | - | - |
|  |  |  |  |  |  | 2^nd^ | - | 101 ± 9 | - | 9 ± 2 | - | - | 2.9 ± 1.7 | - | - |
| Bradley et al. (2014) [26] | DOM UEFA CL | | | HSR: >15 | All | 1^st^ | 5486** | - | 397 ± 19* | - | - | - | - | - | - |
|  |  |  |  |  |  | 2^nd^ | 5267** | - | 380 ± 19* | - | - | - | - | - | - |
|  |  |  |  |  | CD | 1^st^ | 5230** | - | 294 ± 21* | - | - | - | - | - | - |
|  |  |  |  |  |  | 2^nd^ | 5007** | - | 308 ± 31* | - | - | - | - | - | - |
|  |  |  |  |  | FB | 1^st^ | 5436** | - | 398 ± 48* | - | - | - | - | - | - |
|  |  |  |  |  |  | 2^nd^ | 5269** | - | 357 ± 46* | - | - | - | - | - | - |
|  |  |  |  |  | CM | 1^st^ | 5724** | - | 397 ± 25* | - | - | - | - | - | - |
|  |  |  |  |  |  | 2^nd^ | 5434** | - | 382 ± 31* | - | - | - | - | - | - |
|  |  |  |  |  | WM | 1^st^ | 5595** | - | 461 ± 56* | - | - | - | - | - | - |
|  |  |  |  |  |  | 2^nd^ | 5334** | - | 470 ± 38* | - | - | - | - | - | - |
|  |  |  |  |  | ATT | 1^st^ | 5423** | - | 566 ± 39* | - | - | - | - | - | - |
|  |  |  |  |  |  | 2^nd^ | 5344** | - | 485 ± 53* | - | - | - | - | - | - |
| Gabbett & Mulvey (2008) [34] | INT | | | Qualitative | All | 1^st^ | 5213 ± 735 | - | - | - | - | - | - | - | - |
|  |  |  |  |  |  | 2^nd^ | 4755 ± 699 | - | - | - | - | - | - | - | - |
| Hewitt et al. (2014) [38] | INT | | | HSR: >12  SPR: >19 | All | 1^st^ | 4936 ± 594 | - | 1244 ± 465 | - | - | 173 ± 114 | - | - | - |
|  |  |  |  |  |  | 2^nd^ | 4695 ± 823 | - | 1163 ± 541 | - | - | 165 ± 137 | - | - | - |
| Mara et al. (2017) [69] | DOM D1 | | | ACC: >2 | All | 1^st^ | - | - | - | - | - | - | - | - | 219** |
|  |  |  |  |  |  | 2^nd^ | - | - | - | - | - | - | - | - | 207** |
| Mara et al. (2017) [70] | DOM D1 | | | HSR: 12.24 – 19.44  SPR: >19.44  ACC: >2 | All | 1^st^ | 5183 ± 427 | - | 1305 ± 106 | - | - | 329 ± 136 | - | - | - |
|  |  |  |  |  |  | 2^nd^ | 4811 ± 387 | - | 1136 ± 101 | - | - | 284 ± 137 | - | - | - |
| Mohr et al. (2008) [44] | Top-Class | | | HSR: >15  SPR >25 | All | 1^st^ | 5280 ± 90* | - | 910 ± 50* | - | - | 250 ± 20* | - | - | - |
|  |  |  |  |  |  | 2^nd^ | 5050 ± 80* | - | 700 ± 40* | - | - | 210 ± 10* | - | - | - |
|  | High-Level | | |  | All | 1^st^ | 5220 ± 90* | - | 680 ± 60* | - | - | 200 ± 30* | - | - | - |
|  |  |  |  |  |  | 2^nd^ | 5210 ± 80* | - | 620 ± 40* | - | - | 170 ± 20* | - | - | - |
| Nakamura et al. (2017) [73] |  | | | SPR: >20 | All | 1^st^ | - | - | - | - | - | 154 ± 98 | - | - | - |
|  |  |  |  |  |  | 2^nd^ | - | - | - | - | - | 130 ± 88 | - | - | - |
|  |  |  |  | SPR: >19.37 ± 0.48 | All | 1^st^ | - | - | - | - | - | 190 ± 127 | - | - | - |
|  |  |  |  |  |  | 2^nd^ | - | - | - | - | - | 163 ± 100 | - | - | - |
| Panduro et al. (2021) [74] | DOM D1 | | | HSR: >15  VHSR: >18  SPR >25  ACC: >3 | CD | 1^st^ | 4663 ± 400 | - | 560 ± 133 | - | 232 ± 56 | 10 ± 11 | - | 25.9 ± 2.0 | 3.8 ± 2.1 |
|  |  |  |  |  |  | 2^nd^ | 4611 ± 394 | - | 528 ± 144 | - | 210 ± 87 | 9 ± 9 | - | 25.5 ± 1.7 | 2.9 ± 2.2 |
|  |  |  |  |  | FB | 1^st^ | 5031 ± 405 | - | 768 ± 201 | - | 367 ± 131 | 26 ± 25 | - | 27.0 ± 2.1 | 4.4 ± 3.0 |
|  |  |  |  |  |  | 2^nd^ | 5022 ± 28 | - | 761 ± 195 | - | 350 ± 121 | 19 ± 26 | - | 26.4 ± 1.5 | 3.6 ± 2.4 |
|  |  |  |  |  | CM | 1^st^ | 5283 ± 481 | - | 804 ± 252 | - | 328 ± 129 | 17 ± 14 | - | 26.3 ± 1.7 | 5.9 ± 4.4 |
|  |  |  |  |  |  | 2^nd^ | 5193 ± 544 | - | 714 ± 261 | - | 295 ± 134 | 16 ± 20 | - | 25.8 ± 1.9 | 4.1 ± 2.9 |
|  |  |  |  |  | EM | 1^st^ | 5283 ± 481 | - | 923 ± 242 | - | 459 ± 158 | 53 ± 52 | - | 27.1 ± 2.0 | 4.8 ± 4.0 |
|  |  |  |  |  |  | 2^nd^ | 4536 ± 524 | - | 863 ± 304 | - | 404 ± 169 | 39 ± 39 | - | 26.4 ± 1.9 | 2.3 ± 1.9 |
|  |  |  |  |  | FWD | 1^st^ | 4906 ± 560 | - | 813 ± 173 | - | 383 ± 95 | 28 ± 23 | - | 27.5 ± 2.3 | 7.6 ± 5.1 |
|  |  |  |  |  |  | 2^nd^ | 4839 ± 483 | - | 748 ± 221 | - | 353 ± 146 | 28 ± 24 | - | 27.4 ± 2.0 | 4.6 ± 2.7 |
| Park et al. (2019) [39] | INT | | | HSR: 12.5 - 19  VHSR: 19 – 22.5  SPR: >22.5 | All | Mean | - | - | 670 ± 215 | - | 94 ± 37 | 34 ± 18 | - | - | - |
| Principe et al. (2021) [77] | DOM D1 | | | HSR: 15.98 – 19.98  SPR: >19.98  ACC: >2 | DEF | 1^st^ | 4347 ± 1124 | - | 320 ± 116 | - | - | 164 ± 83 | - | - | 133 ± 38 |
|  |  |  |  |  |  | 2^nd^ | 3859 ± 1016 | - | 288 ± 102 | - | - | 138 ± 76 | - | - | 119 ± 37 |
|  |  |  |  |  | MID | 1^st^ | 4596 ± 1196 | - | 368 ± 150 | - | - | 158 ± 88 | - | - | 139 ± 43 |
|  |  |  |  |  |  | 2^nd^ | 3647 ± 1328 | - | 286 ± 137 | - | - | 119 ± 43 | - | - | 111 ± 46 |
|  |  |  |  |  | FWD | 1^st^ | 4465 ± 1040 | - | 327 ± 130 | - | - | 147 ± 105 | - | - | 128 ± 36 |
|  |  |  |  |  |  | 2^nd^ | 3138 ± 1226 | - | 240 ± 113 | - | - | 128 ± 36 | - | - | 92 ± 37 |
| Vescovi (2012) [86] | DOM D1 | | | SPR: >18 | DEF | 1^st^ | - | - | - | - | - | 278 ± 107 | - | - | - |
|  |  |  |  |  |  | 2^nd^ | - | - | - | - | - | 262 ± 131 | - | - | - |
|  |  |  |  |  | MID | 1^st^ | - | - | - | - | - | 232 ± 95 | - | - | - |
|  |  |  |  |  |  | 2^nd^ | - | - | - | - | - | 211 ± 108 | - | - | - |
|  |  |  |  |  | FWD | 1^st^ | - | - | - | - | - | 366 ± 100 | - | - | - |
|  |  |  |  |  |  | 2^nd^ | - | - | - | - | - | 287 ± 113 | - | - | - |
| Vescovi (2014) [40] | U17 DOM | | | HSR: 15.6 – 20  SPR: >20 | All | 1^st^ | 4322 ± 484 | 108 ± 12 | 345 ± 120 | - | - | 134 ± 77 | - | 24.9 ± 2.3 | - |
|  |  |  |  |  |  | 2^nd^ | 4236 ± 422 | 104 ± 12 | 314 ± 116 | - | - | 100 ± 66 | - | 25.5 ± 1.9 | - |
|  | U16 DOM | | |  | All | 1^st^ | 4084 ± 444 | 102 ± 8 | 314 ± 111 | - | - | 95 ± 71 | - | 25.1 ± 1.6 | - |
|  |  |  |  |  |  | 2^nd^ | 3941 ± 397 | 99 ± 8 | 296 ± 111 | - | - | 89 ± 63 | - | 24.6 ± 1.6 | - |
|  | U15 DOM | | |  | All | 1^st^ | 3480 ± 428 | 86 ± 10 | 224 ± 106 | - | - | 44 ± 70 | - | 23.2 ± 2.0 | - |
|  |  |  |  |  |  | 2^nd^ | 3478 ± 395 | 85 ± 13 | 234 ± 109 | - | - | 31 ± 60 | - | 23.7 ± 2.0 | - |
|  | U15 - U17 DOM | | |  | DEF | 1^st^ | 3946 ± 464 | 99 ± 15 | 298 ± 112 | - | - | 99 ± 67 | - | 25.1 ± 2.2 | - |
|  |  |  |  |  |  | 2^nd^ | 3831 ± 419 | 96 ± 7 | 292 ± 112 | - | - | 89 ± 60 | - | 24.8 ± 1.5 | - |
|  |  |  |  |  | MID | 1^st^ | 4281 ± 465 | 107 ± 10 | 313 ± 110 | - | - | 71 ± 70 | - | 23.9 ± 2.0 | - |
|  |  |  |  |  |  | 2^nd^ | 4170 ± 420 | 103 ± 10 | 287 ± 110 | - | - | 59 ± 60 | - | 23.9 ± 2.0 | - |
|  |  |  |  |  | FWD | 1^st^ | 4045 ±464 | 101 ± 11 | 363 ± 110 | - | - | 150 ± 71 | - | 25.8 ± 2.0 | - |
|  |  |  |  |  |  | 2^nd^ | 3907 ± 416 | 97 ± 11 | 302 ± 110 | - | - | 125 ± 103 | - | 26.1 ± 1.7 | - |
| Vescovi & Favero (2014) [41] | COL D1 | | | HSR: 15.6 – 20  SPR: >20 | DEF | 1^st^ | 4878 ± 339 | 100 ± 9 | 384 ± 115 | - | - | 131 ± 60 | - | - | - |
|  |  |  |  |  |  | 2^nd^ | 4618 ± 452 | 96 ± 9 | 364 ± 116 | - | - | 135 ± 67 | - | - | - |
|  |  |  |  |  | MID | 1^st^ | 5186 ± 340 | 106 ± 9 | 384 ± 119 | - | - | 87 ± 58 | - | - | - |
|  |  |  |  |  |  | 2^nd^ | 4939 ± 453 | 103 ± 11 | 378 ± 116 | - | - | 110 ± 67 | - | - | - |
|  |  |  |  |  | FWD | 1^st^ | 5232 ± 342 | 107 ± 7 | 475 ± 114 | - | - | 146 ± 58 | - | - | - |
|  |  |  |  |  |  | 2^nd^ | 5065 ± 453 | 106 ± 10 | 454 ± 115 | - | - | 193 ± 66 | - | - | - |
| Wells et al. (2015) [89] | COL D1 | | Regular season | HSR: 15.96 - 21.9  SPR >22 | All | 1^st^ | 3862 ± 560 | 105 ± 12 | 284 ± 78 | 8 ± 2 | - | 45 ± 46 | 1.2 ± 1.3 | 23.7 ± 2.2 | - |
|  |  |  |  |  |  | 2^nd^ | 3620 ± 454 | 98 ± 17 | 273 ± 63 | 8 ± 3 | - | 42 ± 35 | 1.2 ± 1.1 | 22.9 ± 1.9 | - |
|  |  |  | Post-season |  | All | 1^st^ | 4337 ± 397 | 99 ± 11 | 325 ± 85 | 7 ± 2 | - | 51 ± 52 | 1.1 ± 1.1 | 24.2 ± 1.5 | - |
|  |  |  |  |  |  | 2^nd^ | 3864 ± 418 | 98 ± 19 | 278 ± 62 | 7 ± 2 | - | 34 ± 33 | 0.8 ± 0.8 | 23.0 ± 3.5 | - |

Data presented as mean ± SD or mean (90% CI). *Data presented as mean ± SE. ** mean calculated from available data. TD=total distance; HSR=high-speed running; VHSR=very high-speed running; SPR=sprinting; Vmax=maximum velocity; ACC=accelerations. Qualitative VT = High-Speed Running “striding; movement is similar to jogging but involves a longer stride and more pronounced arm swing”; Sprinting “maximal effort with a greater extension of the lower leg during forward swing and higher heel lift relative to striding”. Sample/Group: COL=college; DOM=domestic; INT=international; U=Under; D=division; UEFA CL=UEFA Champions League. Playing Position: DEF=defender; CB=centre back; CD=central defender; FB=full-back; MID=midfield; CM=central midfield; WM=wide midfield; ATT=attacker; FWD=forward.
