# Supplementary material for: A systematic review of match-play characteristics in women’s soccer
Source: PLoS One. 2022 Jun 30;17(6):e0268334. doi: 10.1371/journal.pone.0268334 (PMC9246157; doi:10.1371/journal.pone.0268334)
Supplement: S5 Table — (DOCX) [file pone.0268334.s006.docx]

**Table S5** Segmental physical characteristics of women’s soccer-match-play

| **Physical Variable (Thresholds)** | | **Study** | **Group / Sample** | **Playing Position** | **Segmental Time Period** | | | | | | |
| --- | --- | --- | --- | --- | --- | --- | --- | --- | --- | --- | --- |
|  |  |  |  |  | **5-min** | **0-15min** | **15-30min** | **30-45min** | **45-60min** | **60-75min** | **75-90min** |
| **Total Distance (m)** | | Bendiksen et al. (2013) [35] | DOM D2 | All | - | 1782 ± 39 | - | - | - | 1393 ± 59 | 1315 ± 47 |
|  |  | Bradley et al. (2014) [26] | DOM UEFA CL | All | 569** | - | - | - | - | - | - |
|  |  | Hewitt et al. (2014) [38] | INT | All | - | 1699 ± 228 | 1570 ± 228 | 1567 ± 251 | 1595 ± 251 | 1506 ± 327 | 1458 ± 350 |
|  |  | Mara et al. (2017) [70] | DOM D1 | All | - | 1801 ± 143 | 1683 ± 199 | 1621 ± 168 | 1652 ± 168 | 1528 ± 168 | 1571 ± 161 |
|  |  | Mohr et al. (2008) [44] | Top-class | All | - | 1803 ± 32* | 1631 ± 40* | 1679 ± 28* | 1679 ± 32* | 1570 ± 32* | 1558 ± 36* |
|  |  |  | High-level | All | - | 1815 ± 32* | 1647 ± 36* | 1699 ± 40* | 1779 ± 32* | 1683 ± 44* | 1627 ± 36* |
|  |  | Panduro et al. (2021) [74] | DOM D1 | CD | - | 1572 ± 134 | - | - | - | - | 1396 ± 124 |
|  |  |  |  | FB | - | 1673 ± 125 | - | - | - | - | 1544 ± 132 |
|  |  |  |  | CM | - | 1796 ± 201 | - | - | - | - | 1556 ± 270 |
|  |  |  |  | WM | - | 1743 ± 175 | - | - | - | - | 1583 ± 137 |
|  |  |  |  | FWD | - | 1571 ± 210 | - | - | - | - | 1494 ± 157 |
|  |  | Ramos et al. (2017) [78] | INT U20 | CD | 463 ± 38 | 1528 ± 123 | 1377 ± 147 | 1347 ± 133 | 1383 ± 60 | 1342 ± 152 | 1185 ± 160 |
|  |  |  |  | WD | 495 ± 25 | 1800 ± 386 | 1671 ± 462 | 1631 ± 380 | 1718 ± 406 | 1505 ± 422 | 1456 ± 357 |
|  |  |  |  | MID | 464 ± 39 | 1583 ± 190 | 1461 ± 168 | 1402 ± 12 | 1459 ± 132 | 1370 ± 166 | 1242 ± 160 |
|  |  |  |  | FWD | 489 ± 26 | 1674 ± 147 | 1542 ± 165 | 1496 ± 122 | 1558 ± 100 | 1441 ± 157 | 1326 ± 101 |
| **Total Distance (m∙min^-1^)** | | Williams et al. (2019) [42] | COL D1 | All | - | 114 ± 19 | 104 ± 21 | 106 ± 19 | 105 ± 24 | 103 ± 24 | 104 ± 22 |
| **High Speed Running (m)** | 16.1 – 20 km∙h^-1^ | Andersen et al. (2016) [31] | DOM D1-D3 | All | - | 324 ± 102 | 247 ± 78 | 186 ± 59 | 259 ± 55 | 223 ± 63 | 199 ± 71 |
|  | 15.6 – 20 km∙h^-1^ | Ramos et al. (2017) [78] | INT U20 | CD | 31 ± 9 | 106 ± 32 | 77 ± 35 | 83 ± 34 | 79 ± 5 | 95 ± 28 | 68 ± 34 |
|  |  |  |  | WD | 45 ± 6 | 190 ± 56 | 147 ± 52 | 131 ± 28 | 166 ± 53 | 137 ± 72 | 143 ± 53 |
|  |  |  |  | MID | 28 ± 6 | 110 ± 43 | 97 ± 44 | 88 ± 30 | 91 ± 35 | 96 ± 48 | 74 ± 38 |
|  |  |  |  | FWD | 42 ± 12 | 163 ± 46 | 116 ± 40 | 129 ± 42 | 147 ± 53 | 150 ± 56 | 105 ± 29 |
|  | 15 – 25 km∙h^-1^ | Andersson et al. (2010) [43] | INT | All | 73 ± 35* | 257 ± 17* | 270 ± 16* | 260 ± 21* | 272 ± 26* | 216 ± 15* | 195 ± 17* |
|  |  |  | DOM D1 | All | 63 ± 31* | 233 ± 19* | 229 ± 23* | 235 ± 22* | 229 ± 17* | 195 ± 10* | 176 ± 22* |
|  |  | Krustrup et al. (2005) [65] | DOM D1 | All | - | 270 | - | 190 | 240 | - | 160 |
|  |  | Mohr et al. (2008) [44] | Top-class | All | - | 330 ± 23* | 266 ± 16* | 282 ± 21* | 281 ± 18* | 239 ± 13* | 209 ± 14* |
|  |  |  | High-level | All | - | 278 ± 29* | 224 ± 27* | 170 ± 22* | 232 ± 20* | 212 ± 25* | 164 ± 14* |
|  | 15 -21 km∙h^-1^ | Bendiksen et al. (2013) [35] | DOM D2 | All | - | 1782 ± 39 | - | - | - | 1393 ± 59 | 1315 ± 47 |
|  | > 15 km∙h^-1^ | Bradley et al. (2014) [26] | DOM UEFA CL | All | 80 ± 23 | - | - | - | - | - | - |
|  |  | Panduro et al. (2021) [74] | DOM D1 | CD | - | 216 ± 53 | - | - | - | - | 162 ± 60 |
|  |  |  |  | FB | - | 278 ± 77 | - | - | - | - | 235 ± 65 |
|  |  |  |  | CM | - | 296 ± 93 | - | - | - | - | 204 ± 98 |
|  |  |  |  | WM | - | 351 ± 95 | - | - | - | - | 246 ± 90 |
|  |  |  |  | FWD | - | 300 ± 86 | - | - | - | - | 222 ± 83 |
|  | > 14.4 km∙h^-1^ | Datson et al. (2017) [51] | INT | All | 135 ± 32 | 478 ± 120 | 439 ± 110 | 413 ± 117 | 439 ± 120 | 398 ± 106 | 355 ± 110 |
|  | 12.24 – 19.44 km∙h^-1^ | Mara et al. (2017) [70] | DOM D1 | All | - | 490 ± 128 | 411 ± 126 | 391 ± 109 | 393 ± 126 | 358 ± 123 | 371 ± 107 |
|  | 12 – 19 km∙h^-1^ | Hewitt et al. (2014) [38] | INT | All | - | 478 ± 183 | 384 ± 168 | 382 ± 168 | 429 ± 174 | 378 ± 198 | 356 ± 190 |
| **Interval Between High Speed Runs (s)** | 12.24 – 19.44 km∙h^-1^ | Mara et al. (2017) [70] | DOM D1 | All | - | 11 ± 3 | 14 ± 6 | 15 ± 5 | 14 ± 7 | 16 ± 5 | 16 ± 5 |
| **Repeated High Speed Run Efforts (n)**  ***(Qualitative VT)*** | 2 | Gabbett et al. (2013) [54] | INT | All | - | 7 | 6 | 3 | 5 | 4 | 4 |
|  |  |  | DOM D1 | All | - | 9 | 6 | 5 | 7 | 6 | 4 |
|  | 3 |  | INT | All | - | 6 | 4 | 2 | 4 | 3 | 3 |
|  |  |  | DOM D1 | All | - | 7 | 4 | 3 | 4 | 4 | 3 |
|  | 4+ |  | INT | All | - | 15 | 10 | 3 | 10 | 5 | 5 |
|  |  |  | DOM D1 | All | - | 20 | 7 | 6 | 10 | 9 | 6 |
| **Very High Speed Running (m)** | >18 km∙h^-1^ | Panduro et al. (2021) [74] | DOM D1 | CD | - | 90 ± 26 | - | - | - | - | 63 ± 33 |
|  |  |  |  | FB | - | 135 ± 52 | - | - | - | - | 96 ±37 |
|  |  |  |  | CM | - | 126 ± 54 | - | - | - | - | 81 ± 50 |
|  |  |  |  | WM | - | 175 ± 60 | - | - | - | - | 119 ± 57 |
|  |  |  |  | FWD | - | 149 ± 49 | - | - | - | - | 111 ± 54 |
| **Sprinting (m)** | > 25 km∙h^-1^ | Andersson et al. (2010) [41] | INT | All | 11 ± 2* | 41 ± 5* | 53 ± 5* | 39 ± 5* | 50 ± 7* | 34 ± 4* | 32 ± 8* |
|  |  |  | DOM D1 | All | 10 ± 2* | 40 ± 5* | 38 ± 7* | 36 ± 5* | 39 ± 5* | 34 ± 3* | 26 ± 4* |
|  |  | Mohr et al. (2008) [44] | Top-class | All | - | 81 ± 8* | 81 ± 9* | 79 ± 7* | 74 ± 7* | 71 ± 8* | 53 ± 6* |
|  |  |  | High-level | All | - | 86 ± 2* | 68 ± 3* | 54 ± 2* | 72 ± 3* | 63 ± 3* | 39 ± 2* |
|  |  | Panduro et al. (2021) [74] | DOM D1 | CD | - | 4 ± 6 | - | - | - | - | 2 ± 5 |
|  |  |  |  | FB | - | 11 ± 15 | - | - | - | - | 3 ± 5 |
|  |  |  |  | CM | - | 8 ± 9 | - | - | - | - | 3 ± 6 |
|  |  |  |  | WM | - | 21 ± 25 | - | - | - | - | 10 ± 16 |
|  |  |  |  | FWD | - | 11 ± 13 | - | - | - | - | 6 ± 9 |
|  | > 21 km∙h^-1^ | Bendiksen et al. (2013) [35] | DOM D2 | All | - | 71 ± 13 | - | - | 71 ± 13 | 39 ± 7 | 85 ± 12 |
|  | >20 km∙h^-1^ | Andersen et al. (2016) [31] | DOM D1-D3 | All | - | 111 ± 51 | 88 ± 44 | 61 ± 22 | 92 ± 51 | 77 ± 40 | 64 ± 37 |
|  |  | Ramos et al. (2017) [78] | INT U20 | CD | 6 ± 4 | 12 ± 9 | 16 ± 14 | 16 ± 20 | 14 ± 13 | 33 ± 23 | 25 ± 14 |
|  |  |  |  | WD | 15 ± 5 | 72 ± 35 | 42 ± 18 | 61 ± 29 | 57 ± 27 | 55 ± 46 | 48 ± 27 |
|  |  |  |  | MID | 5 ± 2 | 21 ± 19 | 18 ± 17 | 21 ± 19 | 28 ± 22 | 20 ± 24 | 16 ± 15 |
|  |  |  |  | FWD | 17 ± 6 | 64 ± 29 | 51 ± 41 | 53 ± 32 | 45 ± 26 | 63 ± 29 | 59 ± 36 |
|  | > 19.44 km∙h^-1^ | Mara et al. (2017) [70] | DOM D1 | All | - | 111 ± 61 | 107 ± 50 | 105 ± 51 | 97 ± 65 | 92 ± 49 | 92 ± 48 |
|  | > 19 km∙h^-1^ | Hewitt et al. (2014) [38] | INT | All | - | 67 ± 46 | 56 ± 46 | 50 ± 46 | 59 ± 53 | 53 ± 53 | 53 ± 61 |
| **Interval Between Sprints (s)** | > 19.44 km∙h^-1^ | Mara et al. (2017) [70] | DOM D1 | All | - | 68 ± 39 | 100 ± 68 | 85 ± 46 | 86 ± 53 | 108 ± 57 | 101 ± 55 |
| **Repeated Sprint Efforts (n)**  ***(Qualitative VT)*** | 2 | Gabbett et al. (2013) [54] | INT | All | - | 1.2 | 1.1 | 0.5 | 1.1 | 0.4 | 0.5 |
|  |  |  | DOM D1 | All | - | 0.8 | 1.1 | 1.3 | 0.8 | 0.8 | 0.6 |
|  | 3 |  | INT | All | - | 0.6 | 0.3 | 0.3 | 0.8 | 0.2 | 0.3 |
|  |  |  | DOM D1 | All | - | 0.5 | 0.5 | 0.5 | 0.5 | 0.4 | 0.2 |
|  | 4+ |  | INT | All | - | 1.9 | 0 | 0.1 | 1.1 | 0.2 | 0.7 |
|  |  |  | DOM D1 | All | - | 0.5 | 0.2 | 0.5 | 0.4 | 0.2 | 0 |
| **Maximum Velocity (km∙h^-1^)** | | Panduro et al. (2021) [74] | DOM D1 | CD | - | 24.7 ± 1.9 | - | - | - | - | 20.8 ± 1.5 |
|  |  |  |  | FB | - | 25.4 ± 2.1 | - | - | - | - | 22.0 ± 1.5 |
|  |  |  |  | CM | - | 25.2 ± 1.8 | - | - | - | - | 20.9 ± 2.8 |
|  |  |  |  | WM | - | 25.9 ± 1.7 | - | - | - | - | 22.6 ± 1.8 |
|  |  |  |  | FWD | - | 26.2 ± 2.5 | - | - | - | - | 22.1 ± 1.9 |
| **Accelerations (n)** | >3 m∙s^-2^ | Panduro et al. (2021) [74] | DOM D1 | CD | - | 1.7 ± 1.5 | - | - | - | - | 0.9 ± 0.9 |
|  |  |  |  | FB | - | 1.3 ± 1.3 | - | - | - | - | 0.9 ± 0.8 |
|  |  |  |  | CM | - | 1.8 ± 1.3 | - | - | - | - | 1.1 ± 1.0 |
|  |  |  |  | WM | - | 1.8 ± 2.1 | - | - | - | - | 0.6 ± 0.6 |
|  |  |  |  | FWD | - | 2.9 ± 2.2 | - | - | - | - | 1.3 ± 0.9 |
|  | >2 m∙s^-2^ | Ramos et al. (2017) [78] | INT U20 | CD | 0.6 ± 0.2 | 2.3 ± 1.6 | 2.3 ± 1.2 | 2.2 ± 1.8 | 1.7 ± 1.5 | 1.5 ± 1.4 | 1.8 ± 1.2 |
|  |  |  |  | WD | 0.7 ± 0.3 | 2.6 ± 1.7 | 3.3 ± 1.5 | 2.1 ± 1.5 | 3.1 ± 2.6 | 2.5 ± 2.1 | 2.5 ± 1.9 |
|  |  |  |  | MID | 0.7 ± 0.3 | 2.1 ± 1.6 | 2.7 ± 1.5 | 1.9 ± 1.3 | 2.2 ± 1.6 | 2.2 ± 1.6 | 2.5 ± 1.5 |
|  |  |  |  | FWD | 0.9 ± 0.3 | 2.7 ± 1.6 | 2.3 ± 2.1 | 3.4 ± 1.4 | 2.6 ± 1.4 | 2.7 ± 1.5 | 3.7 ± 3.4 |
| **Interval Between Accelerations (s)** | Mean | Mara et al. (2017) [69] | DOM D1 | All |  | 10 ± 5 | 15 ± 7 | 15 ± 5 | 14 ± 9 | 16 ± 5 | 16 ± 7 |
|  | Maximum |  |  |  |  | 74 ± 26 | 102 ± 55 | 101 ± 37 | 88 ± 36 | 106 ± 29 | 102 ± 42 |
| **Decelerations (n)** | >-3 m∙s^-2^ | Panduro et al. (2021) [74] | DOM D1 | CD |  | 2.3 ± 1.3 | - | - | - | - | 1.6 ± 1.3 |
|  |  |  |  | FB |  | 3.3 ± 1.7 | - | - | - | - | 2.6 ± 1.2 |
|  |  |  |  | CM |  | 3.1 ± 1.4 | - | - | - | - | 2.0 ± 1.2 |
|  |  |  |  | WM |  | 4.8 ± 2.4 | - | - | - | - | 3.1 ± 2.1 |
|  |  |  |  | FWD |  | 3.5 ± 2.0 | - | - | - | - | 2.8 ± 1.4 |
|  | >-2 m∙s^-2^ | Ramos et al. (2017) [78] | INT U20 | CD | 0.7 ± 0.2 | 2.3 ± 0.8 | 1.5 ± 1.4 | 1.8 ± 1.7 | 2.2 ± 1.6 | 2.5 ± 1.2 | 2.5 ± 1.1 |
|  |  |  |  | WD | 0.9 ± 0.4 | 3.6 ± 1.5 | 3.2 ± 2.2 | 2.8 ± 1.8 | 3.1 ± 2.6 | 2.4 ± 1.8 | 3.2 ± 1.5 |
|  |  |  |  | MID | 0.5 ± 0.2 | 2.0 ± 1.7 | 1.7 ± 1.2 | 1.6 ± 1.3 | 2.1 ± 1.6 | 2.2 ± 1.5 | 1.7 ± 1.3 |
|  |  |  |  | FWD | 1.3 ± 0.6 | 4.9 ± 1.5 | 3.7 ± 2.0 | 4.7 ± 3.5 | 3.9 ± 2.3 | 5.8 ± 2.6 | 3.6 ± 2.5 |
| **Interval Between Deceleration (s)** | Mean | Mara et al. (2017) [69] | DOM D1 | All |  | 11 ± 4 | 14 ± 6 | 14 ± 4 | 14 ± 7 | 15 ± 5 | 16 ± 8 |
|  | Maximum |  |  |  |  | 71 ± 22 | 97 ± 42 | 96 ± 32 | 83 ± 34 | 102 ± 34 | 101 ± 43 |
| **High Metabolic Load Distance (m∙min^-1^)** | >20 W∙kg^-1^ | Williams et al. (2019) [42] | COL D1 | All |  | 23 ± 11 | 20 ± 10 | 20 ± 10 | 19 ± 11 | 19 ± 10 | 21 ± 11 |

Qualitative VT = High-Speed Running “striding; movement is similar to jogging but involves a longer stride and more pronounced arm swing”; Sprinting “maximal effort with a greater extension of the lower leg during forward swing and higher heel lift relative to striding”. NS=not specified. Standard: INT=international; DOM=domestic; UEFA CL=UEFA Champions League. Playing Position: CD=central defender; FB=full-back; WD=wide defender; MID=midfield; WM=wide midfielder; FWD=forward.*mean ± SE. **mean calculated from available data
